# Brønstead Acid-Catalyzed Regiodivergent Hydroindolation of Indoles: Temperature-Controlled Markovnikov and Anti-Markovnikov Addition

**DOI:** 10.3390/ijms26188757

**Published:** 2025-09-09

**Authors:** Asaithampi Ganesan, Yong-Uk Kwon

**Affiliations:** Department of Chemistry and Nanoscience, Ewha Womans University, Seoul 03760, Republic of Korea; drasaithampiganesan@gmail.com

**Keywords:** Brønsted acid, indole, Markovnikov addition, anti-Markovnikov addition, hydroindolation, bis(indolyl)alkanes, *p*-toluenesulfonic acid

## Abstract

Brønsted acid-catalyzed, regiodivergent hydroindolation of indoles with terminal aryl alkynes was developed, affording bis(indolyl)alkanes in good to excellent yields. Systematic investigations revealed that temperature variation plays a key role in determining the regioselectivity of anti-Markovnikov and Markovnikov addition reactions. The reaction proceeds efficiently under transition metal-free conditions in an environmentally benign water/alcohol solvent system, using readily available and inexpensive *p*-toluenesulfonic acid (TsOH) as the catalyst. Control experiments and mechanistic studies support distinct reaction pathways for each regioisomer.

## 1. Introduction

Indole-derived motifs are widely employed in pharmaceuticals, natural products, agrochemicals, and materials science due to their diverse biological activities and unique chemical structures [1,2,3,4,5]. Among various modifications, structural elaboration at the C3 position of indole is a key synthetic challenge and remains a central focus in organic synthesis. The inherent structure of indole, featuring a nitrogen atom within a fused aromatic system, makes it a valuable precursor in synthetic chemistry [6,7,8]. In particular, the C3 position is highly reactive and amenable to various transformations, significantly affecting the biological profiles of resulting compounds [9,10,11,12]. Consequently, the development of efficient strategies for the synthesis of bis(indolyl)alkane derivatives is of great interest. Among several synthetic approaches, the hydroindolation of alkynes has emerged as a simple, efficient, and cost-effective route to access indole-based frameworks [13,14,15,16,17].

**Scheme 1:**
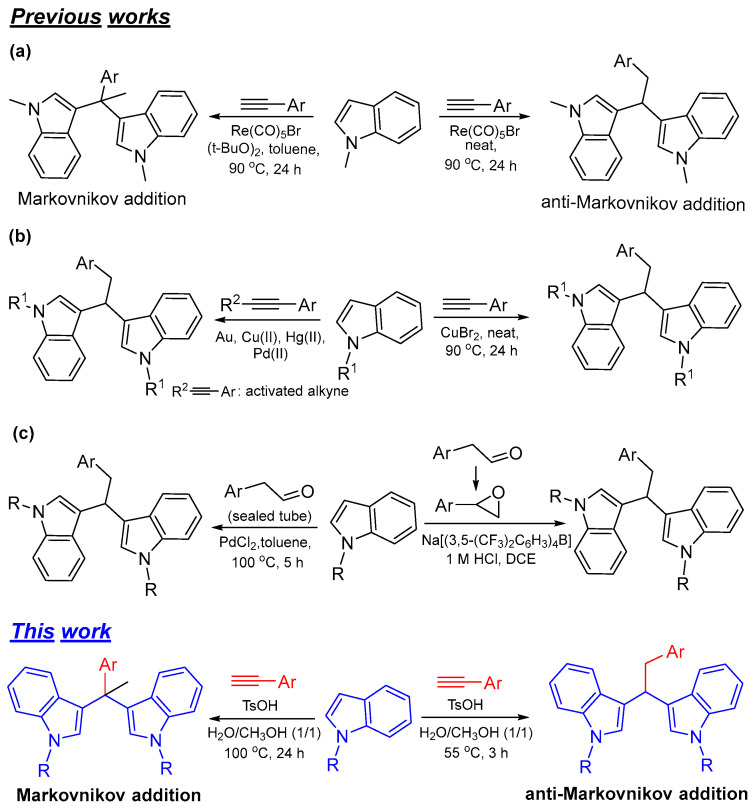
Previous works and this work for Markovnikov and anti-Markovnikov hydroindolations: (**a**) temperature-controlled regioselective hydroindolations [23], (**b**) metal-catalyzed hydroindolations [24,26,28,29,30,31,32], (**c**) hydroindolations using phenylacetaldehyde [25,33,34].

Notably, transition metal-catalyzed additions of indoles to terminal alkynes have been widely explored to construct bis(indolyl)alkane scaffolds via either the Markovnikov (Ga, In, Ag, Au, Pd, Hg, Pt) [18,19,20,21,22] or anti-Markovnikov (Pd, Cu, Au, Re) [23,24,25,26,27] pathways. However, achieving high regioselectivity remains challenging (Scheme 1). The unique electronic properties of metal–alkyne complexes promote double-indole addition at the internal carbon of the alkyne, favoring the Markovnikov adducts [18,19,20,21,22]. In contrast, anti-Markovnikov addition has been investigated through intramolecular hydroindolation in the presence of Au or Hg(OTf)_2_ catalysts, leading to fused indole derivatives [28,29,30].

On the other hand, Barluenga et al. reported an intermolecular anti-Markovnikov addition of *N*-substituted indoles to activated terminal alkynes, directed by hydroxyl groups and catalyzed by gold complexes [31]. Moreover, Xia et al. reported a rhenium-catalyzed anti-Markovnikov addition of *N*-substituted indoles to terminal alkynes in toluene, while Markovnikov addition was achieved under neat conditions. However, this method was ineffective for *NH*-free indole derivatives [23]. In contrast, Jain and co-workers demonstrated a CuBr_2_-catalyzed anti-Markovnikov addition of *NH*-free indoles under neat conditions, but the method was not suitable for *N*-protected indoles [24]. In 2019, Guo et al. explored the PdCl_2_-catalyzed anti-Markovnikov hydroindolation of indole with aldehydes in toluene under sealed-tube conditions [25]. More recently, Tyagi et al. described the borane–Brønsted acid-catalyzed anti-Markovnikov hydroindolation of indoles with styrene oxide [32]. The downside of this approach is the use of aldehyde to produce styrene oxide, as both aldehyde and indole are good candidates for anti-Markovnikov addition in the presence of Lewis and Brønsted acid catalysis [33,34]. However, these methods often suffer from practical limitations, including restricted substrate scope, high cost, use of hazardous solvents, and challenging catalyst preparation. Therefore, developing a robust, inexpensive, and non-toxic catalytic system for synthesizing a variety of 3-substituted indole derivatives under mild and environmentally friendly conditions remains a significant goal. To address the limited efficiency and scope of existing anti-Markovnikov hydroindolation methods for terminal aryl alkynes, we report a transition metal-free catalytic system that enables the regioselective formation of bis(indolyl)alkanes under benign conditions.

We investigated the use of *p*-toluenesulfonic acid (TsOH), a well-known and cost-effective Brønsted acid, as a catalyst for hydroindolation. To the best of our knowledge, TsOH-catalyzed hydroindolation of unactivated alkynes has not been reported. In this study, TsOH was used in a water/methanol mixture, a green solvent system, to develop a simple and economical method for both the Markovnikov and anti-Markovnikov addition of indoles to terminal aryl alkynes. TsOH has previously shown utility in various organic transformations under mild and environmentally benign conditions [35,36].

## 2. Results and Discussion

Indole and phenylacetylene were initially selected as model substrates to optimize the synthesis of bis(indolyl)alkane. The screening results are summarized in Table 1. At room temperature, TsOH (20 mol%) was tested in various polar protic solvents (entries 1–3), where methanol and water showed reasonable conversion within a short time. Interestingly, when the temperature was raised to 55 °C, product formation improved in both solvents (entries 4–5). Notably, using a water/methanol (1/1) mixture at 55 °C afforded the anti-Markovnikov product 3a in 87% yield (entry 6). Further solvent screening (Table S2) showed that polar protic solvents, including alcohols and water, supported the anti-Markovnikov hydroindolation, whereas polar aprotic and non-polar solvents inhibited product formation. Reactions conducted without solvent or without catalyst gave poor results (entries 7–8).

With the optimal solvent system (H_2_O/MeOH) established, various Brønsted acids and additives were evaluated for anti-Markovnikov hydroindolation. Sulfonic acid-based catalysts such as H_2_SO_4_, triflic acid, methanesulfonic acid, and camphorsulfonic acid (entries 9–12) gave lower yields than TsOH. Other acids including HCl, AcOH, and TFA were also ineffective (entries 13–15). Moreover, incorporating Lewis acidic additives under the optimized conditions did not improve the outcome (Table S3). Next, the effect of TsOH loading was investigated. Reducing the catalyst below 20 mol% led to decreased yields (Table S4), while increasing it to 100 mol% offered no further benefit. Importantly, the reaction was highly efficient with 20 mol% TsOH under mild conditions, outperforming previously reported methods involving costly rhenium catalysis [23]. Temperature screening from 25 °C to 100 °C revealed 55 °C as optimal (Table S5). Thus, the standard conditions were set as 2 mmol of indole and 1.5 mmol of phenylacetylene in H_2_O/MeOH (1/1) with 20 mol% TsOH at 55 °C for 3 h.

**Scheme 2:**
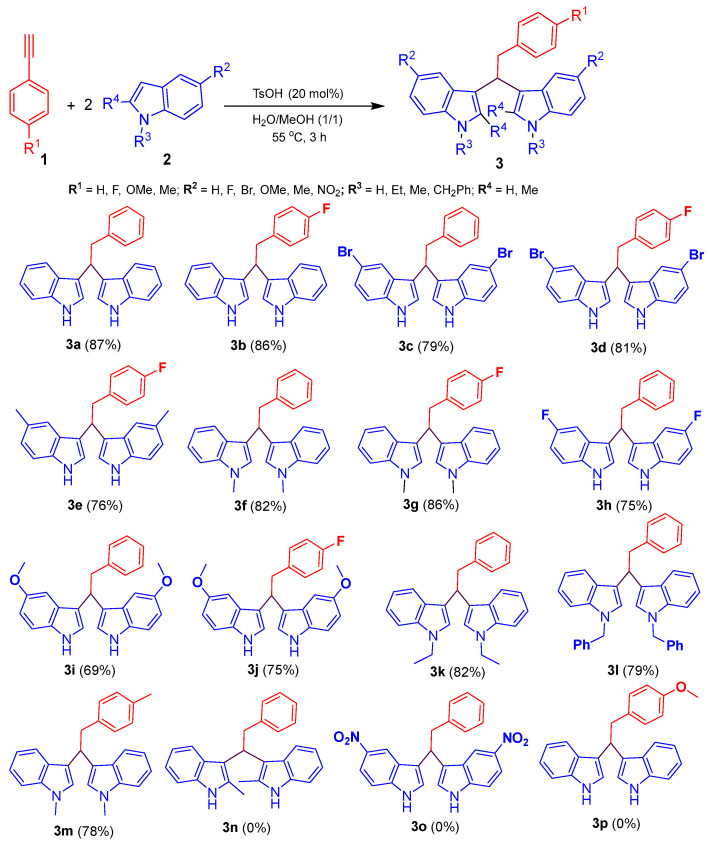
Substrate scope for TsOH-catalyzed anti-Markovnikov hydroindolation.

With the optimized conditions in hand, we explored the substrate scope of the TsOH-catalyzed one-pot hydroindolation using various indoles and terminal alkynes (Scheme 2). Generally, C5-substituted indoles bearing electron-donating or electron-withdrawing groups reacted smoothly with a range of unactivated terminal alkynes, furnishing anti-Markovnikov products in good yields. However, 5-nitroindole failed to react with phenylacetylene, likely due to solubility issues. Similarly, 2-methylindole did not yield the desired product, presumably because of steric hindrance. Despite these limitations, the protocol was broadly applicable to *NH*-free, *N*-substituted, and C5-substituted indoles, affording bis(indolyl)alkanes **3a**–**3m** efficiently. Notably, previous methods showed narrower scope: Xia et al. reported that Re-catalyzed reactions were effective only with *N*-methylindoles [23] while Srivastava et al. showed CuBr_2_-catalyzed reactions compatible with *NH*-free indoles but not with *N*-substituted ones [24]. In contrast, the TsOH-catalyzed strategy accommodates a wide range of indole substrates without the need for transition metal catalysts. Additionally, the reaction tolerated various substituents (H, CH_3_, F) on phenylacetylenes. Interestingly, 4-methoxyphenylacetylene did not react with *NH*-free indole, likely due to the strong resonance-donating (*+R*) effect of the methoxy group, which may have deactivated the alkyne toward electrophilic activation [24].

**Scheme 3:**
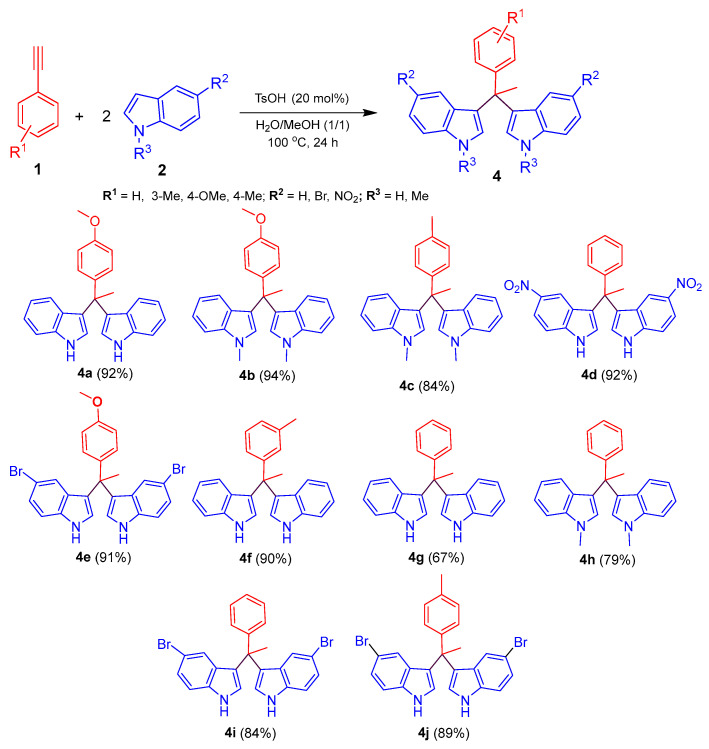
Substrate scope for TsOH-catalyzed Markovnikov hydroindolation.

In addition, when the reaction temperature was raised to 100 °C, the Markovnikov addition product was obtained in a good yield, with no formation of the anti-Markovnikov product—likely due to the *+R* effect. This temperature-dependent regioselectivity was further explored using indole, *N*-methylindole, and C5-substituted indoles bearing electron-donating or electron-withdrawing groups with unactivated alkynes over 24 h at 90–120 °C in the presence of TsOH (Scheme 3). Ultimately, 100 °C was identified as the optimal temperature for TsOH-catalyzed Markovnikov hydroindolation in the water/methanol system.

In our studies on Markovnikov addition to aromatic alkynes, we found that the reaction temperature plays a key role in determining regioselectivity. Remarkably, increasing the temperature shifted the selectivity entirely toward Markovnikov addition, favoring bis(indolyl)alkane formation while completely suppressing anti-Markovnikov products. For example, 5-nitroindole, which gave no anti-Markovnikov product under standard conditions, afforded the Markovnikov product (**4d**) in high yield after heating at 100 °C for 24 h. No traces of anti-Markovnikov products were detected under these conditions. Importantly, both regioisomeric pathways are promoted by the same TsOH catalyst, with temperature serving as the key switch for selectivity. To demonstrate practical utility, gram-scale reactions using 10 mmol of indole with phenylacetylene or 4-ethynylanisole under optimized conditions provided **3a** and **4a** in 82% and 84% yield, respectively (Schemes S3 and S4), confirming the scalability of the method. Additionally, we investigated the hydroindolation of indole using 1-hexyne as a terminal alkyl alkyne. The desired anti-Markovnikov product was detected in the ^1^H-NMR spectrum as part of a complex mixture, but could not be isolated. The crude product from the Markovnikov addition also proved impossible to purify, as TLC revealed multiple unidentifiable spots Therefore, we conclude that our TsOH-catalyzed switchable anti-Markovnikov and Markovnikov hydroindolation is not currently suitable for terminal alkyl-substituted alkynes, although further studies on these substrates are needed.

**Scheme 4:**
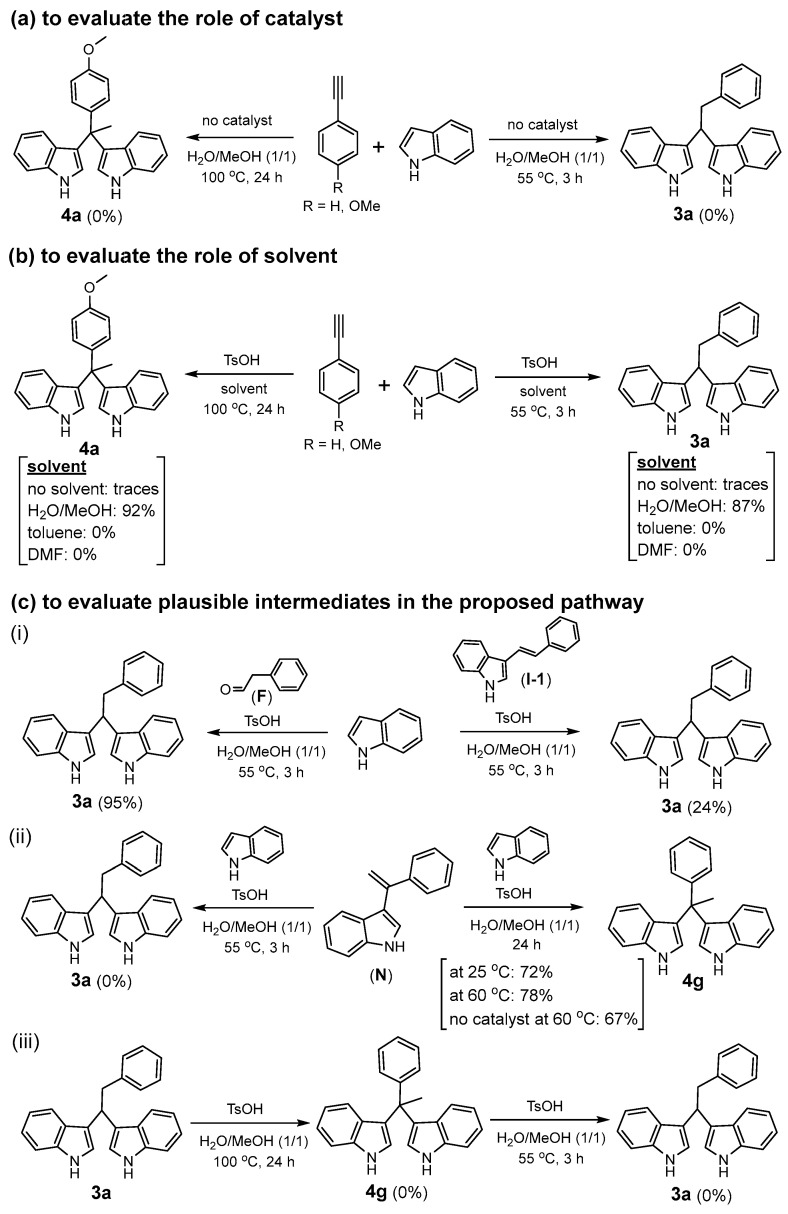
Control experiments for mechanistic studies.

A series of control experiments were conducted to better understand the reaction mechanism (Scheme 4). First, both hydroindolation reactions were performed without a catalyst, and no product formation was observed (Scheme 4a), confirming the essential role of TsOH. When reactions were carried out neat with TsOH, only trace amounts of product were obtained (Scheme 4b). Subsequently, to probe the solvent effect further, reactions were tested in toluene and DMF, but no product formation occurred, indicating that polar protic solvents are crucial. These solvent screenings demonstrate that TsOH-induced hydroindolation proceeds exclusively in the presence of H_2_O/MeOH. These studies clearly indicate that polar protic solvents are essential for achieving both anti-Markovnikov and Markovnikov selectivity, depending on the reaction temperature and duration.

To investigate the reaction mechanism, we examined potential intermediates based on the proposed pathway. Phenylacetaldehyde (**F**) and (*E*)-3-styryl-1*H*-indole (**I-1**) [24] were identified as plausible intermediates (Scheme 4) [33,36]. When each was independently reacted with indole under optimized conditions, the anti-Markovnikov product **3a** was obtained (Scheme 4c-i), supporting their roles in the reaction sequence. However, this pathway appeared less effective for the Markovnikov process. Treating **I-1** with indole at 55 °C gave **3a** in only 24% yield, while heating to 100 °C for 24 h produced unidentified byproducts. Separately, the reaction of indole with 3-(1-phenylvinyl)-1*H*-indole (**N**) led selectively to the Markovnikov product **4g** (Scheme 4c-ii). In contrast, subjecting **3a** to TsOH in water/methanol at 100 °C for 24 h resulted in unidentified byproducts (Scheme 4c-iii). Similarly, **4g** was unreactive in the presence of TsOH at 55 °C for 3 h and did not convert into **3a**. These results indicate that the Markovnikov and the anti-Markovnikov addition products are not interconverted under the reaction conditions, suggesting that their formation proceeds through independent mechanistic pathways. Temperature-controlled regioselectivity can be achieved through distinct, independent reaction pathways originating from the same starting materials. In 2022, Milián et al. reported BCl_3_-catalyzed, temperature-controlled regiodivergent borylative cyclizations of enynes [37]. Likewise, Wang et al. reported a switchable, site-selective hydroalkylation that affords nitrogenous α- and β-alkylated products with different skeletal structures from the same alkene substrates by temperature regulation [38]. These regioselectivities were rationalized by the substantial differences in the Gibbs free energy barriers of the respective intermediates. In a similar manner, our study indicates that the thermodynamic and kinetic properties of intermediates, as reflected in their Gibbs free energy barriers, may play a key role in governing the switchable site selectivity between the Markovnikov and anti-Markovnikov addition products.

**Scheme 5:**
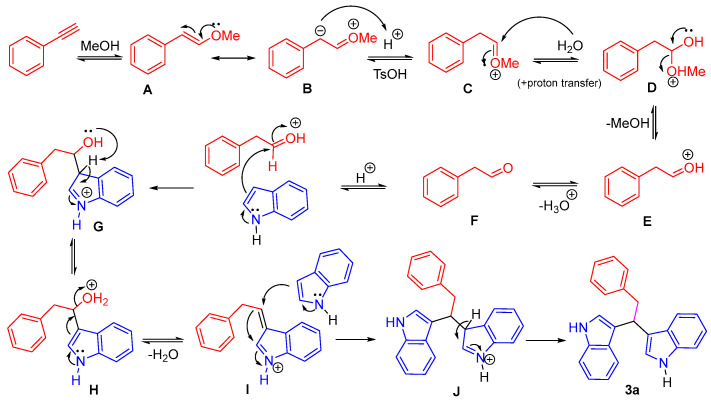
Plausible reaction mechanism for anti-Markovnikov addition.

Based on control experiments and prior reports [39,40,41,42], a plausible reaction mechanism for the anti-Markovnikov addition is proposed (Scheme 5). Initially, methanol reacts with phenylacetylene to form intermediate **A**. The oxygen atom in intermediate **A** donates its lone pair of electrons to generate resonance structure **B**, which then undergoes protonation to afford intermediate **C**. Subsequent nucleophilic attack by H_2_O on intermediate **C** forms intermediate **E**, accompanied by the elimination of methanol. The protonated form of phenylacetaldehyde (**F**) then reacts with indole via nucleophilic addition and dehydration to give azafulvene intermediate **I**. Finally, a second indole molecule subsequently adds to **I**, affording the anti-Markovnikov product **3a** [33].

**Scheme 6:**
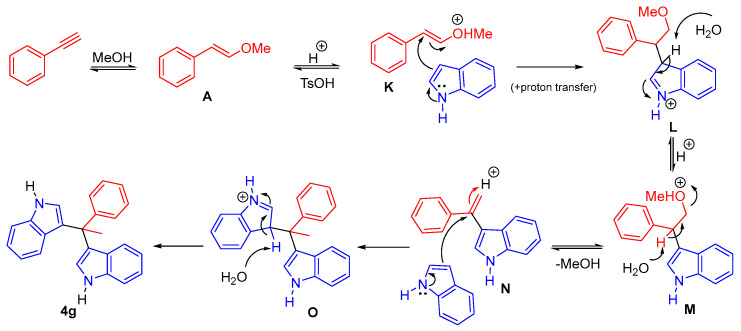
Plausible reaction mechanism for Markovnikov addition.

Based on control studies and prior reports [39,40,41,42], a plausible reaction mechanism for TsOH-catalyzed Markovnikov hydroindolation is proposed (Scheme 6). The reaction begins with nucleophilic addition of indole to intermediate **K**, the less stable protonated form of intermediate **A**. This is followed by a deprotonation–protonation sequence, affording 3-(1-phenylvinyl)-1*H*-indole (**N**) with elimination of methanol [42,43,44,45,46,47,48]. Subsequently, a second indole molecule then undergoes TsOH-facilitated nucleophilic addition to intermediate **N**, yielding the Markovnikov product.

**Scheme 7:**
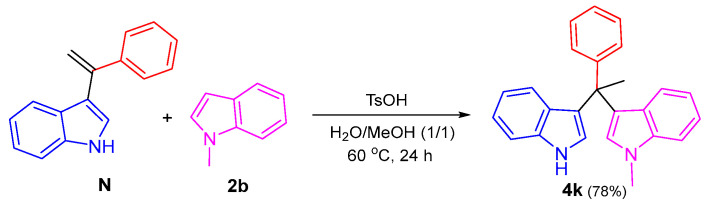
Synthesis of asymmetric Markovnikov hydroindolation product.

Furthermore, the asymmetric Markovnikov addition between intermediate **N** [49] and *N*-methylindole afforded the expected product **4k** in 78% yield (Scheme 7), further supporting the above proposed mechanism.

## 3. Material and Methods

Unless otherwise stated, all reactions were carried out in an open-air atmosphere under the specified optimized reaction conditions. Methanol and other solvents were obtained from Sigma-Aldrich (Merk: Darmstadt, Germany) and used without further purification. Starting materials, including phenylacetylene and indole derivatives, were supplied by TCI (Tokyo, Japan). *p*-Toluenesulfonic acid monohydrate (TsOH) was also obtained from Sigma-Aldrich and employed in all processes without additional purification. Merck TLC 60 F254 silica gel plates were used for thin-layer chromatography (TLC) and visualized under brief UV light exposure. For purification, silica gel (230–400 mesh) was used with dichloromethane (DCM), hexane, and methanol as the mobile phases. All compounds were fully characterized by **^1^H** and **^13^C** nuclear magnetic resonance (NMR) spectra which were recorded on a 300 MHz Bruker Avance spectrometer (Billerica, MA, USA) at the National Research Facilities and Equipment Center (NanoBio·Energy Materials Center) at Ewha Womans University (Seoul, Korea), using CDCl_3_, DMSO-d_6_, and acetone-d_6_ as solvents. Chemical shift values (δ) are reported in parts per million (ppm).

### 3.1. General Procedure for Anti-Markovnikov Hydroindolation

To a stirred solution of indole (234 mg, 2 mmol) and phenylacetylene (153 mg, 1.5 mmol) in a water/methanol (1 mL/1 mL) mixture, *p*-toluenesulfonic acid monohydrate (70 mg, 20 mol%) was added. The reaction temperature was then heated to 55 °C and stirred for 3 h. Upon completion, the reaction mixture was cooled to room temperature and extracted with dichloromethane (DCM), followed by washing with brine solution. The resulting organic layer was dried over anhydrous Na_2_SO_4_ and concentrated under reduced pressure. The crude product was purified by silica gel column chromatography using a 20% DCM/hexane eluent, affording the pure anti-Markovnikov addition product **3a** (293 mg, 87%). All the synthesized compounds were previously reported and characterized by ^1^H and ^13^C NMR spectroscopy (Supplementary Materials).

### 3.2. General Procedure for Markovnikov Hydroindolation

To a stirred solution of indole (234 mg, 2 mmol) and phenylacetylene (153 mg, 1.5 mmol) in a water/methanol (1 mL/1 mL) mixture, *p*-toluenesulfonic acid monohydrate (70 mg, 20 mol%) was added. The reaction mixture was then heated to 100 °C and stirred for 24 h. Upon completion, the reaction mixture was cooled to room temperature, extracted with DCM, and washed with brine. The organic layer was dried over anhydrous Na_2_SO_4_ and concentrated under reduced pressure. The crude product was purified using 10% DCM-hexane eluent on silica gel column chromatography, affording the pure Markovnikov addition product **4g** (225 mg, 67%). All the synthesized compounds were previously reported and characterized by ^1^H and ^13^C NMR spectroscopy (Supplementary Materials).

## 4. Conclusions

In conclusion, Brønsted acid catalysis using TsOH enabled the highly regioselective anti-Markovnikov addition of indoles to terminal aryl alkynes at 55 °C. Remarkably, raising the temperature to 100 °C switched the selectivity toward Markovnikov products with excellent efficiency. Both electron-donating and electron-withdrawing substituents on the indole or phenylacetylene rings were well tolerated, with each addition pathway proceeding with high reactivity and outstanding selectivity across the tested temperature range. Additionally, the protocol was compatible with both *NH*-free and *N*-substituted indoles, enabling the synthesis of a broad array of regioselective bis(indolyl)alkanes in good to excellent yields. Based on the control experiments, plausible mechanisms for both anti-Markovnikov and Markovnikov hydroindolation have been proposed. Further studies are underway to develop asymmetric variants using broader nucleophile classes.

## Figures and Tables

**Table 1:** Optimization of reaction conditions for Brønsted acid-catalyzed anti-Markovnikov hydroindolation ^a^.

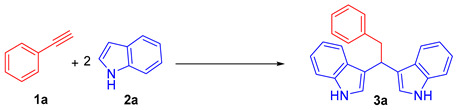				
Entry	Catalyst ^b^	Solvent ^c^	Temp (°C)/Time (h)	Yield (%) ^d^
1	TsOH	MeOH	rt/12	74
2	TsOH	H_2_O	rt/12	71
3	TsOH	EtOH	rt/12	53
4	TsOH	MeOH	55/3	83
5	TsOH	H_2_O	55/3	79
6	TsOH	H_2_O/MeOH	55/3	87
7	TsOH	-	55/3	ta
8	-	H_2_O/MeOH	55/24	nr
9	con. H_2_SO_4_	H_2_O/MeOH	55/3	64
10	CF_3_SO_3_H	H_2_O/MeOH	55/3	52
11	MsOH	H_2_O/MeOH	55/3	46
12	CSA	H_2_O/MeOH	55/3	59
13	con. HCl	H_2_O/MeOH	55/3	ta
14	CH_3_COOH	H_2_O/MeOH	55/3	nr
15	CF_3_COOH	H_2_O/MeOH	55/3	nr

^a^ Reaction conditions: **1a** (1.5 mmol), **2a** (2.0 mmol), catalyst (20 mol%). ^b^ TsOH: *p*-toluenesulfonic acid monohydrate, MsOH: methanesulfonic acid, CSA: camphorsulfonic acid. ^c^ H_2_O/MeOH: 1/1 mixture. ^d^ Isolated yield, ta: trace amount, nr: no reaction.

## Data Availability

The data are included in the article or the Supplementary Materials. Additional data from this study are available upon request from the authors.

## Supplementary Materials

The following supporting information can be downloaded at: https://www.mdpi.com/article/10.3390/ijms26188757/s1.

## References

[1] Shiri M., Zolfigol M.A., Kruger H.G., Tanbakouchian Z. (2010). Bis- and Trisindolylmethanes (BIMs and TIMs). Chem. Rev..

[2] Lysenko V., Machushynets N.V., van Dam J.L., Sterk F.A.C., Speer A., Ram A.F.J., Slingerland C.J., Van Wezel G.P., Martin N.I. (2025). Total Synthesis, Structure Elucidation, and Bioactivity Evaluation of the Cyclic Lipopeptide Natural Product Paenilipoheptin, A. Org. Lett..

[3] Frecentese F., Sodano F., Corvino A., Schiano M.E., Magli E., Albrizio S., Sparaco R., Andreozzi G., Nieddu M., Rimoli M.G. (2023). The Application of Microwaves, Ultrasounds, and Their Combination in the Synthesis of Nitrogen-Containing Bicyclic Heterocycles. Int. J. Mol. Sci..

[4] Khalilullah H., Agarwal D.K., Ahsan M.J., Jadav S.S., Mohammed H.A., Khan M.A., Mohammed S.A.A., Khan R. (2022). Synthesis and Anti-Cancer Activity of New Pyrazolinyl-Indole Derivatives: Pharmacophoric Interactions and Docking Studies for Identifying New EGFR Inhibitors. Int. J. Mol. Sci..

[5] Silva P., de Almeida M., Silva J., Albino S., Espírito-Santo R., Lima M., Villarreal C., Moura R., Santos V. (2020). (E)-2-Cyano-3-(1H-Indol-3-Yl)-N-Phenylacrylamide, a Hybrid Compound Derived from Indomethacin and Paracetamol: Design, Synthesis and Evaluation of the Anti- Inflammatory Potential. Int. J. Mol. Sci..

[6] Olyaei A., Sadeghpour M. (2023). Chemistry of 3-Cyanoacetyl Indoles: Synthesis, Reactions and Applications: A Recent Update. RSC Adv..

[7] Chen J.B., Jia Y.X. (2017). Recent Progress in Transition-Metal-Catalyzed Enantioselective Indole Functionalizations. Org. Biomol. Chem..

[8] Lőrinczi B., Simon P., Szatmári I. (2022). Synthesis of Indole-Coupled KYNA Derivatives via C–N Bond Cleavage of Mannich Bases. Int. J. Mol. Sci..

[9] Wang S.Y., Shi X.C., Laborda P. (2020). Indole-Based Melatonin Analogues: Synthetic Approaches and Biological Activity. Eur. J. Med. Chem..

[10] Salami S.A., Smith V.J., Krause R.W.M. (2023). Aqua/Mechanochemical Mediated Synthesis of Novel Spiro [Indole–Pyrrolidine] Derivatives. Int. J. Mol. Sci..

[11] Sorokin B., Filimonova A., Emelianova A., Kublitski V., Gvozd A., Shmygarev V., Yampolsky I., Guglya E., Gusev E., Kuzmin D. (2025). Novel Triazeneindole Antibiotics: Synthesis and Hit-to-Lead Optimization. Int. J. Mol. Sci..

[12] Wróbel M., Chodkowski A., Siwek A., Satała G., Bojarski A.J., Dawidowski M. (2024). Design and Synthesis of Potential Multi-Target Antidepressants:_Exploration of 1-(4-(7-Azaindole)-3,6-dihydropyridin-1-yl)alkyl-3-(1*H*-indol-3-yl)pyrrolidine-2,5-dione Derivatives with Affinity for the Serotonin Transporter. Int. J. Mol. Sci..

[13] Ma J., Feng R., Dong Z.B. (2023). Recent Advances in Indole Synthesis and the Related Alkylation. Asian J. Org. Chem..

[14] Ferrer C., Amijs C.H.M., Echavarren A.M. (2007). Intra- and Intermolecular Reactions of Indoles with Alkynes Catalyzed by Gold. Chem. Eur. J..

[15] Xu Y., Liang X., Hyun C.G. (2024). Discovery of Indole–Thiourea Derivatives as Tyrosinase Inhibitors: Synthesis, Biological Evaluation, Kinetic Studies, and In Silico Analysis. Int. J. Mol. Sci..

[16] Al-Wabli R.I., Issa I.S., Al-mutairi M.S., Almomen A.A., Attia M.I. (2023). A Facile Synthesis and Molecular Characterization of Certain New Anti-Proliferative Indole-Based Chemical Entities. Int. J. Mol. Sci..

[17] Oberhuber N., Ghosh H., Nitzsche B., Dandawate P., Höpfner M., Schobert R., Biersack B. (2023). Synthesis and Anticancer Evaluation of New Indole-Based Tyrphostin Derivatives and Their (*p*-Cymene)dichloridoruthenium(II) Complexes. Int. J. Mol. Sci..

[18] Yadav J.S., Reddy B.V.S., Padmavani B., Gupta M.K. (2004). Gallium(III) Halide-Catalyzed Coupling of Indoles with Phenylacetylene: Synthesis of Bis(indolyl)phenylethanes. Tetrahedron Lett..

[19] Luo C., Yang H., Mao R., Lu C., Cheng G. (2015). An Efficient Au(i) Catalyst for Double Hydroarylation of Alkynes with Heteroarenes. New J. Chem..

[20] Maiti G., Kayal U., Karmakar R., Bhattacharya R.N. (2013). An Efficient One Pot Conversion of Alkynes to Bis(indolyl) and Bis(pyrrolyl)alkanes in Aqueous Ethanol. Indian J. Chem. Sect. B Org. Med. Chem..

[21] Xie M.H., Xie F.D., Lin G.F., Zhang J.H. (2010). Convenient Synthesis of Bis(indolyl)alkanes and Bis(pyrrolyl)alkanes by Cu(OTf)_2_-Catalyzed Addition of Indole and Pyrrole to Acetylenic Sulfone. Tetrahedron Lett..

[22] Baron M., Biffis A. (2019). Gold(I) Complexes in Ionic Liquids: An Efficient Catalytic System for the C-H Functionalization of Arenes and Heteroarenes under Mild Conditions. Eur. J. Org. Chem..

[23] Xia D., Wang Y., Du Z., Zheng Q.Y., Wang C. (2012). Rhenium-Catalyzed Regiodivergent Addition of Indoles to Terminal Alkynes. Org. Lett..

[24] Srivastava A., Patel S.S., Chandna N., Jain N. (2016). Copper-Catalyzed Anti-Markovnikov Hydroindolation of Terminal Alkynes: Regioselective Synthesis of Bis(indolyl)alkanes. J. Org. Chem..

[25] Guo S., Fang Z., Zhou B., Hua J., Dai Z., Yang Z., Liu C., He W., Guo K. (2019). Cu/Pd-Catalyzed Chemoselective Synthesis of C-3 Dicarbonyl Indoles and Bis(indolyl)alkanes from Aldehydes and Indoles. Org. Chem. Front..

[26] Li Z., Shi Z., He C. (2005). Addition of Heterocycles to Electron Deficient Olefins and Alkynes Catalyzed by Gold(III). J. Organomet. Chem..

[27] Lu W., Jia C., Kitamura T., Fujiwara Y. (2000). Pd-Catalyzed Selective Addition of Heteroaromatic C-H Bonds to C-C Triple Bonds under Mild Conditions. Org. Lett..

[28] Ferrer C., Escribano-Cuesta A., Echavarren A.M. (2009). Synthesis of the Tetracyclic Core Skeleton of the Lundurines by a Gold-Catalyzed Cyclization. Tetrahedron.

[29] Ferrer C., Echavarren A.M. (2006). Gold-Catalyzed Intramolecular Reaction of Indoles with Alkynes: Facile Formation of Eight-Membered Rings and an Unexpected Allenylation. Angew. Chem. Int. Ed..

[30] Donets P.A., Van Hecke K., Van Meervelt L., Van Der Eycken E.V. (2009). Efficient Synthesis of the Indoloazocine Framework via Intramolecular Alkyne Carbocyclization. Org. Lett..

[31] Barluenga J., Fernández A., Rodríguez F., Fañanás F.J. (2009). Synthesis of Bis(indolyl)alkanes by a Site-Selective Gold-Catalyzed Addition of Indoles to Butynol Derivatives. J. Organomet. Chem..

[32] Tyagi A., Khan J., Yadav N., Mahato R., Hazra C.K. (2022). Catalyst-Switchable Divergent Synthesis of Bis(indolyl)alkanes and 3- Alkylated Indoles from Styrene Oxides. J. Org. Chem..

[33] Subramaniapillai S.G., Ganesan A. (2014). ZnCl_2_ Promoted Efficient, One-Pot Synthesis of 3-Arylmethyl and Diarylmethyl Indoles. Tetrahedron Lett..

[34] Banari H., Kiyani H., Pourali A.R. (2019). Bisindolization Reaction Employing Phthalimide-N-Sulfonic Acid as an Efficient Catalyst. Curr. Organocatalysis.

[35] Liu P., Pan Y.M., Xu Y.L., Wang H.S. (2012). PTSA-Catalyzed Mannich-Type-Cyclization-Oxidation Tandem Reactions: One-Pot Synthesis of 1,3,5-Substituted Pyrazoles from Aldehydes, Hydrazines and Alkynes. Org. Biomol. Chem..

[36] Yadav A., Dahiya P., Rawat M., Peddinti R.K. (2024). PTSA-Catalyzed [3 + 2] Cycloaddition of in Situ Generated 3-Ylidene Oxindoles with Coumarin-Based N-Phenyl Enamine Derivatives. Eur. J. Org. Chem..

[37] Milián A., Fernández-Rodríguez M.A., Merino E., Vaquero J.J., García-García P. (2022). Metal-Free Temperature-Controlled Regiodivergent Borylative Cyclizations of Enynes: BCl_3_-Promoted Skeletal Rearrangement. Angew. Chem. Int. Ed..

[38] Wang J.W., Liu D.G., Chang Z., Li Z., Fu Y., Lu X. (2022). Nickel-Catalyzed Switchable Site-Selective Alkene Hydroalkylation by Temperature Regulation. Angew. Chem. Int. Ed..

[39] Zhu W., Tang N., Zhou W., Li C., Ou Y., Abdukader A., Qiu R. (2024). Base-Promoted Annulated Aromatization of Aryl Acetylenes with Dimethyl Sulfoxide to Access Symmetrical/Unsymmetric M- Terphenyl. J. Org. Chem..

[40] Huang W.J., Liu L.X., Zhou Y.G., Wu B., Jiang G.F. (2023). Brønsted Acid-Catalyzed C6 Functionalization of 2,3-Disubstituted Indoles for Construction of Cyano-Substituted All-Carbon Quaternary Centers. Org. Biomol. Chem..

[41] Hermange P., Gicquiaud J., Barbier M., Karnat A., Toullec P.Y. (2022). Bronsted Acid Catalyzed Carbocyclizations Involving Electrophilic Activation of Alkynes. Synthesis.

[42] Otani T., Ueki K., Cho K., Kanai K., Tateno K., Saito T. (2015). Construction of Dibenzo-Fused Seven-to Nine-Membered Carbocycles via Bronsted Acid-Promoted Intramolecular Friedel–Crafts-Type Alkenylation. Chem. Commun..

[43] Ling F., Xiao L., Fang L., Feng C., Xie Z., Lv Y., Zhong W. (2018). B(C_6_F_5_)_3_-Catalyzed Markovnikov Addition of Indoles to Aryl Alkynes: An Approach toward Bis(indolyl)alkanes. Org. Biomol. Chem..

[44] Dipika N., Sharma Y.B., Pant S., Dhaked D.K., Guru M.M. (2022). Borane-Catalyzed Dehydrogenative C-C Bond Formation of Indoles with N-Tosylhydrazones: An Experimental and Computational Study. Org. Chem. Front..

[45] Schießl J., Rudolph M., Hashmi A.S.K. (2017). The Gold-Catalyzed Hydroarylation of Alkynes with Electron-Rich Heteroarenes*—*A Kinetic Investigation and New Synthetic Possibilities. Adv. Synth. Catal..

[46] Noland W.E., Brown C.D., DeKruif R.D., Lanzatella N.P., Gao S.M., Zabronsky A.E., Tritch K.J. (2018). Condensation Reactions of Indole with Acetophenones Affording Mixtures of 3,3-(1-Phenylethane-1,1-diyl)bis(1*H*-indoles) and 1,2,3,4-Tetrahydro-3-(1*H*-indol-3-yl)-1-methyl-1,3-diphenylcyclopent[b]indoles. Synth. Commun..

[47] Nair V.N., Kojasoy V., Laconsay C.J., Kong W.Y., Tantillo D.J., Tambar U.K. (2021). Catalyst-Controlled Regiodivergence in Rearrangements of Indole-Based Onium Ylides. J. Am. Chem. Soc..

[48] Kumar S., Rastogi S.K., Singh A., Bharati Ahirwar M., Deshmukh M.M., Sinha A.K., Kumar R. (2022). Friedel-Crafts-Type Reaction of (Het)arenes with Aldehydes/Ketones under Acid-Free Conditions Using Neutral Ionic Liquid: A Convenient Routes to Bis(indolyl)methanes and Beyond. Asian J. Org. Chem..

[49] Wang Z., Ai F., Wang Z., Zhao W., Zhu G., Lin Z., Sun J. (2015). Organocatalytic Asymmetric Synthesis of 1,1-Diarylethanes by Transfer Hydrogenation. J. Am. Chem. Soc..

