# Supplementary material for: Brønstead Acid-Catalyzed Regiodivergent Hydroindolation of Indoles: Temperature-Controlled Markovnikov and Anti-Markovnikov Addition
Source: Int J Mol Sci. 2025 Sep 9;26(18):8757. doi: 10.3390/ijms26188757 (PMC12469531; doi:10.3390/ijms26188757)
Supplement: Supplementary file 1 [file ijms-26-08757-s001.zip › ijms-3846998-supplementary.pdf]

Supplementary Materials

Brønsted Acid-Catalyzed Regiodivergent Hydroindolation of Indoles: Temperature-Controlled Markovnikov and Anti-Markovnikov Addition

Asaithampi Ganesan and Yong-Uk Kwon*

Department of Chemistry and Nanoscience, Ewha Womans University, Seoul 03760, Republic of Korea

*Correspondence: yukwon@ewha.ac.kr

Contents

1. General information 2
2. Optimization of reaction conditions for anti-Markovnikov hydroindolation 2
3. General procedure for anti-Markovnikov hydroindolation 4
  3.1. NMR spectral data of compounds for anti-Markovnikov hydroindolation 5
4. General procedure for Markovnikov hydroindolation 9
  4.1. NMR spectral data of compounds for Markovnikov hydroindolation 10
5. General procedure for gram-scale synthesis 13
6. Control experiments for mechanistic studies 14
  6.1. General procedure for anti-Markovnikov addition via phenylacetaldehyde 14
  6.2. General procedure for anti-Markovnikov addition via (*E*)-3-styryl-1*H*-indole 15
  6.3. General procedure for 3-(1-phenyl-vinyl)-1*H*-indole intermediate 15
  6.4. General procedure for Markovnikov addition via 3-(1-phenyl-vinyl)-1*H*-indole 16
7. NMR spectral data of compounds for asymmetric Markovnikov addition 17
8. References 17
9. NMR spectra 18

1. General information

Unless otherwise stated, all reactions were carried out in an open-air atmosphere under the specified optimized reaction conditions. Methanol and other solvents were obtained from Sigma-Aldrich and used without further purification. Starting materials, including phenylacetylene and indole derivatives, were supplied by TCI chemicals. *p*-Toluenesulfonic acid monohydrate (TsOH·H_2O) was also obtained from Sigma-Aldrich and employed in all processes without additional purification. Merck TLC 60 F254 silica gel plates were used for thin-layer chromatography (TLC) and visualized under brief UV light exposure. For purification, silica gel (230–400 mesh) was used with dichloromethane (DCM), hexane, and methanol as the mobile phases. All compounds were fully characterized by ^{1}H and ^{13}C nuclear magnetic resonance (NMR) spectra which were recorded on a Bruker 300 MHz spectrometer at the National Research Facilities and Equipment Center (NanoBio·Energy Materials Center) at Ewha Womans University, using $CDCl_3$, DMSO-d_6, and acetone-d_6 as solvents. Chemical shift values (δ) are reported in parts per million (ppm).

2. Optimization of reaction conditions for anti-Markovnikov hydroindolation

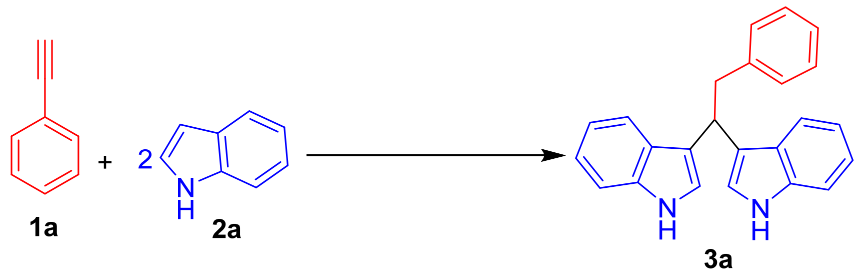

Table S1. Optimization of Brønstead acid catalyst[a]

entry	catalyst	solvent	time (h)	yield (%)
1	TsOH	H_2O	12/24	71/75
2	con. H_2SO_4	H_2O	12/24	67/72
3	CH_3SO_3H	H_2O	12/24	49/56
4	CF_3SO_3H	H_2O	12/24	54/55
5	CSA	H_2O	12/24	62/71

[a] conditions: **1a** (1.5 mmol), **2a** (2.0 mmol), catalyst (20 mol%), temp: rt

Table S2. Optimization of solvent[a]

entry	TsOH (mol%)	solvent	temp. (°C)/time (h)	yield (%)
1	20	MeOH	55/3	83
2	20	EtOH	55/3	64
3	20	CH_3CN	55/3	45
4	20	iPrOH	55/3	20
5	20	1-PrOH	55/3	75
6	20	H_2O	55/3	71
7	20	DMF	55/3	NR[b]
8	20	Et_2O	55/3	NR[b]
9	20	DCM	55/3	NR[b]
10	20	DCE	55/3	NR[b]
11	20	$CHCl_3$	55/3	NR[b]
12	20	PhMe	55/3	NR[b]
13	20	Dioxane	55/3	NR[b]

[a] conditions: **1a** (1.5 mmol), **2a** (2.0 mmol); [b] NR = no reaction

Table S3. Effect of Lewis acidic additives[a]

entry	catalyst	additive	solvent	temp. (°C)/time (h)	yield (%)
1	TsOH	$ZnCl_2$	H_2O/MeOH	55/3	81
2	TsOH	$CuSO_4$	H_2O/MeOH	55/3	72
3	TsOH	AgOTf	H_2O/MeOH	55/3	76
4	TsOH	$Cu(OTf)_2$	H_2O/MeOH	55/3	69
5	TsOH	$InCl_3$	H_2O/MeOH	55/3	82
6	TsOH	$HfCl_4$	H_2O/MeOH	55/3	79

[a] conditions: **1a** (1.5 mmol), **2a** (2.0 mmol), catalyst (20 mol%), additive (10 mol%)

Table S4. Optimization of catalyst loading capacity[a]

entry	TsOH (mol %)	solvent	temp. (°C)/time (h)	yield (%)
1	5	H_2O/MeOH	55/3	40
2	10	H_2O/MeOH	55/3	72
3	15	H_2O/MeOH	55/3	79
4	20	H_2O/MeOH	55/3	87
5	30	H_2O/MeOH	55/3	87
6	40	H_2O/MeOH	55/3	85
7	50	H_2O/MeOH	55/3	85
8	60	H_2O/MeOH	55/3	87
9	70	H_2O/MeOH	55/3	86
10	80	H_2O/MeOH	55/3	85
11	90	H_2O/MeOH	55/3	85
12	100	H_2O/MeOH	55/3	85

[a] conditions: **1a** (1.5 mmol), **2a** (2.0 mmol)

Table S5. Optimization of reaction temperature[a]

entry	TsOH (mol %)	solvent	temp. (°C)/time (h)	yield (%)
1	20	H_2O/MeOH	25/3	15
2	20	H_2O/MeOH	35/3	20
3	20	H_2O/MeOH	45/3	34
4	20	H_2O/MeOH	55/3	87
5	20	H_2O/MeOH	65/3	54
6	20	H_2O/MeOH	75/3	32
7	20	H_2O/MeOH	85/3	20
8	20	H_2O/MeOH	100/3	traces

[a] conditions: **1a** (1.5 mmol), **2a** (2.0 mmol)

3. General procedure for anti-Markovnikov hydroindolation

To a stirred solution of various indole (2 mmol) and terminal alkynes (1.5 mmol) in water/methanol (1/1, 2 mL) as a solvent, *p*-toluenesulfonic acid monohydrate (70 mg, 20 mol%) was added, and the reaction temperature was raised to 55 °C for 3 h. After the reaction was completed, it was cooled, extracted with dichloromethane (DCM) and water, and then washed with brain. The resulting organic layer was dried over anhydrous Na_2SO_4, and the organic layer was concentrated under reduced pressure. After obtaining a crude product, it was purified using 20% of DCM-hexane eluent on silica gel column to give a pure desired product.

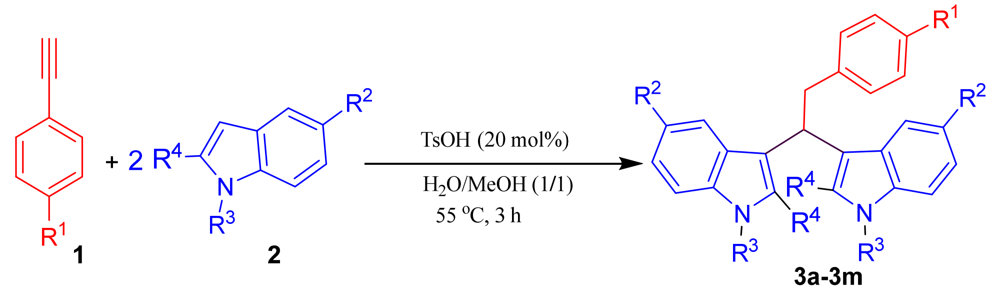

R¹ = H, F, OMe, Me; **R²** = H, F, Br, OMe, Me, NO_2; **R³** = H, Et, Me, CH_2Ph; **R⁴** = H, Me

Scheme S1. Synthesis of anti-Markovnikov hydroindolation of various indoles and terminal aryl alkynes

3.1. NMR spectral data of compounds for anti-Markovnikov hydroindolation

All the synthesized compounds were previously reported and analyzed using ^{1}H and ^{13}C NMR, as well as melting point.

3,3'-(2-phenylethane-1,1-diyl)bis(1*H*-indole) (3a)

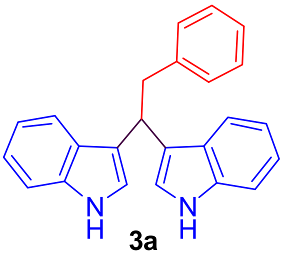

3a

Off-white semi-solid;[1] Yield: 293 mg (87%); ^{1}H NMR (300 MHz, $CDCl_3$) δ 7.90 (s, 2H), 7.51 (d, *J* = 7.8 Hz, 2H), 7.31 (t, *J* = 8.1Hz, 3H), 7.14-7.19 (m, 2H), 6.97-7.04 (m, 6H), 6.56-7.68 (m, 2H), 4.88 (t, *J* = 7.3 Hz, 1H), 3.44 (d, *J* = 7.3 Hz, 2H); ^{13}C NMR (75 MHz, $CDCl_3$) δ 144.7, 136.5, 130.3, 127.0, 126.9, 126.1, 122.1, 121.9, 119.6, 119.4, 119.1, 118.9, 115.8, 111.3, 37.1, 34.2.

3,3'-(2-(4-fluorophenyl)ethane-1,1-diyl)bis(1*H*-indole) (3b)

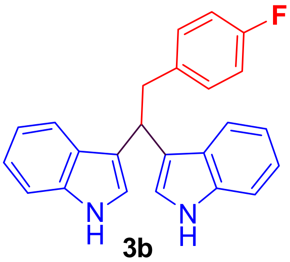

3b

Off-white semi-solid;[1] Yield: 304 mg (86%); ^{1}H NMR (300 MHz, $CDCl_3$) δ 7.83 (s, 2H), 7.52 (d, *J* = 8.01 Hz, 2H), 7.30 (t, *J* = 8.1Hz, 2H), 7.14-7.19 (m, 2H), 6.95-7.05 (m, 6H), 6.56-7.69 (m, 2H), 4.88 (t, *J* = 7.3 Hz, 1H), 3.43 (d, *J* = 7.3 Hz, 2H); ^{13}C NMR (75 MHz, $CDCl_3$) δ 144.7, 136.5, 130.4, 126.94, 126.90, 126.1, 122.0, 121.9, 119.6, 119.5, 111.2, 37.1, 34.3.

3,3'-(2-phenylethane-1,1-diyl)bis(5-bromo-1*H*-indole) (3c)

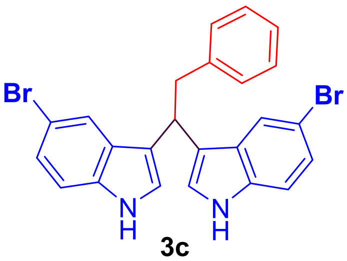

3c

Light brown semi solid;[2] Yield: 388 mg (79%); ^{1}H NMR (300 MHz, $CDCl_3$) δ 8.02 (s, 2H), 7.46 (t, *J* = 0.8 Hz, 2H), 7.19-7.30 (m, 5H), 6.96-7.12 (m, 5H), 6.47 (d, *J* = 8.4 Hz, 1H), 4.68 (t, *J* = 7.4 Hz, 1H), 3.30 (d, *J* = 7.4 Hz, 2H); ^{13}C NMR (75 MHz, $CDCl_3$) δ 143.7, 135.1, 132.9, 130.0, 128.3, 127.5, 125.0, 123.1, 121.9, 118.2, 117.5, 112.8, 112.6, 110.5, 36.8, 34.1.

3,3'-(2-(4-fluorophenyl)ethane-1,1-diyl)bis(5-bromo-1*H*-indole) (3d)

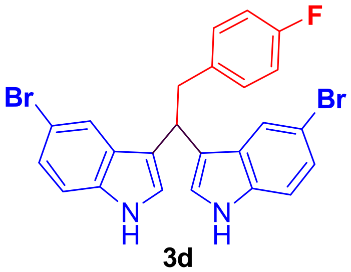

3d

Light yellow viscous liquid;[3] Yield: 415 mg (81%); ^{1}H NMR (300 MHz, $CDCl_3$) δ 7.97 (s, 2H), 7.46 (d, *J* = 0.8 Hz, 2H), 7.08-7.29 (m, 5H), 6.93-6.97 (m, 3H), 6.45 (d, *J* = 8.4 Hz, 1H), 4.66 (t, *J* = 7.4 Hz, 1H), 3.26 (d, *J* = 7.4 Hz, 2H); ^{13}C NMR (75 MHz, $CDCl_3$) δ 143.8, 135.2, 132.9, 130.0, 128.4, 127.5, 124.9, 123.0, 122.0, 118.4, 117.4, 112.7, 112.6, 110.5, 36.9, 34.2.

3,3'-(2-(4-fluorophenyl)ethane-1,1-diyl)bis(5-methyl-1*H*-indole) (3e)

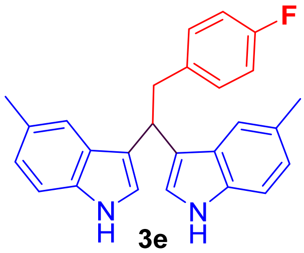

3e

Off-white semi-solid;[2] Yield: 290 mg (76%); ^{1}H NMR (300 MHz, $CDCl_3$) δ 7.79 (s, 2H), 7.21 -7.28 (m, 5H), 6.80-7.00 (m, 6H), 6.49 (s, 1H), 6.48 (s, 1H), 4.81 (t, *J* = 7.2 Hz, 1H), 3.38 (d, *J* = 7.2 Hz, 2H), 2.38 (s, 6H); ^{13}C NMR (75 MHz, $CDCl_3$) δ 142.3, 134.9, 130.9, 128.2, 127.9, 127.3, 127.2, 126.4, 123.4, 122.1, 119.4, 115.9, 110.6, 37.3, 34.6, 21.5.

3,3'-(2-phenylethane-1,1-diyl)bis(1-methyl-1*H*-indole) (3f)

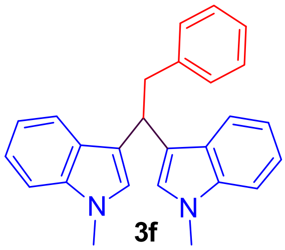

3f

Light yellow solid;[2] m.p.: 143-145 °C; Yield: 298 mg (82%); ^{1}H NMR (300 MHz, $CDCl_3$) δ 7.52-7.56 (m, 2H), 7.31 -7.33 (m, 3H), 7.21-7.26 (m, 2H), 7.12-7.18 (m, 1H), 7.02-7.09 (m,

3H), 6.91 (s, 2H), 6.62-6.67 (m, 1H), 6.51-6.54(m, 1H), 4.86 (t, $J = 7.2$ Hz, 1H), 3.74 (s, 6H), 3.40 (d, $J = 7.2$ Hz, 2H); ^{13}C NMR (75 MHz, $CDCl_3$) δ 147.8, 137.3, 129.8, 127.3, 126.6, 125.7, 121.5, 119.8, 118.6, 118.4, 117.0, 109.7, 109.1, 37.6, 34.4, 32.7.

3,3'-(2-(4-fluorophenyl)ethane-1,1-diyl)bis(1-methyl-1*H*-indole) (3g)

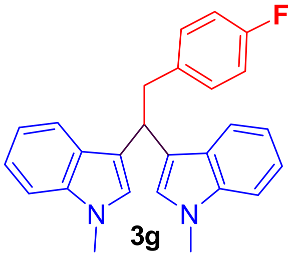

Light yellow solid;[2] m.p.: 143-145 ºC; Yield: 328 mg (86%); ^{1}H NMR (300 MHz, $CDCl_3$) δ 7.53-7.57 (m, 2H), 7.31 -7.34 (m, 2H), 7.21-7.26 (m, 2H), 7.12-7.18 (m, 1H), 7.02-7.09 (m, 3H), 6.91 (s, 2H), 6.63-6.68 (m, 1H), 6.52-6.545 (m, 1H), 4.87 (t, $J = 7.2$ Hz, 1H), 3.74 (s, 6H), 3.41 (d, $J = 7.2$ Hz, 2H; ^{13}C NMR (75 MHz, $CDCl_3$) δ 147.7, 137.4, 129.8, 127.3, 127.1, 126.6, 125.7, 121.5, 119.8, 118.7, 118.4, 117.0, 109.7, 109.1, 37.6, 34.4, 32.7.

3,3'-(2-phenylethane-1,1-diyl)bis(5-fluoro-1*H*-indole) (3h)

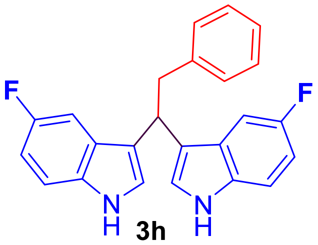

Light brown viscous liquid.;[3] Yield: 280 mg (75%); ^{1}H NMR (300 MHz, $CDCl_3$) δ 7.99 (s, 2H), 7.21-7.28 (m, 2H), 7.00-7.08 (m, 4H), 6.86-6.93 (m, 2H), 6.50-6.72 (m, 3H), 4.69 (t, $J =$ 7.5 Hz, 1H), 3.36 (d, $J = 7.5$ Hz, 2H); ^{13}C NMR (75 MHz, $CDCl_3$) δ 166.6, 160.7, 143.3, 142.3, 141.2, 140.3, 129.4, 129.3, 126.3, 126.1, 125.5, 121.6, 118.5, 117.7, 115.2, 114.9, 111.7, 43.1, 29.5.

3,3'-(2-phenylethane-1,1-diyl)bis(5-methoxy-1*H*-indole) (3i)

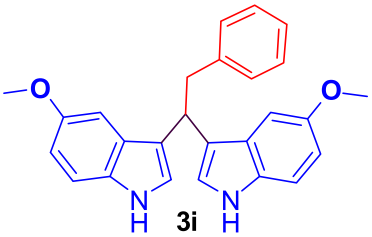

Light brown viscous liquid;[3] Yield: 284 mg (69%); ^{1}H NMR (300 MHz, $CDCl_3$) δ 7.88 (s, 2H), 7.29 (s 1H), 7.20 (d, *J* = 8.7Hz, 2H), 6.98 (d, *J* = 2.3 Hz, 2H), 6.88 (d, *J* = 2.4 Hz, 2H), 6.81 (dd, *J* = 8.8, 2.4 Hz, 2H), 6.51-6.60 (m, 4H), 4.73 (t, *J* = 7.3 Hz,1H), 3.72 (s, 6H), 3.40 (d, *J* = 7.3 Hz, 2H); ^{13}C NMR (75 MHz, $CDCl_3$) δ 153.6, 152.9, 138.4, 131.7, 127.9, 127.4, 122.6, 119.3, 117.0, 116.2, 112.6, 111.9, 111.7, 101.7, 55.8, 37.5, 34.8.

3,3'-(2-(4-fluorophenyl)ethane-1,1-diyl)bis(5-methoxy-1*H*-indole) (3j)

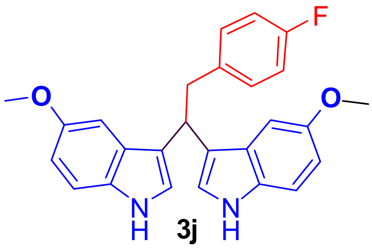

Light viscous liquid;[1] Yield: 323 mg (75%); ^{1}H NMR (300 MHz, DMSO-d_6) δ 10.56 (s, 2H), 7.25 (d, *J* = 2.3 Hz, 2H), 7.18 (d, *J* = 8.7 Hz, 2H), 7.03 (d, *J* = 2.3 Hz, 2H), 6.81 (dd, *J* = 6.3, 2.4 Hz, 2H), 6.42-6.45 (m, 4H), 4.76 (t, *J* = 7.5 Hz, 1H), 3.72 (s, 6H), 3.33 (d, *J* = 7.5 Hz, 2H); ^{13}C NMR (75 MHz, DMSO-d_6) δ 152.9, 151.2, 140.2, 132.1, 127.5, 127.1, 123.5, 118.9, 116.2, 112.2, 110.8, 101.8, 55.8, 36.5, 32.9.

3,3'-(2-phenylethane-1,1-diyl)bis(1-ethyl-1*H*-indole) (3k)

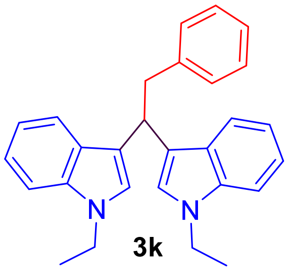

Light brown viscous liquid;[3] Yield: 322 mg (82%); ^{1}H NMR (300 MHz, $CDCl_3$) δ 7.3 (d, *J* = 7.9 Hz, 2H), 7.34 (d, *J* = 7.9 Hz, 2H), 7.28 (s, 1H), 7.17-7.23 (m, 2H), 7.09-7.14 (m, 2H), 6.99-7.04 (m, 2H), 6.96 (s, 2H), 6.66 (td, *J* = 7.9, 1.1 Hz, 1H), 6.51 (d, *J* = 7.9 Hz, 1H), 4.81 (t, *J* = 7.1 Hz, 1H), 4.13 (q, *J* = 7.2 Hz, 4H). 3.43 (d, *J* = 7.2 Hz, 2H), 1.43 (t, *J* = 7.2 Hz, 6H); ^{13}C NMR (75 MHz, $CDCl_3$) δ 147.1, 136.4, 130.3, 127.5, 127.1, 126.1, 125.1, 121.3, 120.1, 118.6, 118.2, 116.9, 110.3, 109.3, 40.9, 37.5, 35.6, 15.6.

3,3'-(2-phenylethane-1,1-diyl)bis(1-benzyl-1*H*-indole) (3l)

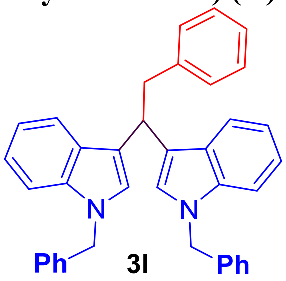

3l

Brown viscous liquid;[3] Yield: 407 mg (79%); ^{1}H NMR (300 MHz, $CDCl_3$) δ 7.55 (d, *J* = 7.9 Hz, 2H), 7.25-7.36 (m, 11H), 7.14-7.21 (m, 1H), 6.99-7.11 (m, 9H), 6.68 (t, *J* = 7.3 Hz, 1H), 6.56 (d, *J* = 7.9 Hz, 1H), 5.28 (s, 4H), 4.96 (t, *J* = 7.3 Hz, 1H), 3.54 (d, *J* = 7.3 Hz, 2H); ^{13}C NMR (75 MHz, $CDCl_3$) δ 146.4, 139.7, 137.9, 137.0, 130.4, 128.8, 128.5, 127.6, 127.5, 127.4, 127.0, 126.6, 126.2, 121.7, 120.0, 119.0, 118.7, 117.3, 110.8, 109.7, 49.8, 48.2, 35.0.

3,3'-(2-(*p*-tolyl)ethane-1,1-diyl)bis(1-methyl-1*H*-indole) (3m)

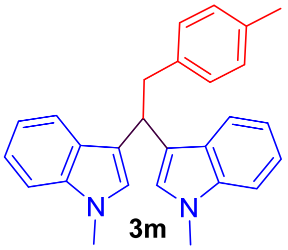

3m

Light yellow solid;[2] m.p.: 137-139 °C; Yield: 295 mg (78%); ^{1}H NMR (300 MHz, $CDCl_3$) δ 7.52 (d, *J* = 7.9 Hz, 2H), 7.28 -7.32 (m, 2H), 7.00-7.25 (m, 6H), 6.91 (s, 2H), 6.70 (dd, *J* = 7.4, 0.8 Hz, 1H), 6.58 (d, *J* = 7.9 Hz, 1H), 4.86 (t, *J* = 7.2 Hz, 1H), 3.73 (s, 6H), 3.41 (d, *J* = 7.2 Hz, 2H), 2.45 (s, 3H); ^{13}C NMR (75 MHz, $CDCl_3$) δ 137.3, 129.9, 127.2, 127.1, 126.7, 126.6, 126.4, 121.5, 119.7, 118.6, 118.3, 110.6, 109.1, 37.5, 34.4, 32.7, 31.1.

4. General procedure for Markovnikov hydroindolation

To a stirred solution of various indoles (2 mmol) and terminal alkynes (1.5 mmol) in water/methanol (1/1, 2 mL) as a solvent, *p*-toluenesulfonic acid monohydrate (70 mg, 20 mol%) was added, and the reaction temperature was raised to 100 °C for 24 h. After the reaction was completed, it was cooled, extracted with DCM and water, and then washed with brain. The resulting organic layer was dried over anhydrous Na_2SO_4, and the organic layer was concentrated under reduced pressure. After obtaining a crude product, it was purified using 10% of DCM-hexane eluent on silica gel column to give a pure desired product.

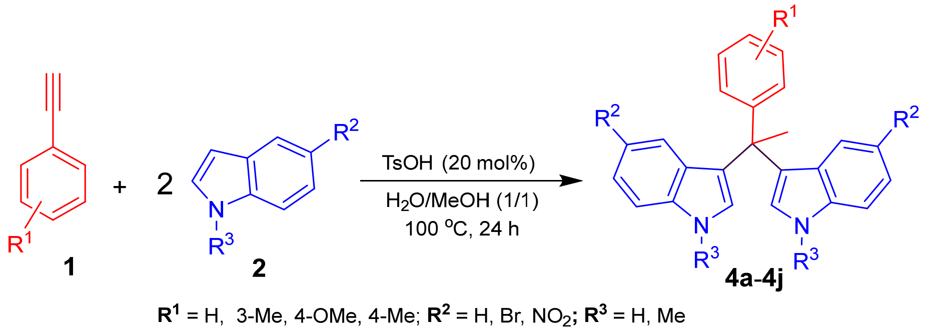

Scheme S2. Synthesis of Markovnikov additions of various indoles and terminal aryl alkynes

4.1. NMR spectral data of compounds for Markovnikov hydroindolation

3,3'-(1-(4-methoxyphenyl)ethane-1,1-diyl)bis(*1H*-indole) (4a)

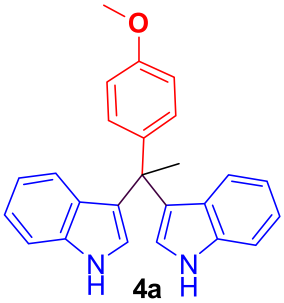

4a

White solid;[4] m.p.: 119–120 °C; Yield: 337 mg (92%); ^{1}H NMR (300 MHz, $CDCl_3$) δ 7.85 (s, 2H), 7.32 -7.38 (m, 7H), 7.14-7.17 (m, 2H), 6.97-6.99 (m, 2H), 6.83 (d, *J* = 8.9 Hz, 2H), 6.63 (d, *J* = 2.8 Hz, 2H), 3.81 (s, 3H), 2.37 (s, 3H); ^{13}C NMR (75 MHz, $CDCl_3$) δ 157.5, 140.4, 137.1, 129.1, 126.5, 124.9, 123.4, 122.2, 121.5, 118.9, 113.1, 111.2, 55.2, 43.1, 28.9.

3,3'-(1-(4-methoxyphenyl)ethane-1,1-diyl)bis(1-methyl-1*H*-indole) (4b)

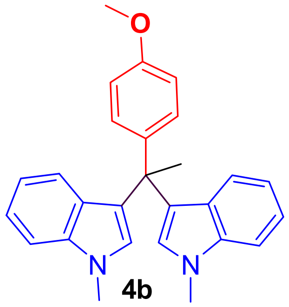

4b

Yellow solid.;[5] m.p.: 200-203 °C; Yield: 370 mg (94%); ^{1}H NMR (300 MHz, $CDCl_3$) δ 7.32 - 7.40 (m, 6H), 7.20-7.25 (m, 2H), 6.97-7.02 (m, 2H), 6.84 (d, *J* = 9.0 Hz, 2H), 6.53 (s, 2H), 3.83 (s, 3H), 3.71(s, 6H), 2.39 (s, 3H); ^{13}C NMR (75 MHz, $CDCl_3$) δ 157.5, 140.8, 137.8, 129.1, 128.1, 126.9, 123.7, 122.3, 121.1, 118.4, 113.0, 109.2, 55.2, 43.1, 32.7, 29.3.

3,3'-(1-(*p*-tolyl)ethane-1,1-diyl)bis(1-methyl-1*H*-indole) (4c)

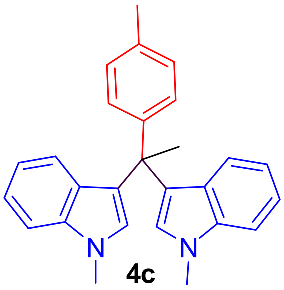

4c

Yellow solid;[5] m.p.: 199-201 ºC; Yield: 318 mg (84%); ^{1}H NMR (300 MHz, $CDCl_3$) δ 7.34 - 7.41 (m, 6H), 7.21-7.30 (m, 2H), 7.12 (d, J = 8.0, 2H), 6.97-7.02 (m, 2H), 6.56 (s, 2H), 3.72 (s, 6H), 2.40 (s, 3H), 2.39 (s, 3H); ^{13}C NMR (75 MHz, $CDCl_3$) δ 145.4, 137.8, 135.1, 128.5, 128.1, 127.9, 126.9, 123.5, 122.3, 121.0, 118.4, 109.2, 43.4, 32.7, 29.2, 21.1.

3,3'-(1-phenylethane-1,1-diyl)bis(5-nitro-1*H*-indole) (4d)

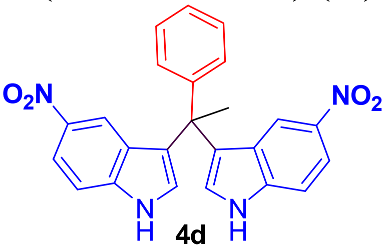

4d

Yellow solid;[7] m.p.: 198−201 °C; Yield: 392 mg (92%); ^{1}H NMR (300 MHz, $CDCl_3$) δ 8.60 (s, 2H), 8.17 (d, J = 2.2 Hz, 2H), 8.05 (dd, J = 6.8, 2.2 Hz, 2H), 7.29-7.44 (m, 8H), 6.98 (d, J = 2.4 Hz, 2H), 2.42 (s, 3H); ^{13}C NMR (75 MHz, $CDCl_3$) δ 146.4, 141.2, 140.2, 128.4, 127.7, 126.8, 126.33, 126.30, 125.7, 118.6, 117.6, 111.6, 43.5, 29.4.

3,3'-(1-(4-methoxyphenyl)ethane-1,1-diyl)bis(5-bromo-1*H*-indole) (4e)

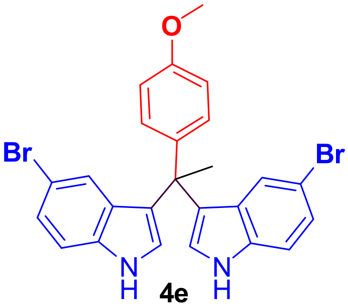

4e

White solid;[8] m.p.: 112–114 °C; Yield: 476 mg (91%); ^{1}H NMR (300 MHz, acetone-d_6) δ 10.83 (s, 1H), 10.68 (s, 1H), 8.20 (d, J = 1.8 Hz, 1H), 7.94-8.03 (m, 3H), 7.76 (d, J = 1.8 Hz, 1H), 7.52 (d, J = 8.6 Hz, 1H), 7.35-7.38 (m, 2H), 7.19 (dd, J = 6.6, 1.9 Hz, 1H), 7.02 (d, J = 9.0 Hz, 2H), 6.86 (d, J = 1.5 Hz, 1H), 3.89 (s, 3H), 2.50 (s, 3H); ^{13}C NMR (75 MHz, acetone-d_6) δ 158.0, 139.8, 136.3, 128.9, 128.3, 125.1, 123.7, 123.53, 123.47, 113.4, 113.0, 111.3, 54.5, 42.7, 29.8 (merged with acetone-d_6 peak).

3,3'-(1-(m-tolyl)ethane-1,1-diyl)bis(1*H*-indole) (4f)

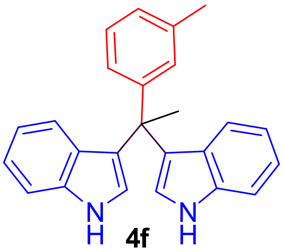

4f

White solid;[4] m.p.: 190−191 °C: Yield: 315 mg (90%); ^{1}H NMR (300 MHz, $CDCl_3$) δ 7.81 (s, 2H), 7.35 -7.39 (m, 4H), 7.29 (s, 1H), 7.15-7.19 (m, 4H), 7.04-7.06 (m, 1H), 6.95-6.98 (m, 2H), 6.63 (d, J = 2.5 Hz, 2H), 2.39 (s, 3H), 2.30(s, 3H); ^{13}C NMR (75 MHz, $CDCl_3$) δ 147.9, 137.2, 137.1, 128.7, 127.7, 126.7, 126.5, 125.4, 124.8, 123.5, 122.2, 121.5, 118.9, 111.2, 43.7, 28.8, 21.8.

3,3'-(1-phenylethane-1,1-diyl)bis(1*H*-indole) (4g)

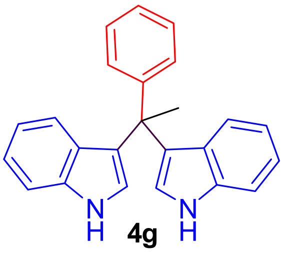

4g

White solid;[4] m.p.: 191−193 °C; Yield: 225 mg (67%); ^{1}H NMR (300 MHz, $CDCl_3$) δ 7.83 (s, 2H), 7.42 -7.45 (m, 2H), 7.35-7.38(m, 4H), 7.23-7.32 (m, 3H), 7.15-7.20 (m, 2H), 6.95-7.00 (m, 2H), 6.63 (d, J = 2.5 Hz, 2H), 2.40 (s, 3H); ^{13}C NMR (75 MHz, $CDCl_3$) δ 148.1, 137.1, 128.1, 127.9, 126.5, 125.9, 124.7, 123.5, 122.1, 121.6, 118.9, 111.3, 43.8, 28.8.

3,3'-(1-phenylethane-1,1-diyl)bis(1-methyl-1*H*-indole) (4h)

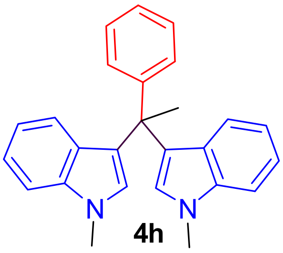

4h

White solid;[4] m.p.: 196−197 °C; Yield: 288 mg (79%); ^{1}H NMR (300 MHz, $CDCl_3$) δ 7.45 (d, J = 7.0 Hz, 2H), 7.19-7.37 (m, 10H), 6.97 (t, J = 7.7 Hz. 2H), 6.53 (s, 2H), 2.40 (s, 3H); ^{13}C NMR (75 MHz, $CDCl_3$) δ 148.4, 137.8, 128.1, 127.8, 126.9, 125.7, 123.3, 122.2, 121.0, 118.4, 109.2, 43.7, 32.7, 29.2.

3,3'-(1-phenylethane-1,1-diyl)bis(5-bromo-1*H*-indole) (4i)

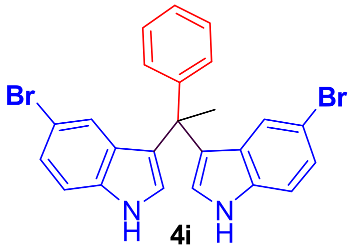

4i

White solid;[4] m.p.: 184−186 °C; Yield: 418 mg (84%); ^{1}H NMR (300 MHz, DMSO-d_6) δ 11.12 (s, 2H), 7.35 (dd, *J* = 7.9, 0.5 Hz, 2H), 7.19-7.29 (m, 5H), 7.13 (d *J* = 1.9 Hz, 1H), 7.10 (s, 3H), 6.86 (d, *J* = 2.5 Hz, 2H), 2.19 (s, 3H); ^{13}C NMR (75 MHz, DMSO-d_6) δ 147.9, 136.2, 128.3, 128.3, 128.0, 126.4, 125.6, 123.6, 123.2, 122.6, 114.2, 111.2, 43.4, 29.7.

3,3'-(1-(*p*-tolyl)ethane-1,1-diyl)bis(5-bromo-1*H*-indole) (4j)

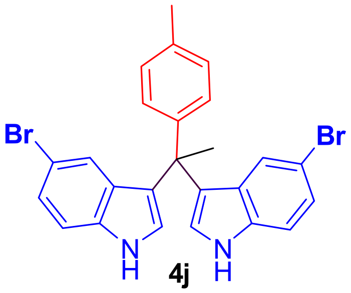

4j

Off-White solid;[6] m.p.: 194−197 °C; Yield: 453 mg (89%); ^{1}H NMR (300 MHz, $CDCl_3$) δ 7.95 (s, 2H), 7.44 (d, *J* = 0.6 Hz, 2H), 7.22-7.29 (m, 6H), 7.10 (d, *J* = 8.0 Hz, 2H), 6.66 (d, *J* = 2.5 Hz, 2H), 2.36 (s, 3H), 2.31 (s, 3H); ^{13}C NMR (75 MHz, $CDCl_3$) δ 144.1, 135.73, 135.65, 128.8, 128.1, 127.7, 124.6, 124.5, 124.2, 112.7, 112.4, 43.2, 28.8, 21.0.

5. General procedure for gram-scale synthesis

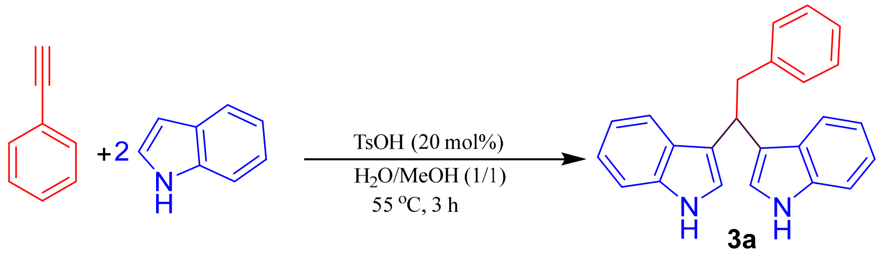

Scheme S3. Gram-scale synthesis of anti-Markovnikov hydroindolation

To a stirred solution of indole (1.172 g, 10 mmol) and phenylacetylene (1.15 g, 7.5 mmol) in water/methanol as a solvent, *p*-toluenesulfonic acid monohydrate (350 mg, 20 mol%) was added, and the reaction temperature was raised to 55 °C for 3 h. After the reaction was

completed, it was cooled, extracted with DCM and water, and then washed with brain. The resulting organic layer was dried over anhydrous Na_2SO_4, and the solution was concentrated under reduced pressure. After obtaining a crude product, it was purified using 20% of DCM-hexane eluent on silica gel column to give a pure **3a** (2.83 g, 82%).

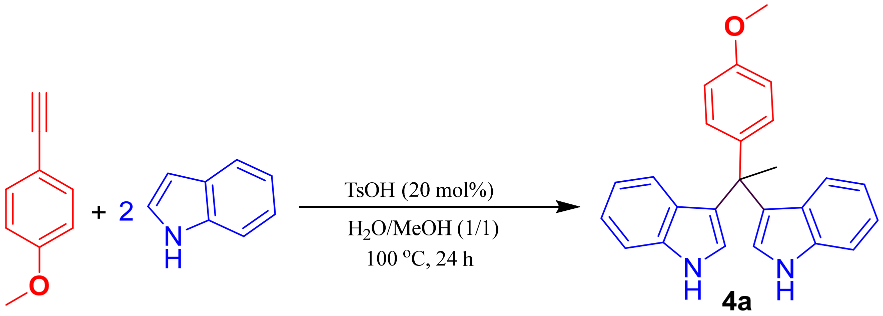

Scheme S4. Gram-scale synthesis of Markovnikov hydroindolation

To a stirred solution of indole (0.70 g, 6 mmol) and 4-methoxyphenylacetylene (0.595 g, 4.5 mmol) in water/methanol as a solvent, *p*-toluenesulfonic acid monohydrate (210 mg, 20 mol%) was added, and the reaction temperature was raised to 100 °C for 24 h. After the reaction was completed, it was cooled, extracted with DCM and water, and then washed with brain. The resulting organic layer was dried over anhydrous Na_2SO_4, and the solution was concentrated under reduced pressure. After obtaining a crude product, it was purified using 10% of DCM-hexane eluent on silica gel column to give a pure **4a** (0.95 g, 84%).

6. Control experiments for mechanistic studies

6.1. General procedure for anti-Markovnikov addition *via* phenylacetaldehyde

To a stirred solution of indole (234 mg, 2 mmol) and phenylacetaldehyde (181 mg, 1.5 mmol) in a water/methanol solvent mixture, *p*-toluenesulfonic acid monohydrate (70 mg, 20 mol%) was added. The reaction mixture was heated to 55 °C and maintained at this temperature for 3 h. After completion of the reaction, the mixture was cooled and extracted with DCM and water. The organic layer was then washed with brine, dried over anhydrous Na_2SO_4, and concentrated under reduced pressure. The resulting crude semisolid was purified using silica gel column chromatography with a 20% DCM-hexane eluent. A pure **3a** molecule (75%) was obtained as an off-white semi-solid.

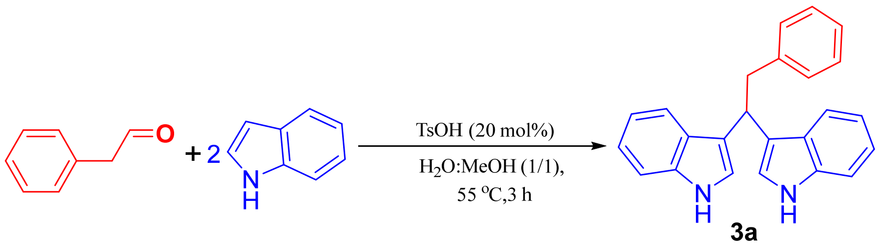

Scheme S5. Synthesis of anti-Markovnikov addition derivative from indole and phenylacetaldehyde

6.2. General procedure for anti-Markovnikov addition *via* (*E*)-3-styryl-1*H*-indole

To a stirred solution of indole (88 mg, 0.75 mmol) and (*E*)-3-styryl-1*H*-indole (**I-1**)[3] (110 mg, 0.5 mmol) in a water/methanol solvent mixture, *p*-toluenesulfonic acid monohydrate (70 mg, 20 mol%) was added. The reaction mixture was heated to 60 °C and maintained at this temperature for 24 h. After completion of the reaction, the reaction mixture was cooled and extracted with DCM and water. The organic layer was washed with brine, dried over anhydrous Na_2SO_4, and concentrated under reduced pressure. The resulting crude semisolid was purified through silica gel column chromatography using a 20% DCM-hexane eluent. A pure **3a** molecule was obtained as a off-white semi-solid.

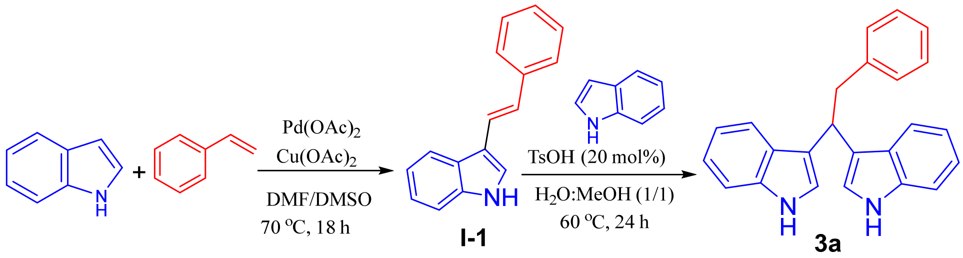

Scheme S6. Synthesis of anti-Markovnikov addition derivative from indole and (*E*)-3-styryl-1*H*-indole

6.3. General procedure for 3-(1-phenyl-vinyl)-1*H*-indole intermediate (N)

The compound **N** was synthesized using a reported procedure.[9] 3-Acetylindole (10 mmol, 1.00 equiv.) was stirred at 0 °C in freshly distilled, dry THF under a nitrogen atmosphere. Then, a 1.0 M solution of phenylmagnesium bromide in THF (25 mmol, 2.5 equiv.) was added dropwise to the reaction mixture. After the addition was completed, the reaction mixture was heated from 0 °C to 50 °C and stirred for 12 h. Upon completion of the reaction, the mixture was cooled to 0 °C, and saturated ammonium chloride solution was added. The reaction mixture was then extracted with diethyl ether (150 mL × 3), washed with brine, and the combined organic layers were dried over Na_2SO_4. The organic layer was filtered and

concentrated using rotary evaporation to yield a crude semi-solid intermediate product. The crude intermediate was dissolved in DCM (50 mL), and anhydrous $MgSO_4$ (5 g, 0.5 g per mmol of substrate) and 200–300 mesh silica gel (5 g) were added. The reaction mixture was stirred for 7 hours and then filtered. The filtrate was concentrated and a crude product was purified using silica gel column chromatography with 20% DCM–hexane as the eluent to give the brownish solid vinyl product **N**.

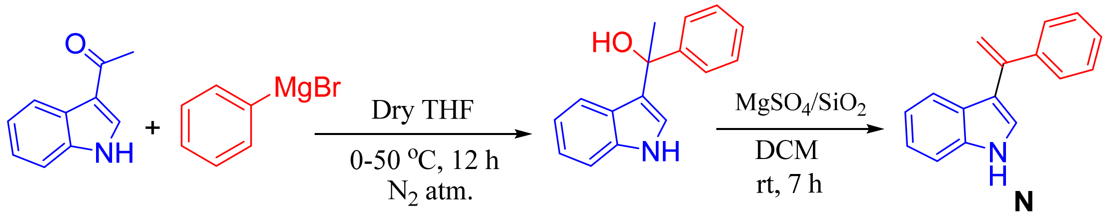

Scheme S7. Synthesis of 3-(1-phenyl-vinyl)-1*H*-indole derivative

6.4. General procedure for Markovnikov addition *via* 3-(1-phenyl-vinyl)-1*H*-indole

To a stirred solution of indole (88 mg, 0.75 mmol) or 1-methyl indole (98 mg, 0.75 mmol) and 3-(1-phenyl-vinyl)-1*H*-indole (**N**)[9] (110 mg mg, 0.5 mmol) in a water/methanol solvent mixture, *P*-toluenesulfonic acid monohydrate (35 mg, 20 mol%) was added. The reaction mixture was heated to 60 °C and maintained at this temperature for 12 h. After completion of the reaction, the reaction mixture was cooled and extracted with DCM and water. The organic layer was washed with brine, dried over anhydrous Na_2SO_4, and concentrated under reduced pressure. The resulting crude semisolid was purified through silica gel column chromatography using a 10% DCM-hexane eluent. A pure **4g** (78%) or **4k** (72%) molecule was obtained as a brownish semisolid, which then subjected to further structural analysis.

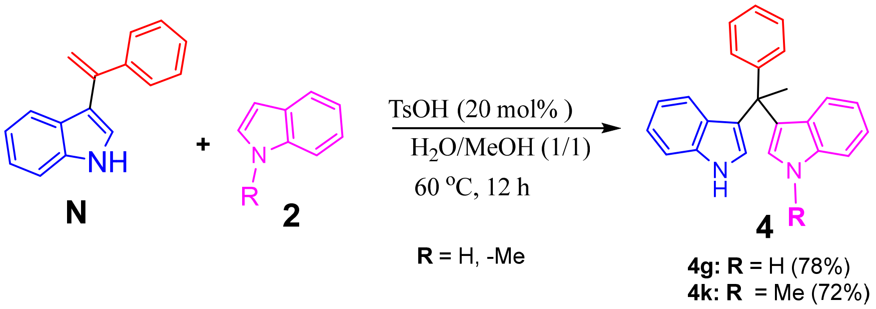

Scheme S8. Synthesis of Markovnikov addition derivatives from 3-(1-phenyl-vinyl)-1*H*-indole and various indoles

7. NMR spectral data of compounds for asymmetric Markovnikov addition

3-(1-(1*H*-indol-3-yl)-1-(*p*-tolyl)ethyl)-1-methyl-1*H*-indole (4k)

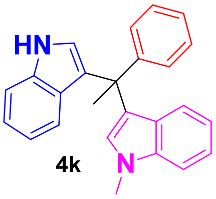

4k

White solid;[5] m.p.: 186−187 °C; Yield: 273 mg (78%); ^{1}H NMR (300 MHz, $CDCl_3$) δ 7.83 (s, 1H), 7.46 (dd, *J* = 6. 8, 1.6 Hz, 2H), 7.35-7.39 (m, 4H), 7.32 (s, 1H), 7.24-7.30 (m, 3H), 7.18-7.23 (m, 2H), 3.69 (s, 3H), 2.41 (s, 3H); ^{13}C NMR (75 MHz, $CDCl_3$) δ 148.2, 137.8, 137.1, 128.1, 127.8, 126.9, 126.5, 125.8, 124.7, 123.5, 123.3, 122.3, 122.1, 121.5, 121.1, 119.0, 118.4, 111.2, 109.3, 43.7, 32.6, 29.0.

8. References

1. Srivastava, A.; Patel, S. S.; Chandna, N.; Jain, N. Copper-Catalyzed Anti-Markovnikov Hydroindolation of Terminal Alkynes: Regioselective Synthesis of Bis(Indolyl)Alkanes. *J. Org. Chem.* **2016**, *81*, 11664–11670.
2. Guo, S.; Fang, Z.; Zhou, B.; Hua, J.; Dai, Z.; Yang, Z.; Liu, C.; He, W.; Guo, K. Cu/Pd-Catalyzed Chemoselective Synthesis of C-3 Dicarbonyl Indoles and Bis(Indolyl)Alkanes from Aldehydes and Indoles. *Org. Chem. Front.* **2019**, *6*, 627–631.
3. Tyagi, A.; Khan, J.; Yadav, N.; Mahato, R.; Hazra, C. K. Catalyst-Switchable Divergent Synthesis of Bis(indolyl)alkanes and 3-Alkylated Indoles from Styrene Oxides. *J. Org. Chem.* **2022**, *87*, 10229–10240.
4. Ling, F.; Xiao, L.; Fang, L.; Feng, C.; Xie, Z.; Lv, Y.; Zhong, W. B(C6F5) 3-Catalyzed Markovnikov Addition of Indoles to Aryl Alkynes: An Approach toward Bis(indolyl)alkanes. *Org. Biomol. Chem.* **2018**, *16*, 9274–9278.
5. Xia, D.; Wang, Y.; Du, Z.; Zheng, Q. Y.; Wang, C. Rhenium-Catalyzed Regiodivergent Addition of Indoles to Terminal Alkynes. *Org. Lett.* **2012**, *14*, 588–591.
6. Maiti, G.; Kayal, U.; Karmakar, R.; Bhattacharya, R. N. An Efficient One Pot Conversion of Alkynes to Bis(indolyl) and Bis(pyrrolyl)alkanes in Aqueous Ethanol. *Indian J. Chem. - Sect. B Org. Med. Chem.* **2013**, *52*, 122–128.
7. Yadav, J. S.; Reddy, B. V. S.; Padmavani, B.; Gupta, M. K. Gallium(III) Halide-Catalyzed Coupling of Indoles with Phenylacetylene: Synthesis of Bis(indolyl)phenylethanes. *Tetrahedron Lett*. **2004**, *45*, 7577–7579.
8. Kumar, S.; Rastogi, S. K.; Singh, A.; Bharati Ahirwar, M.; Deshmukh, M. M.; Sinha, A. K.; Kumar, R. Friedel-Crafts-Type Reaction of (Het)Arenes with Aldehydes/Ketones under Acid-Free Conditions Using Neutral Ionic Liquid: A Convenient Routes to Bis(Indolyl)Methanes and Beyond. *Asian J. Org. Chem.* **2022**, *11*, e202100749.
9. Wang, Z.; Ai, F.; Wang, Z.; Zhao, W.; Zhu, G.; Lin, Z.; Sun, J. Organocatalytic Asymmetric Synthesis of 1,1-Diarylethanes by Transfer Hydrogenation. *J. Am. Chem. Soc.* **2015**, *137*, 383–389.

9. NMR spectra

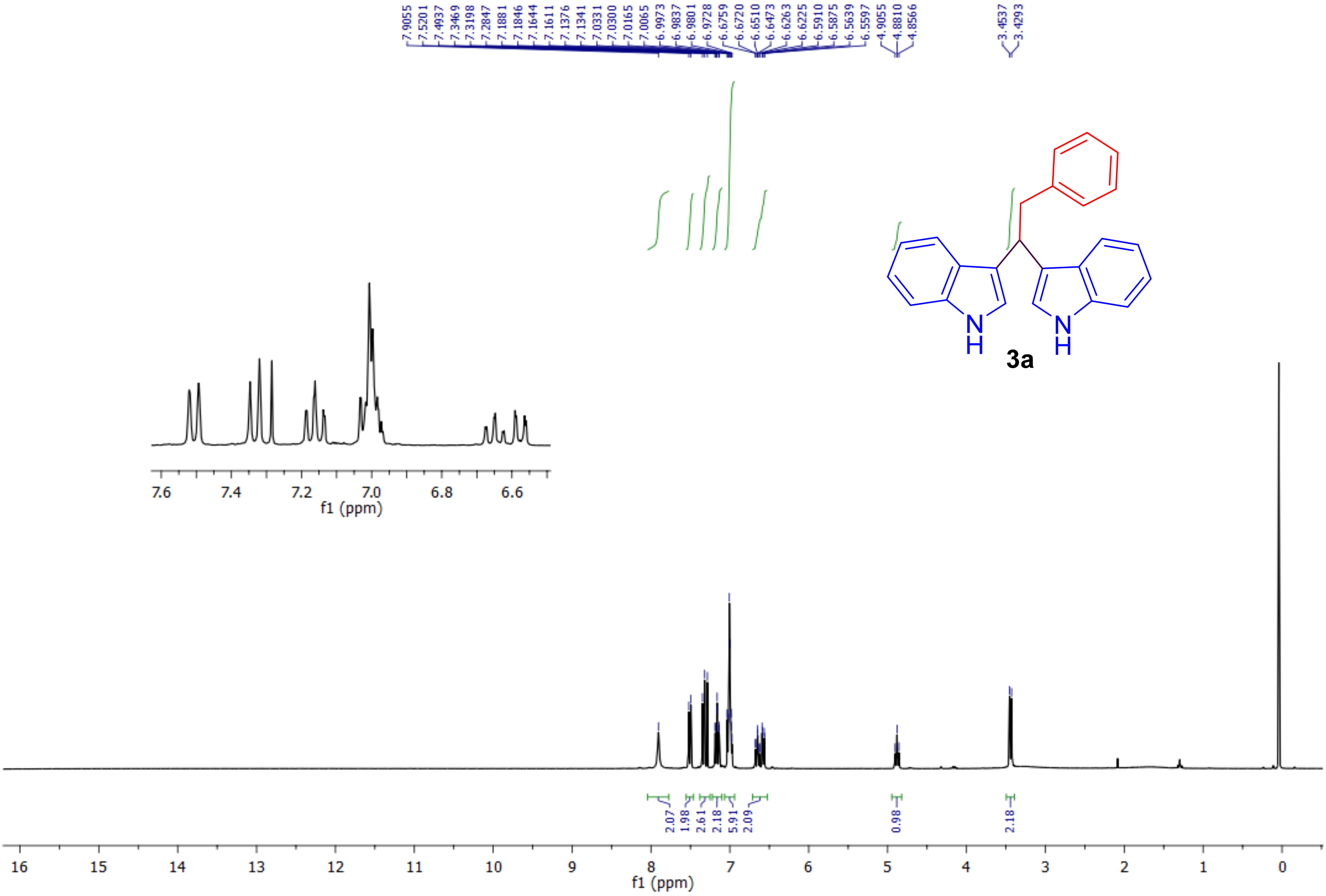

Figure S1. ^{1}H NMR Spectra of **3a** (300 MHz, $CDCl_3$)

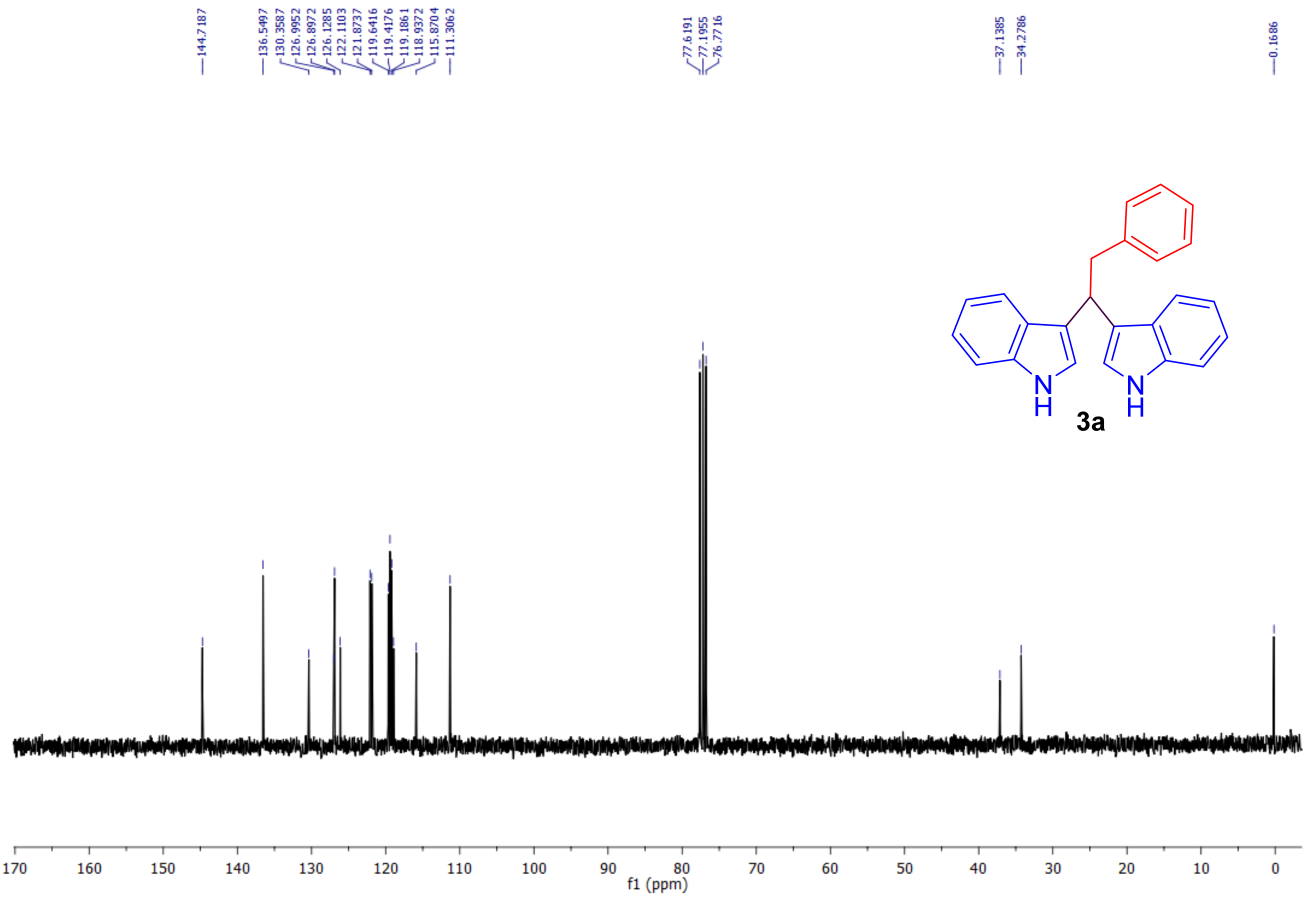

Figure S2. ^{13}C NMR Spectra of **3a** (300 MHz, $CDCl_3$)

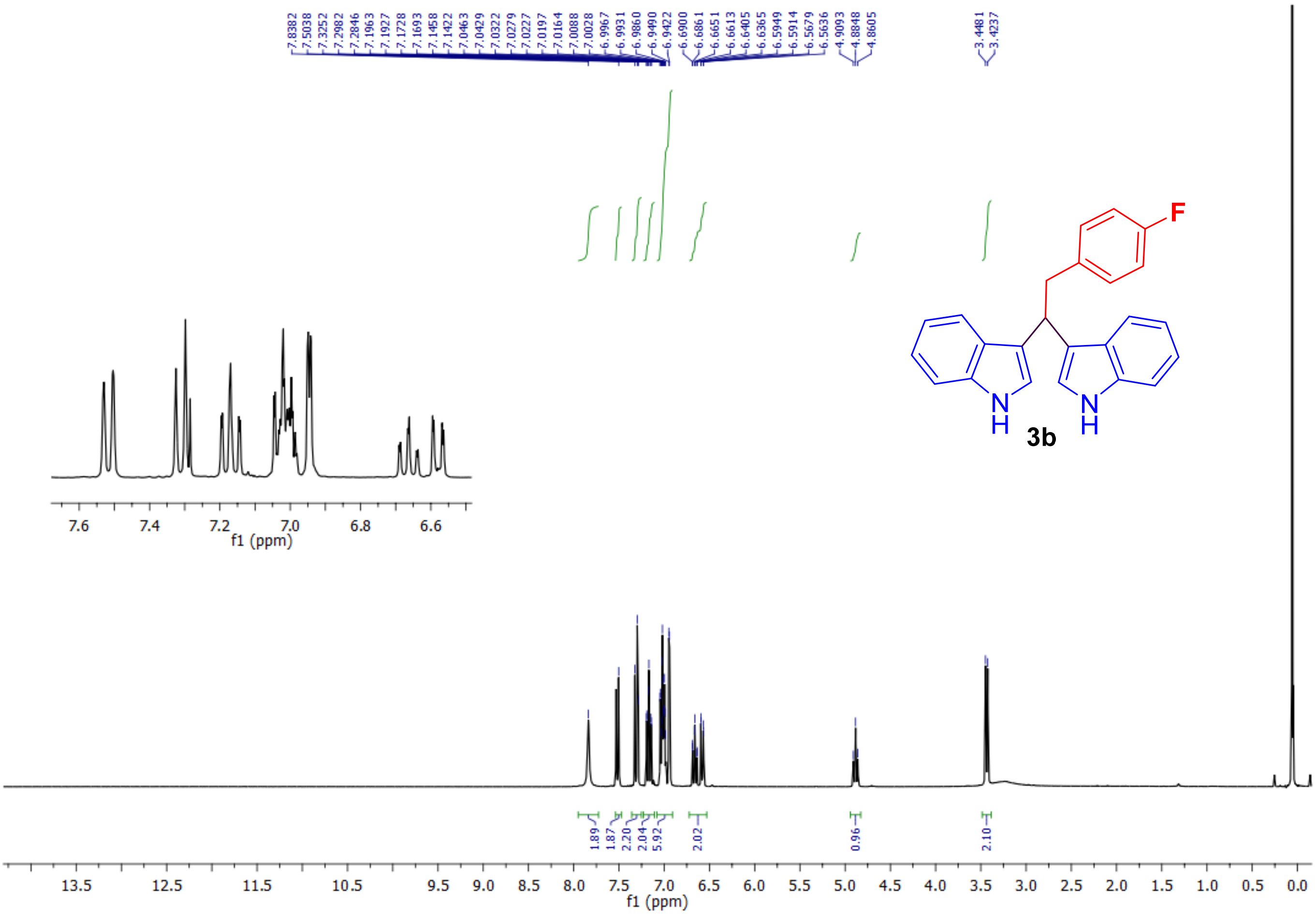

Figure S3. ^{1}H NMR Spectra of **3b** (300 MHz, $CDCl_3$)

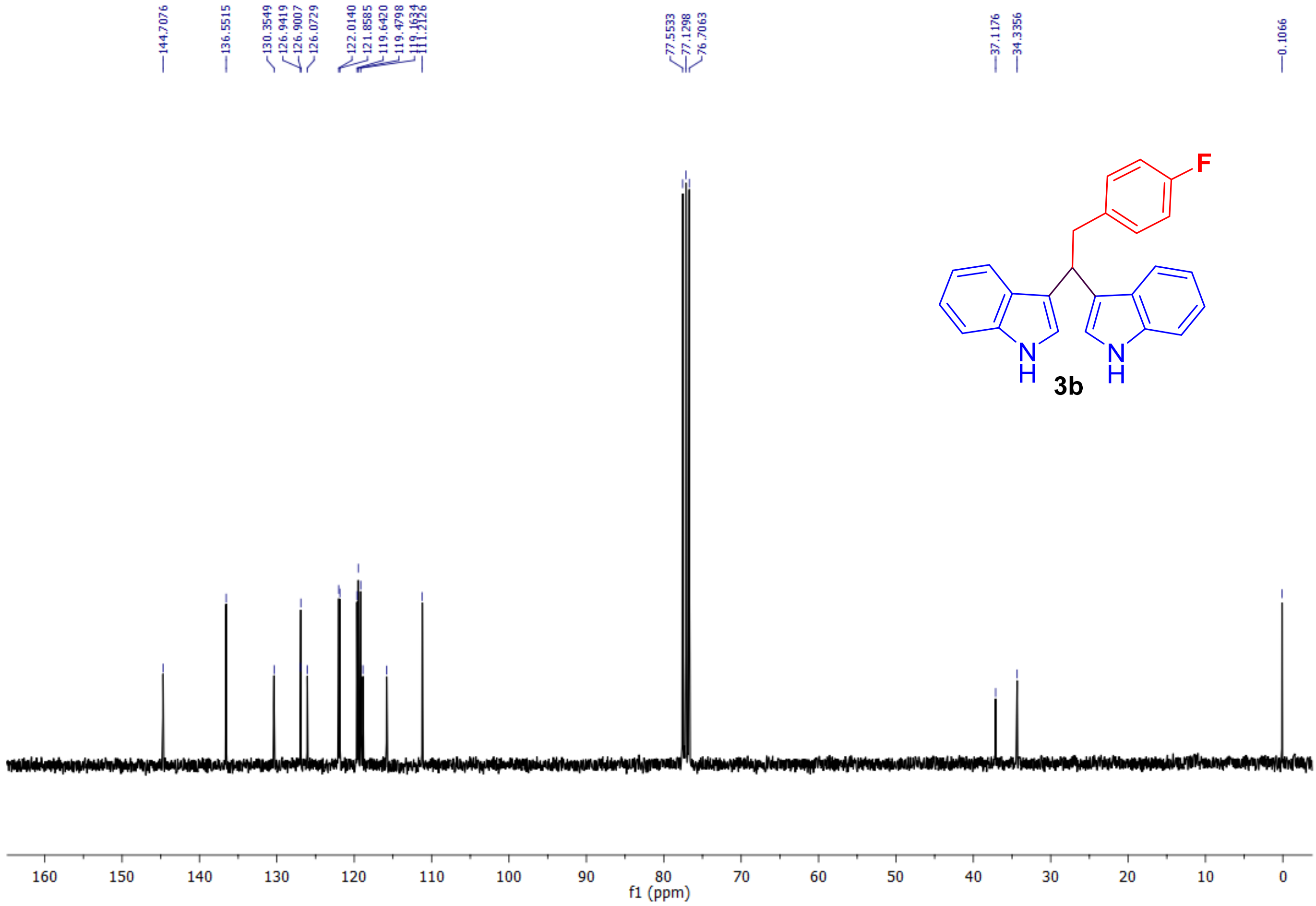

Figure S4. ^{13}C NMR Spectra of **3b** (300 MHz, $CDCl_3$)

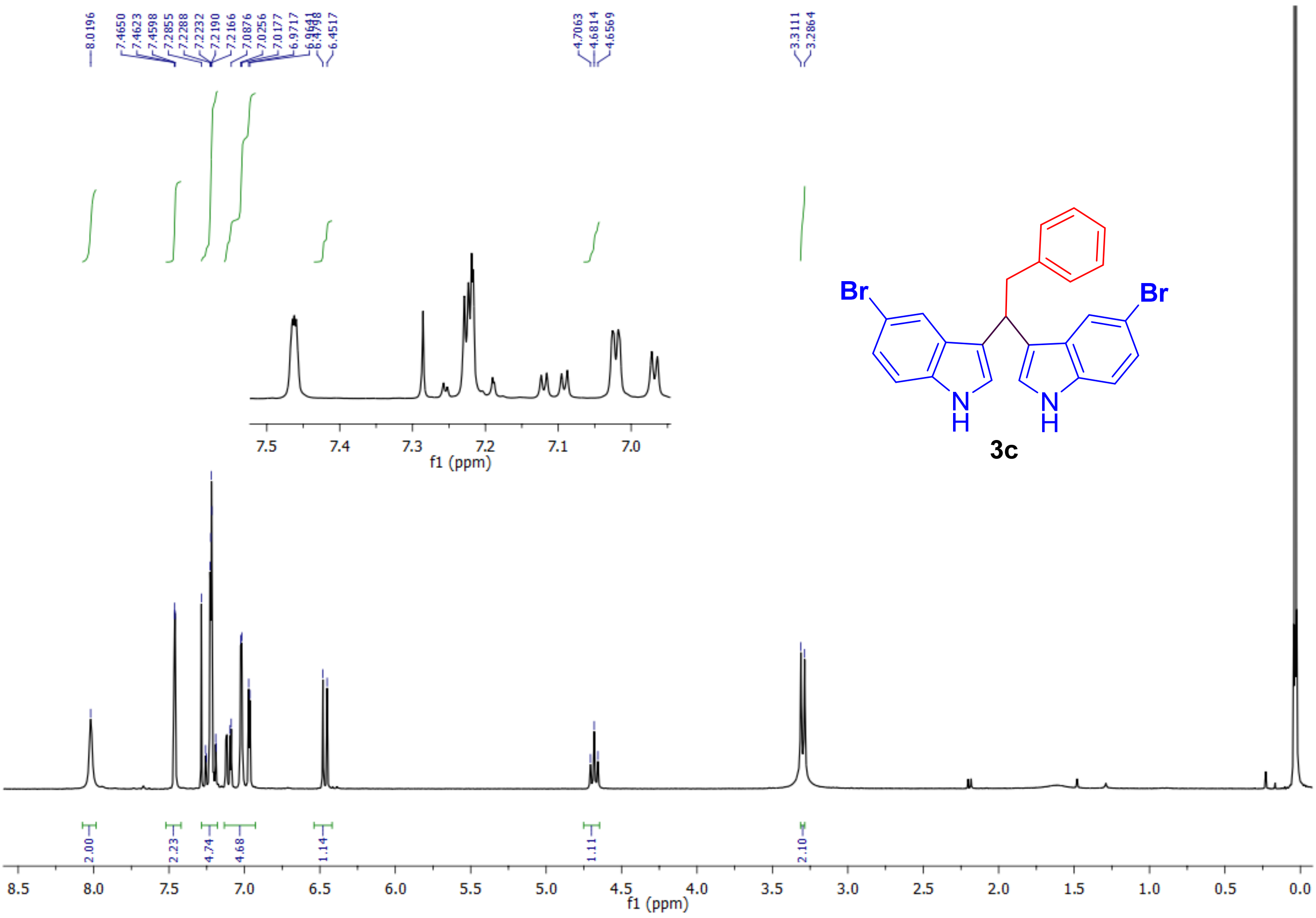

Figure S5. ^{1}H NMR Spectra of **3c** (300 MHz, $CDCl_3$)

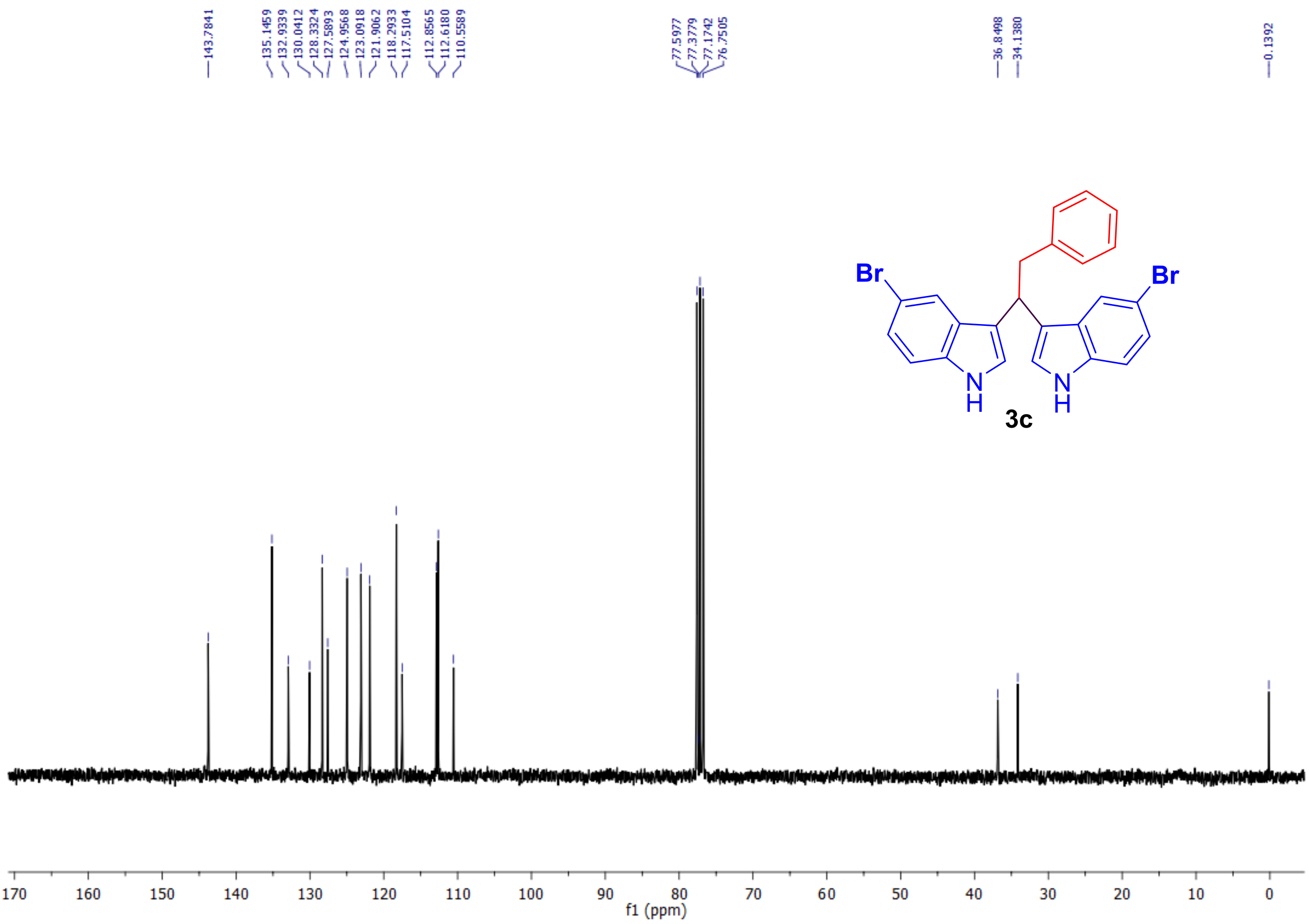

Figure S6. ^{13}C NMR Spectra of **3c** (300 MHz, $CDCl_3$)

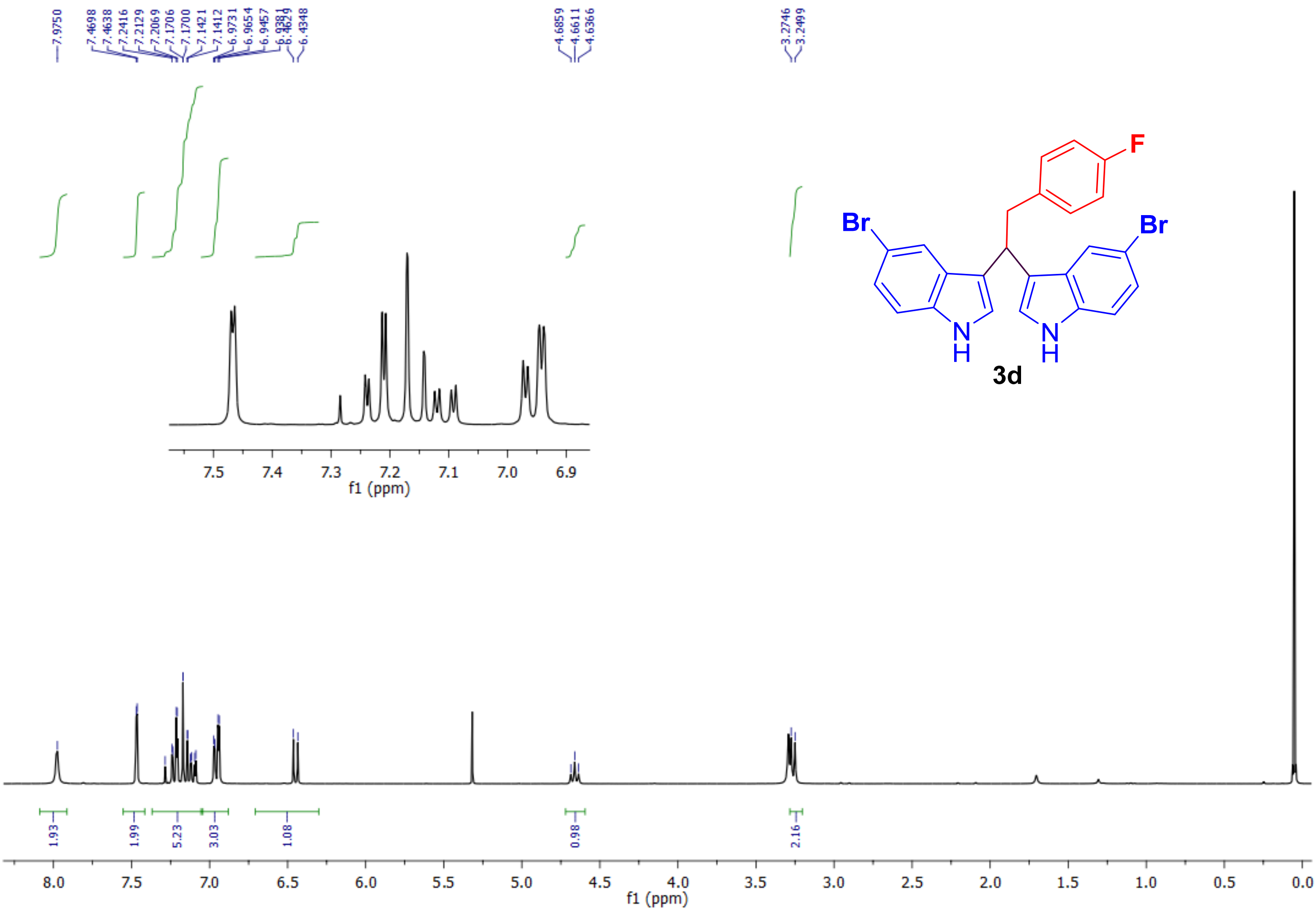

Figure S7. ^{1}H NMR Spectra of **3d** (300 MHz, $CDCl_3$)

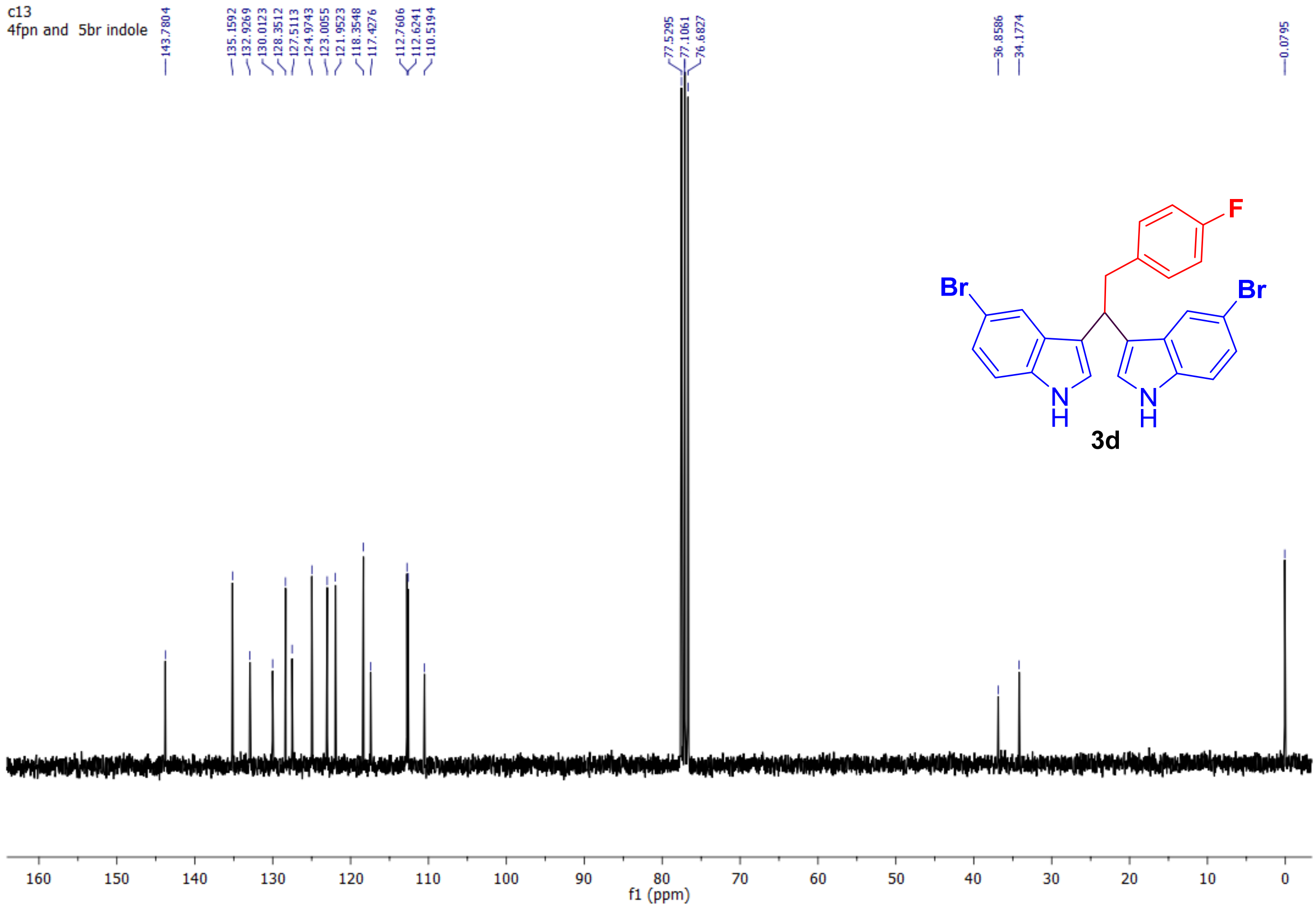

Figure S8. ^{13}C NMR Spectra of **3d** (300 MHz, $CDCl_3$)

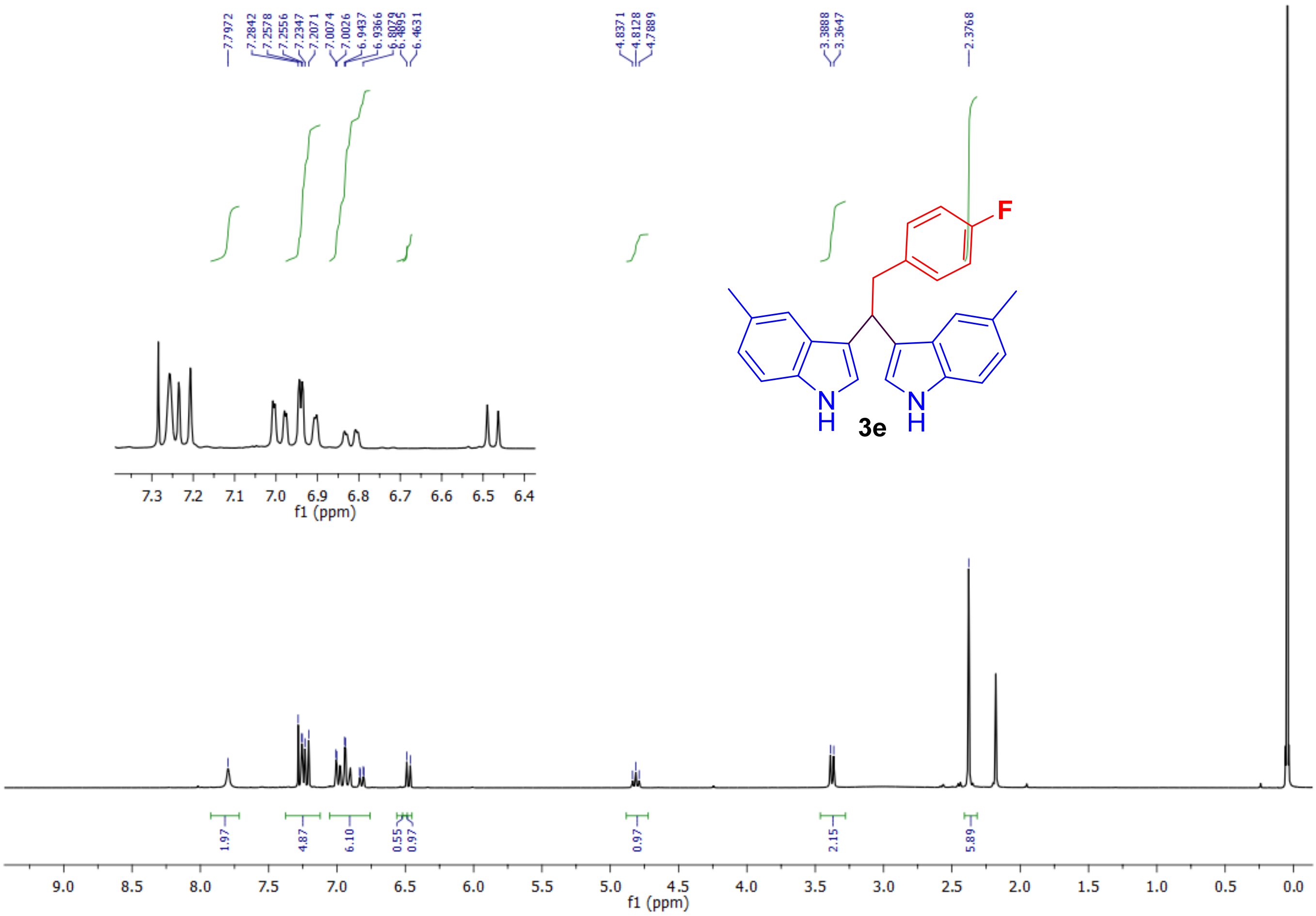

Figure S9. ^{1}H NMR Spectra of **3e** (300 MHz, $CDCl_3$)

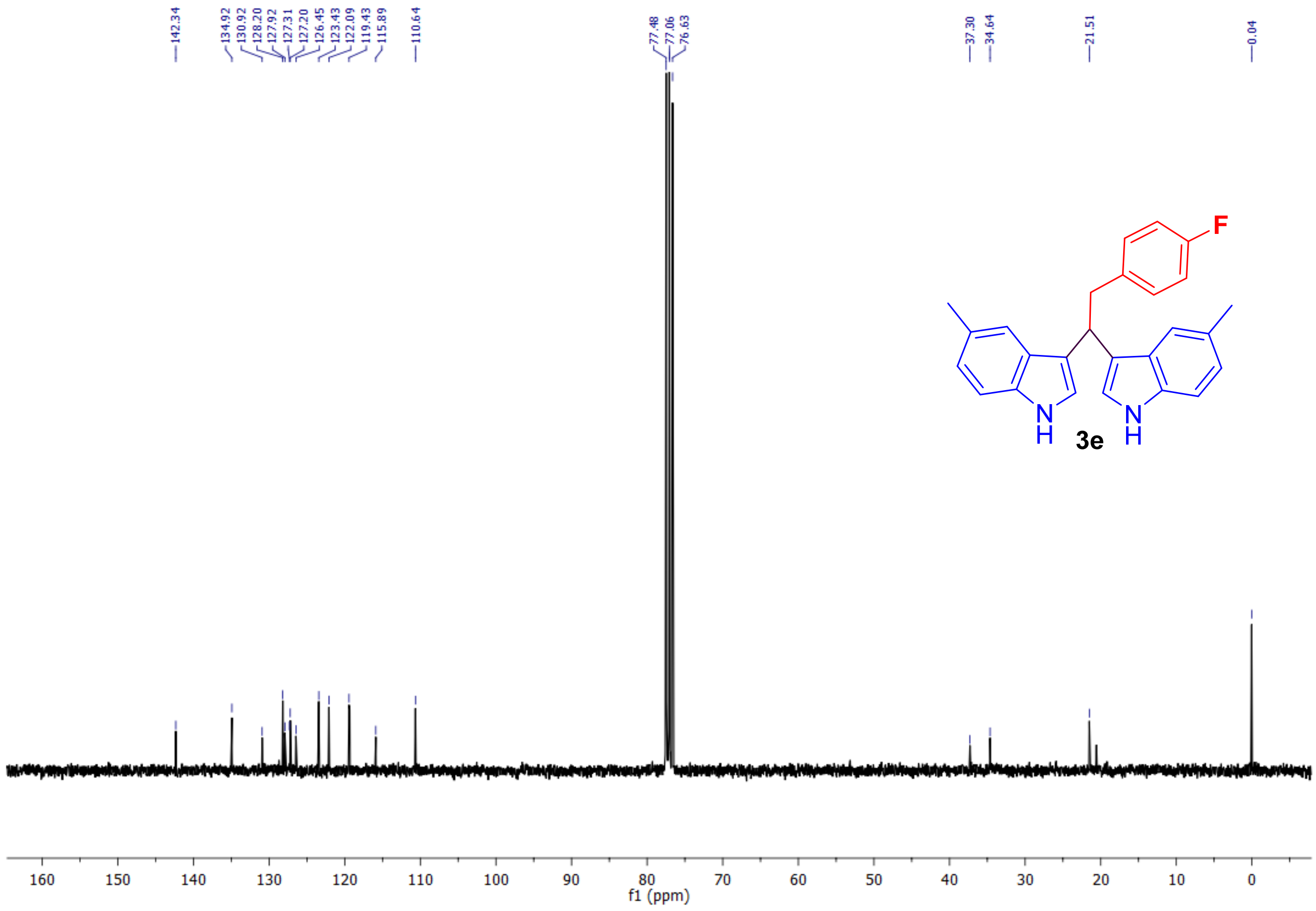

Figure S10. ^{13}C NMR Spectra of **3e** (300 MHz, $CDCl_3$)

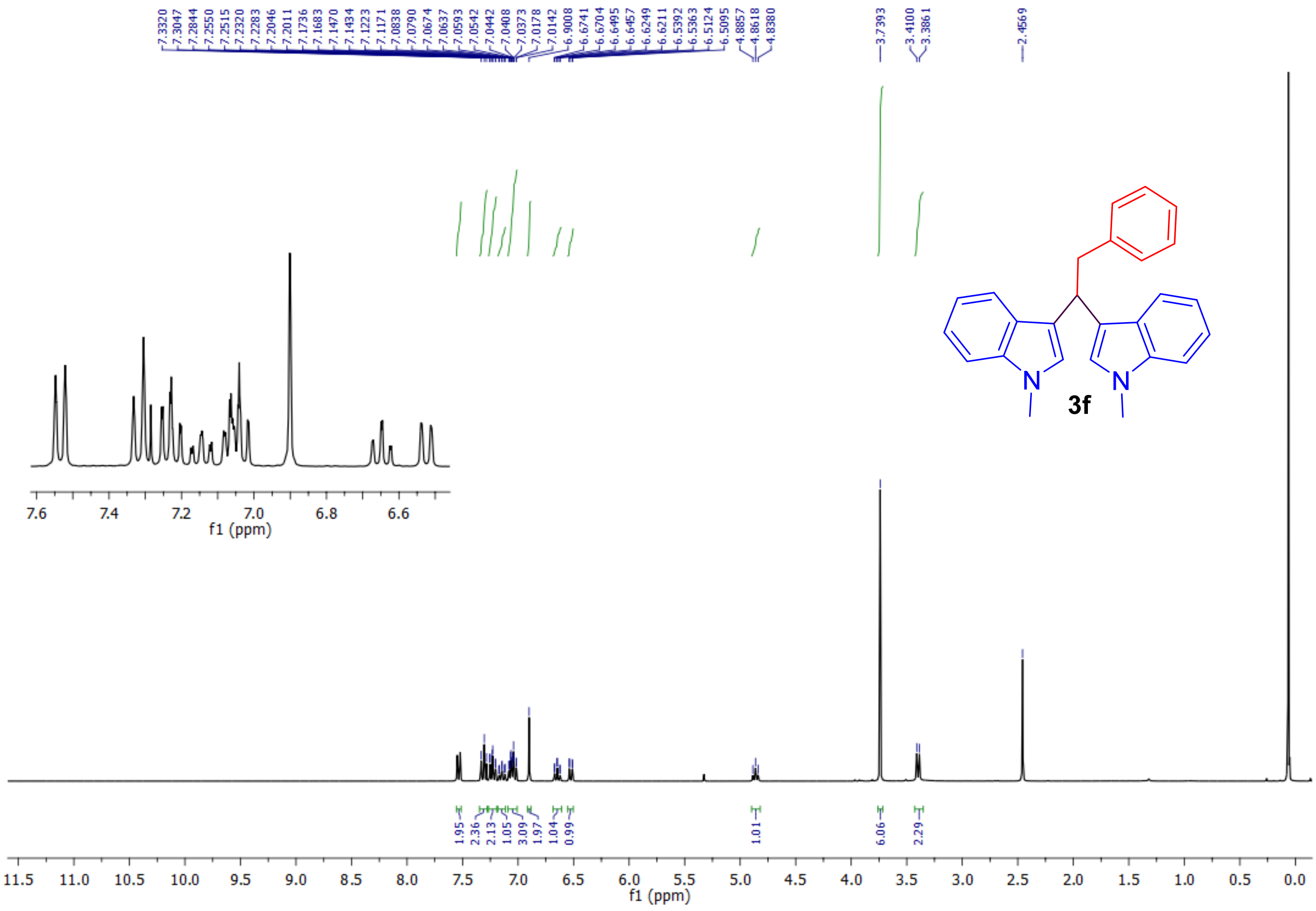

Figure S11. ^{1}H NMR Spectra of **3f** (300 MHz, $CDCl_3$)

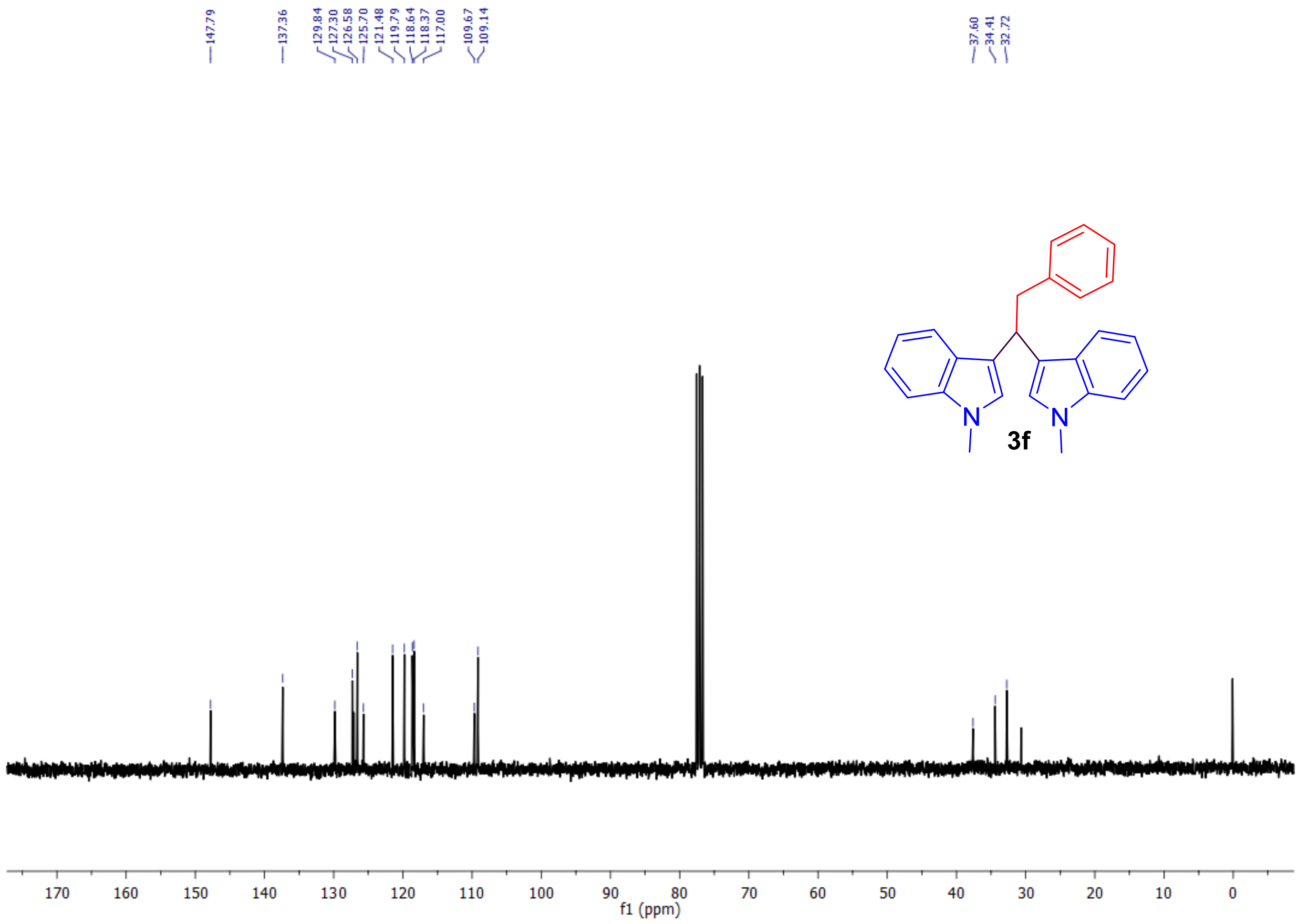

Figure S12. ^{13}C NMR Spectra of **3f** (300 MHz, $CDCl_3$)

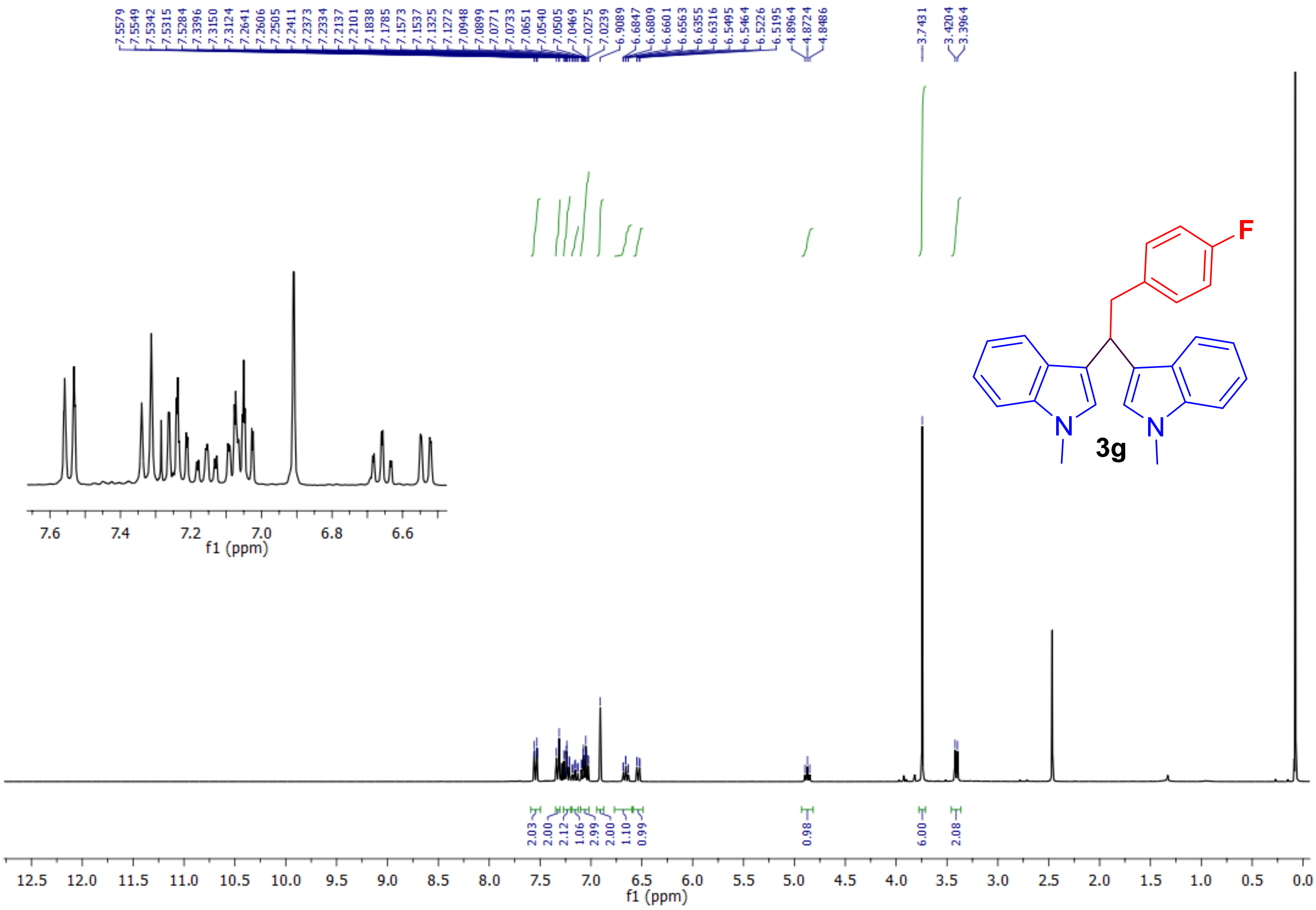

Figure S13. ^{1}H NMR Spectra of **3g** (300 MHz, $CDCl_3$)

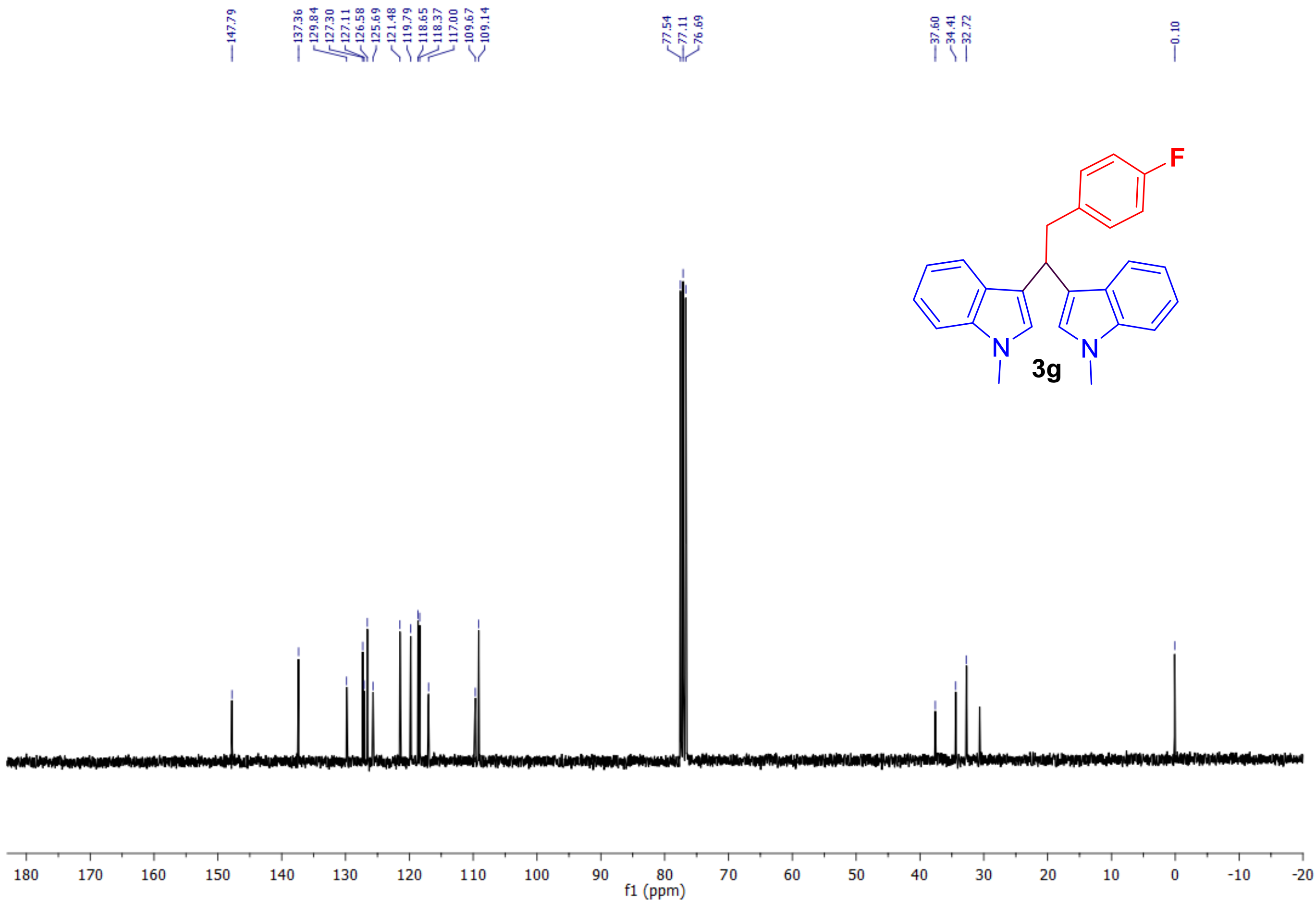

Figure S14. ^{13}C NMR Spectra of **3g** (300 MHz, $CDCl_3$)

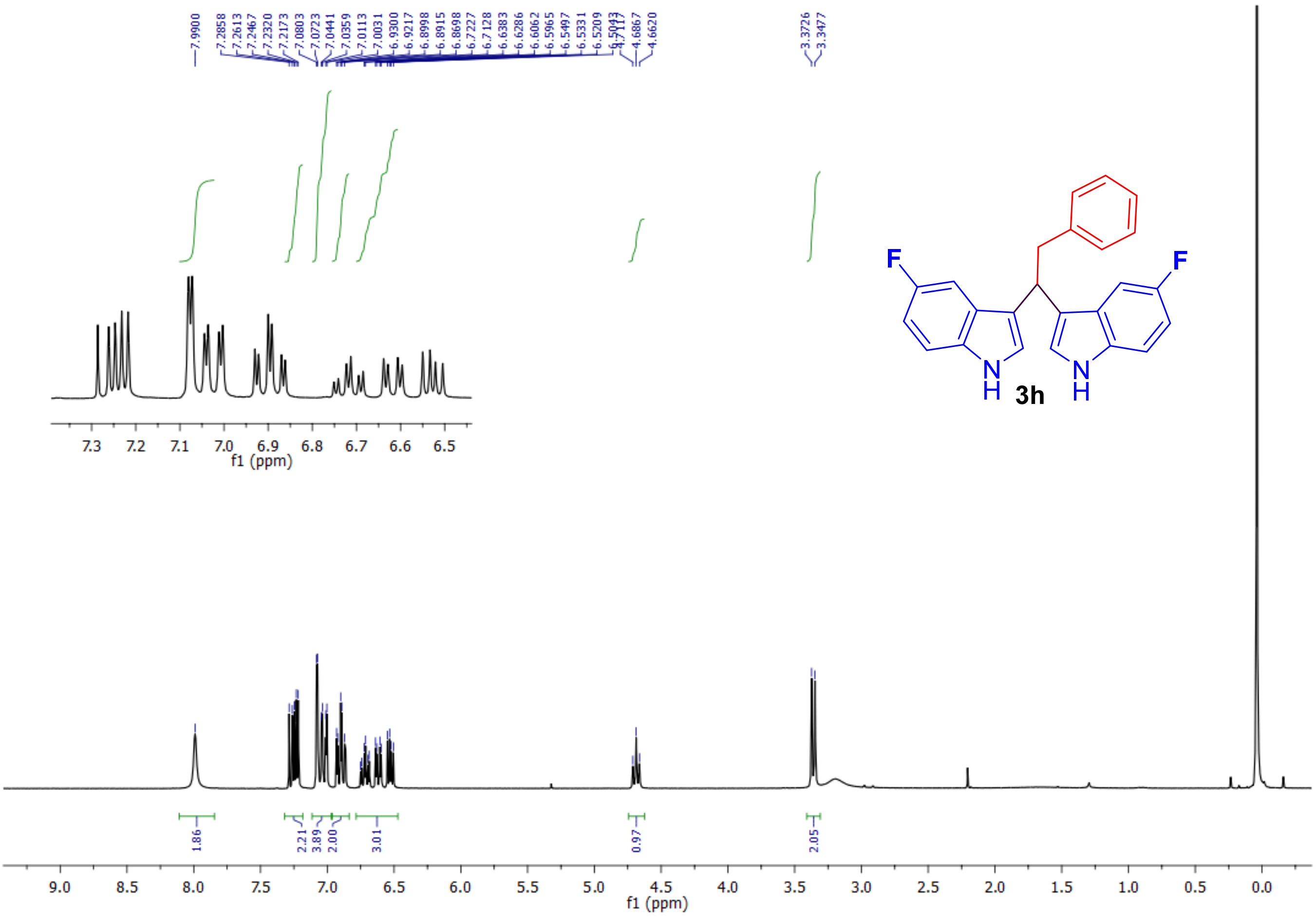

Figure S15. ^{1}H NMR Spectra of **3h** (300 MHz, $CDCl_3$)

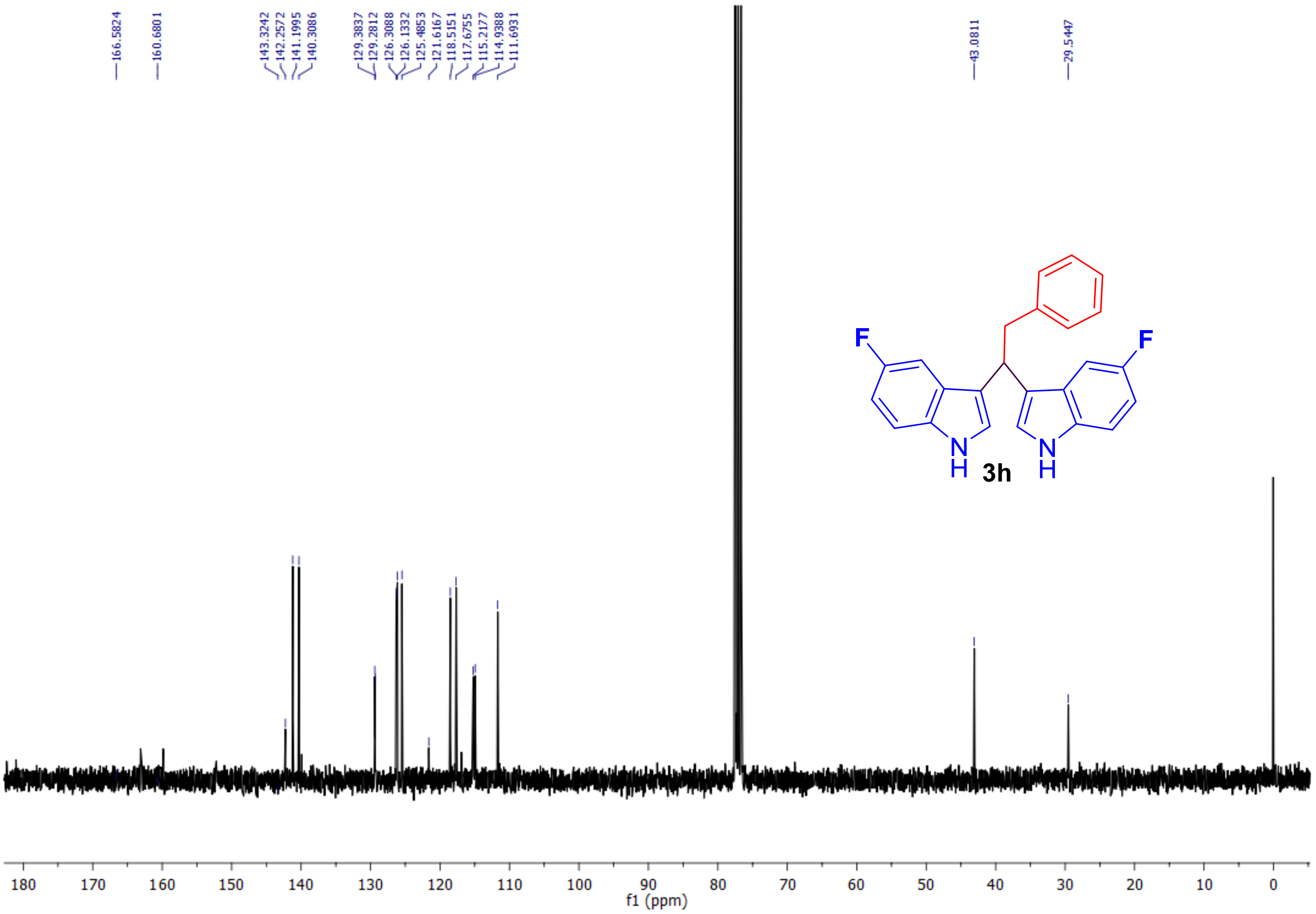

Figure S16. ^{13}C NMR Spectra of **3h** (300 MHz, acetone-d$_6$)

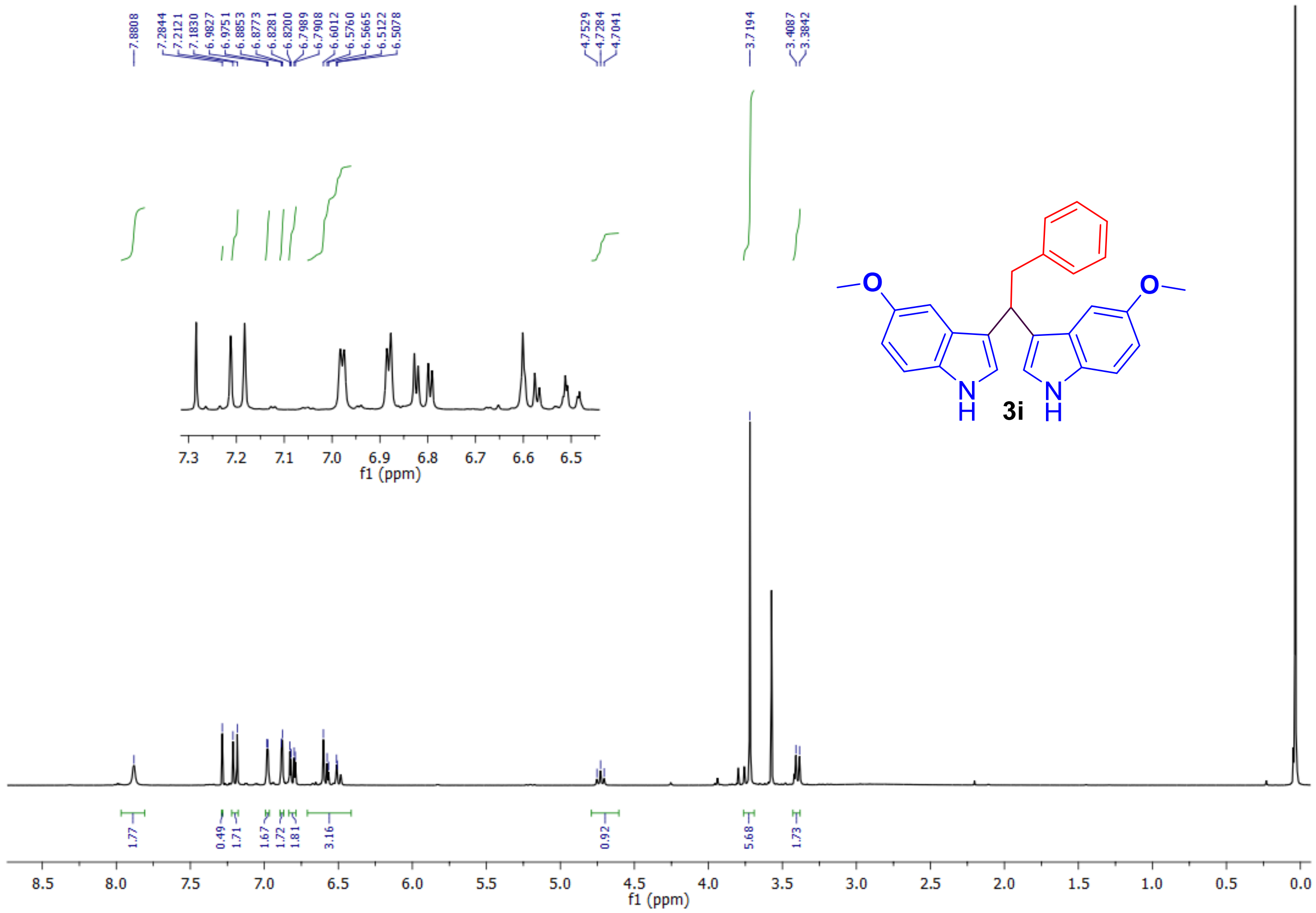

Figure S17. ^{1}H NMR Spectra of **3i** (300 MHz, $CDCl_3$)

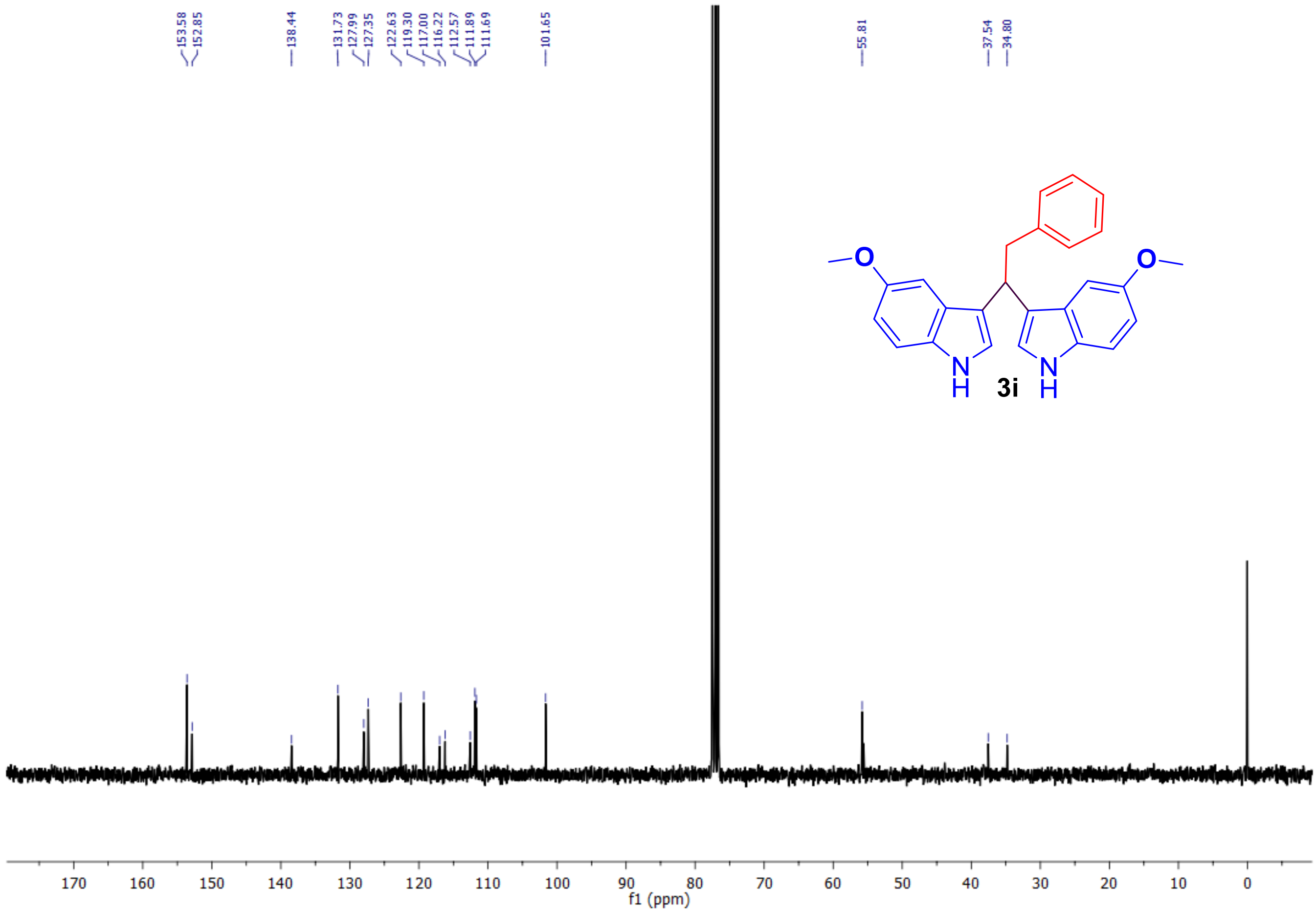

Figure S18. ^{13}C NMR Spectra of **3i** (300 MHz, $CDCl_3$)

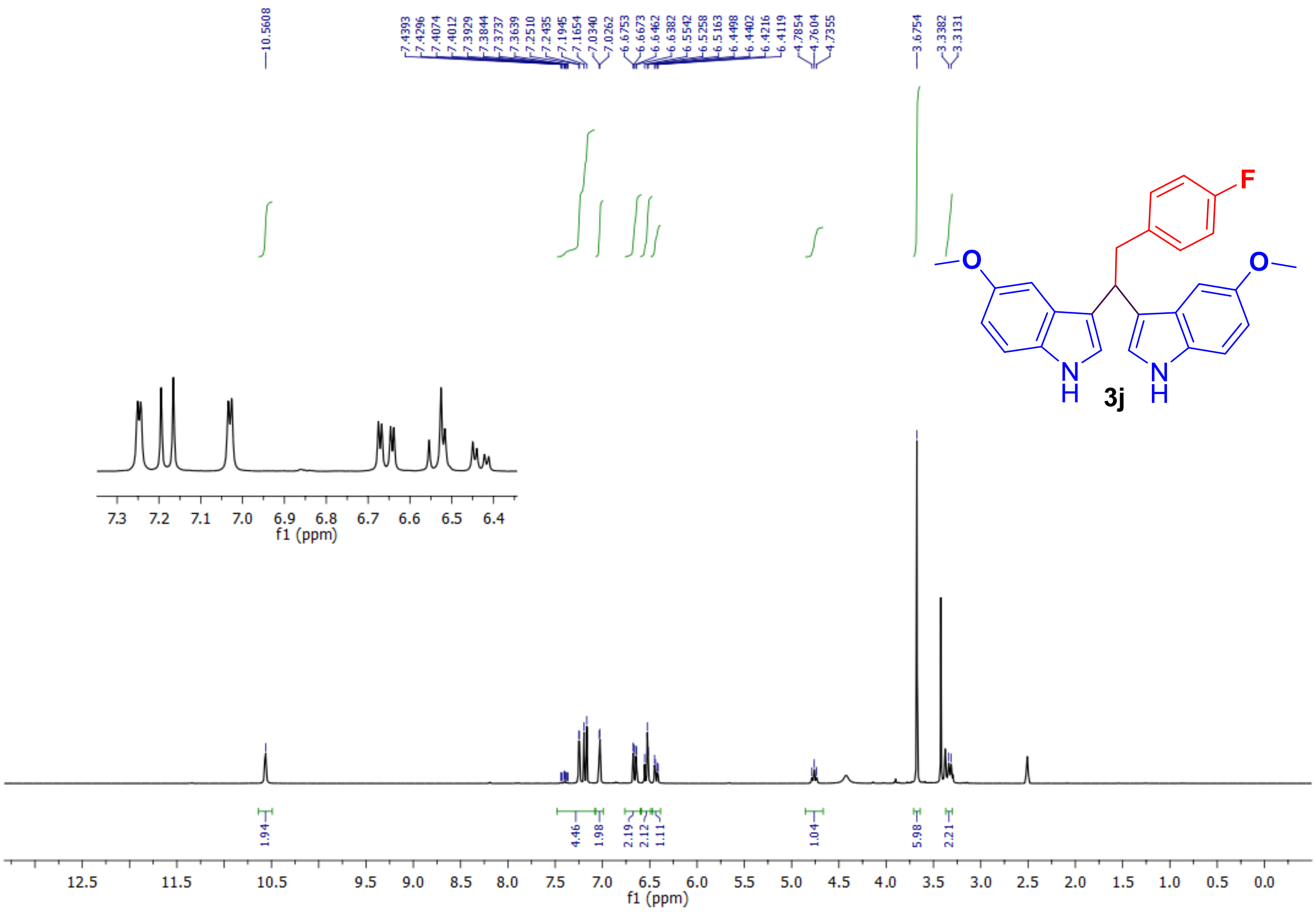

Figure S19. ^{1}H NMR Spectra of **3j** (300 MHz, $CDCl_3$)

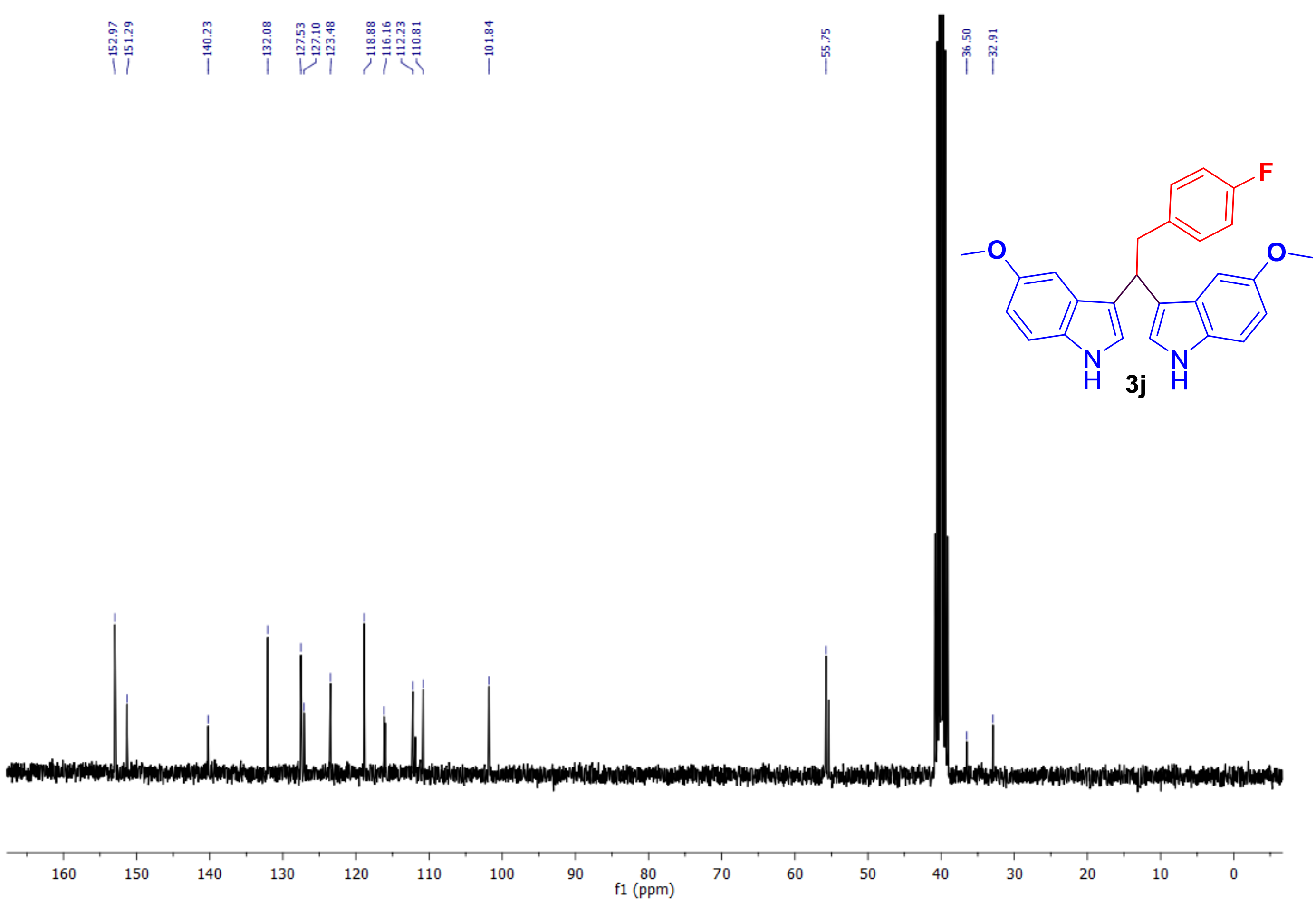

Figure S20. ^{13}C NMR Spectra of **3j** (300 MHz, $CDCl_3$)

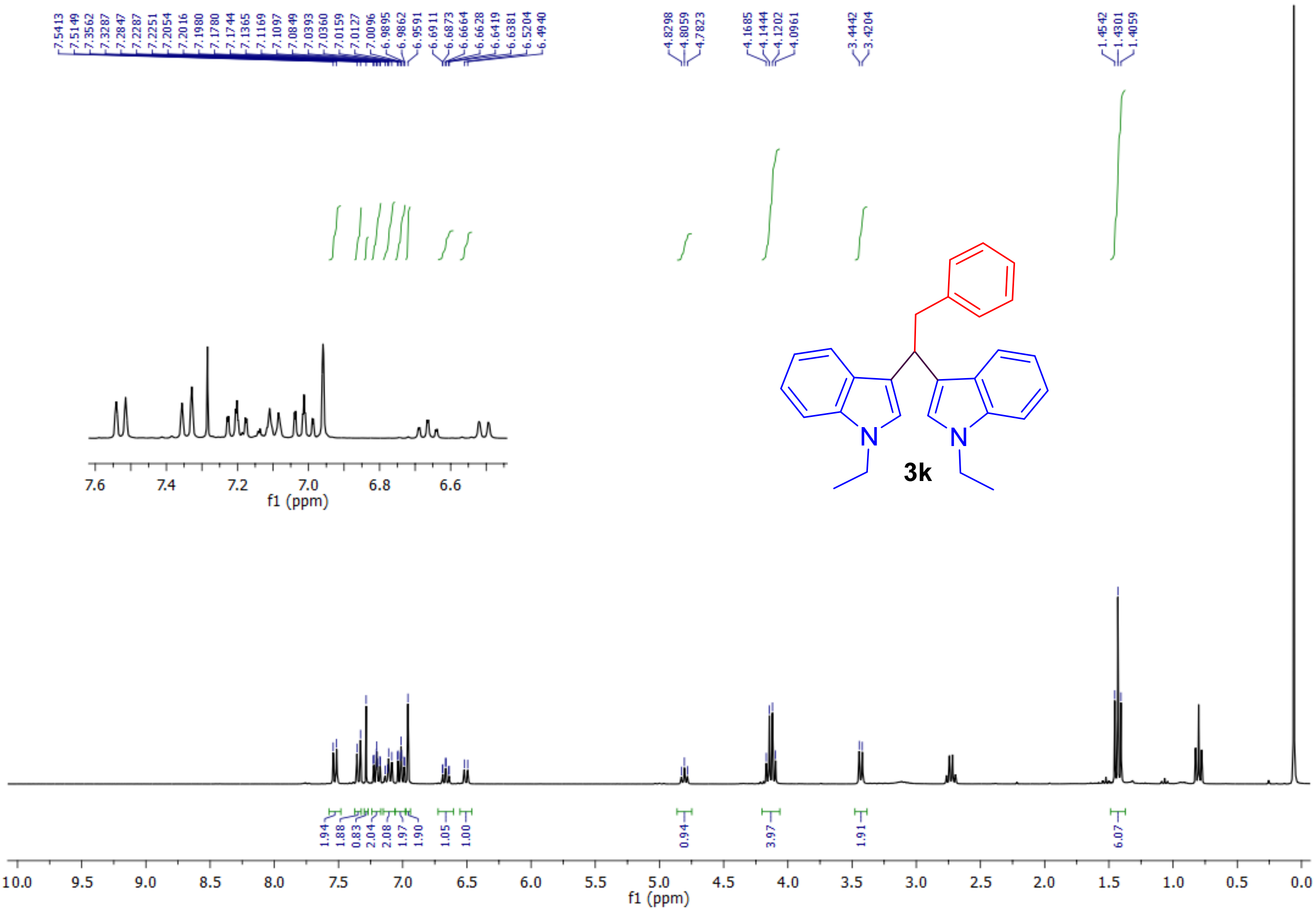

Figure S21. ^{1}H NMR Spectra of **3k** (300 MHz, $CDCl_3$)

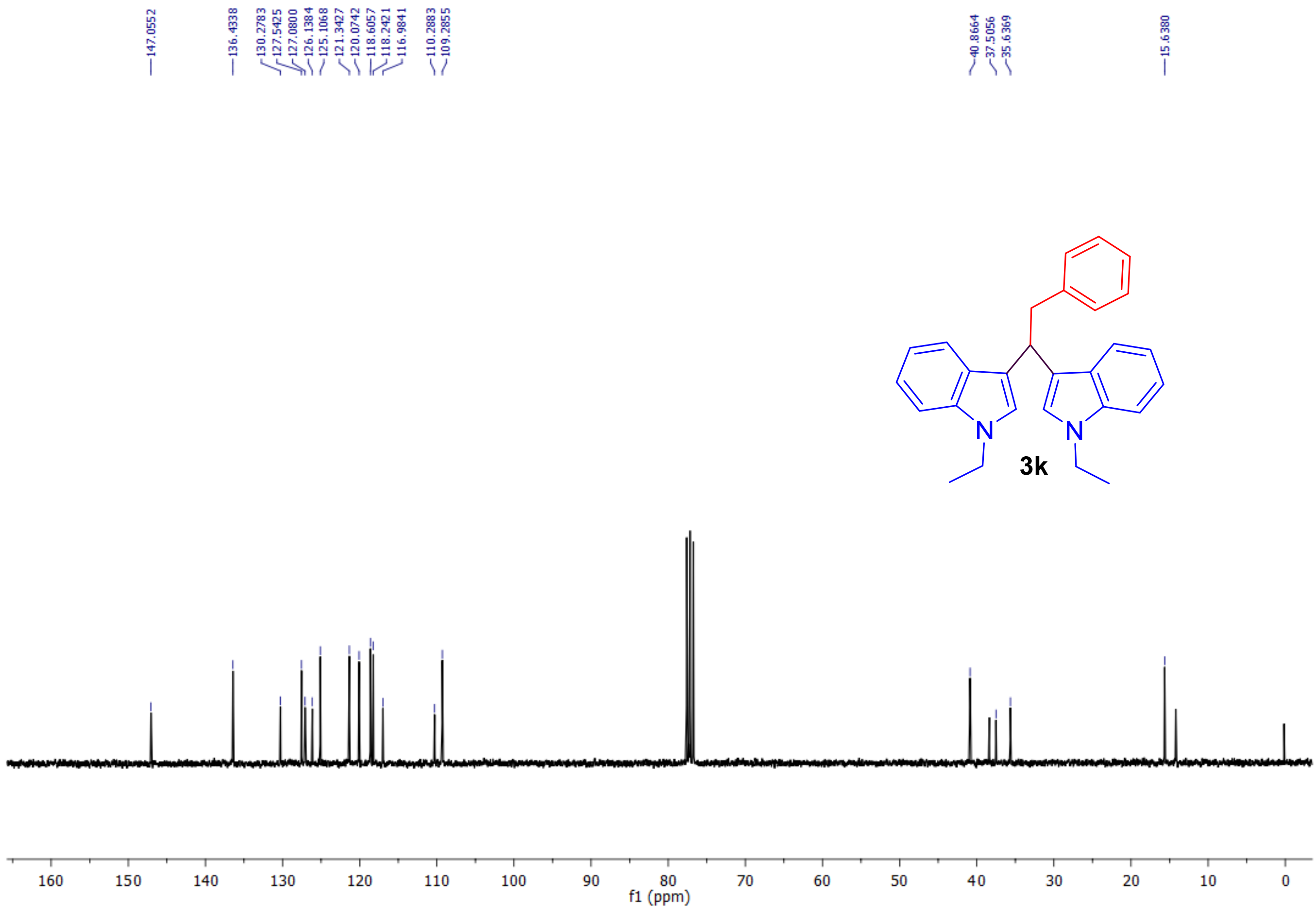

Figure S22. ^{13}C NMR Spectra of **3k** (300 MHz, $CDCl_3$)

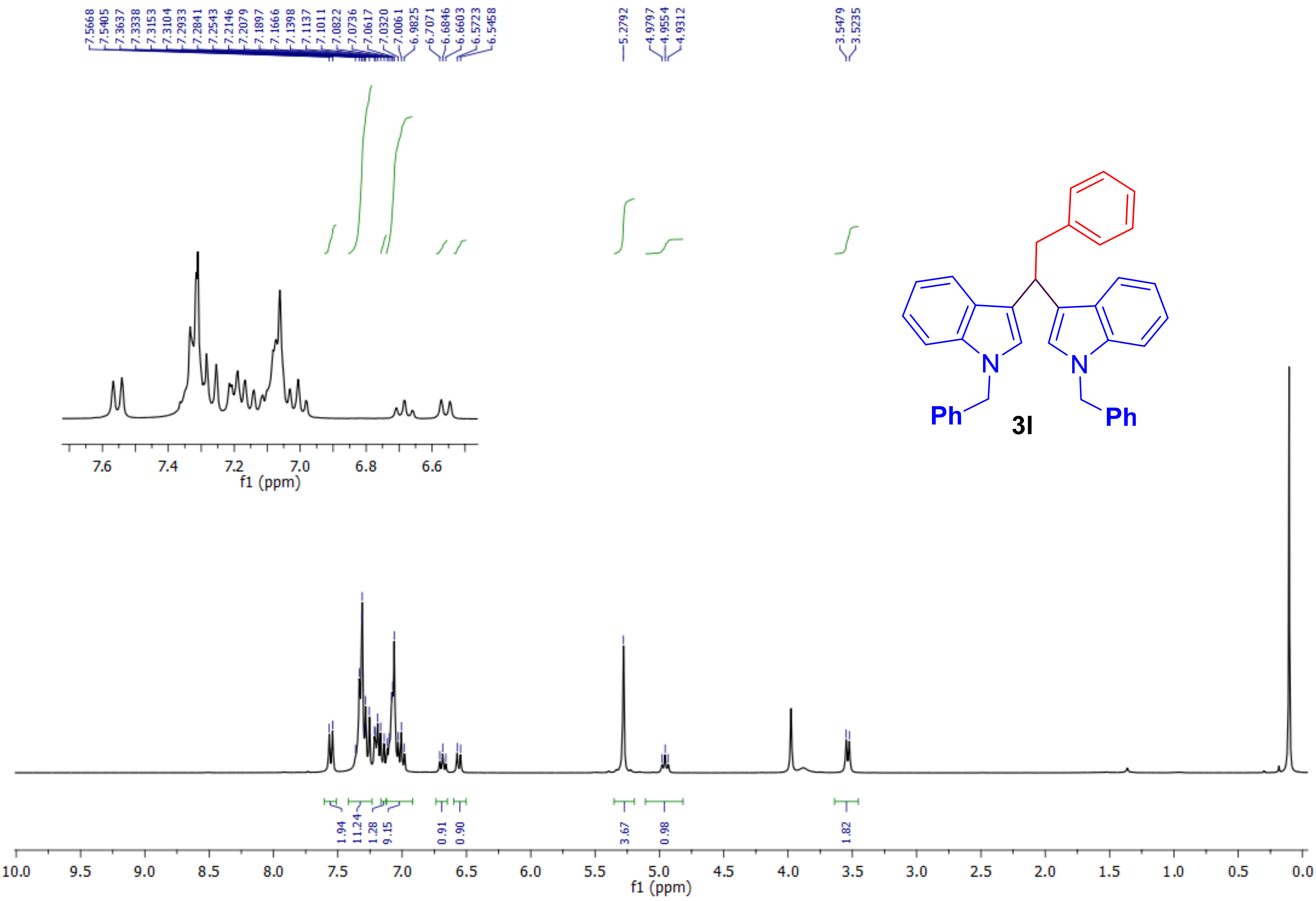

Figure S23. ^{1}H NMR Spectra of **3l** (300 MHz, $CDCl_3$)

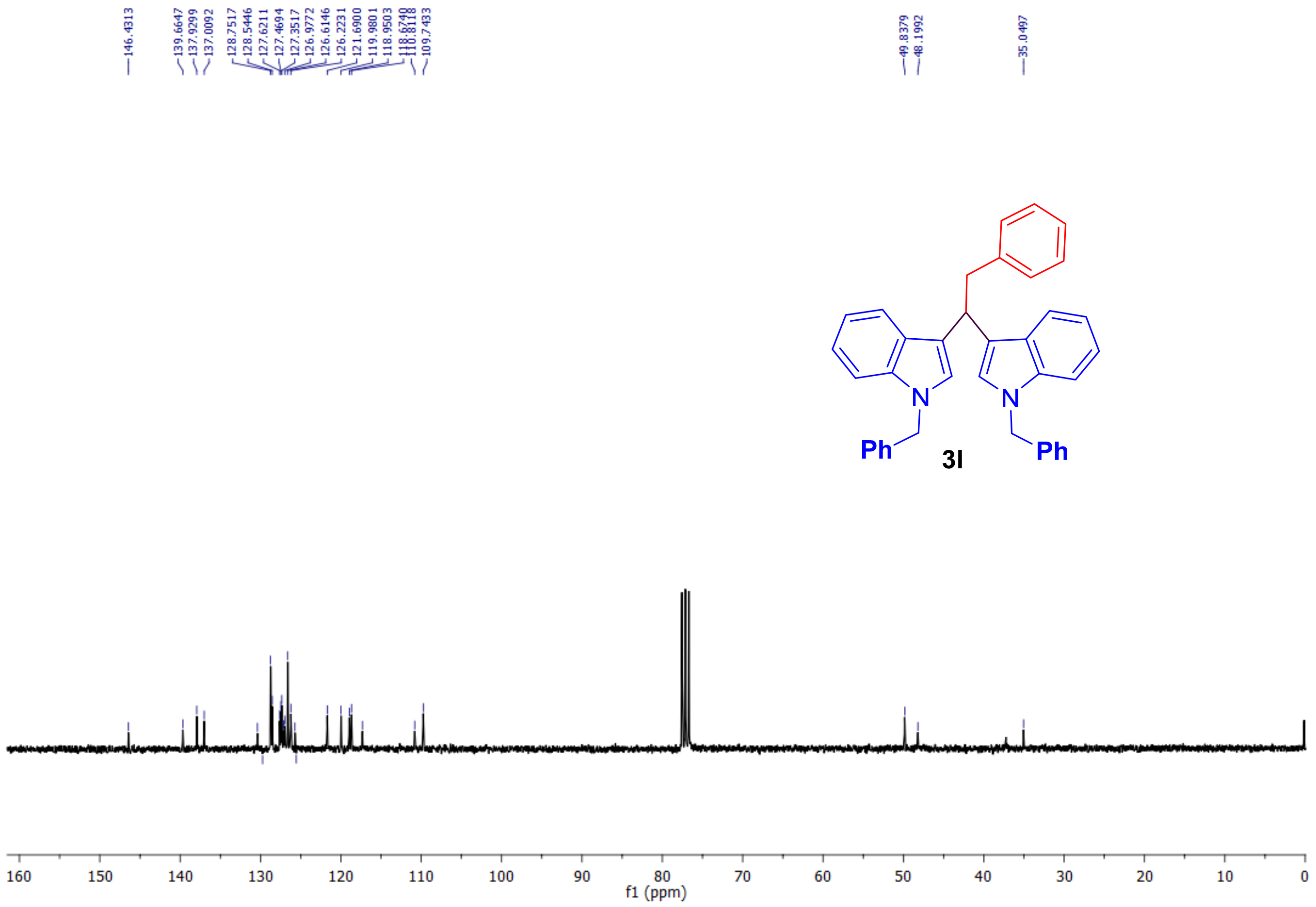

Figure S24. ^{13}C NMR Spectra of **3l** (300 MHz, $CDCl_3$)

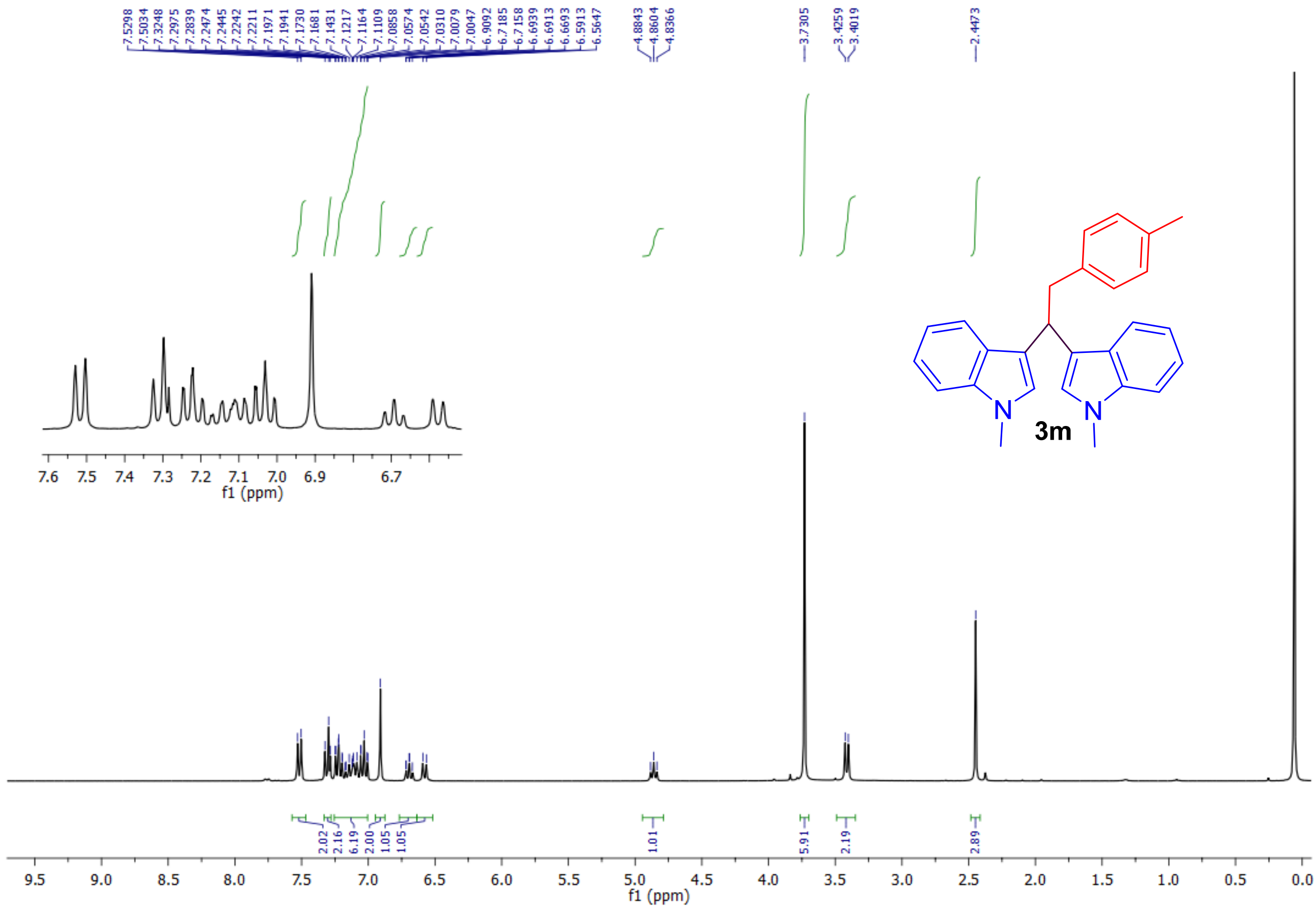

Figure S25. ^{1}H NMR Spectra of **3m** (300 MHz, $CDCl_3$)

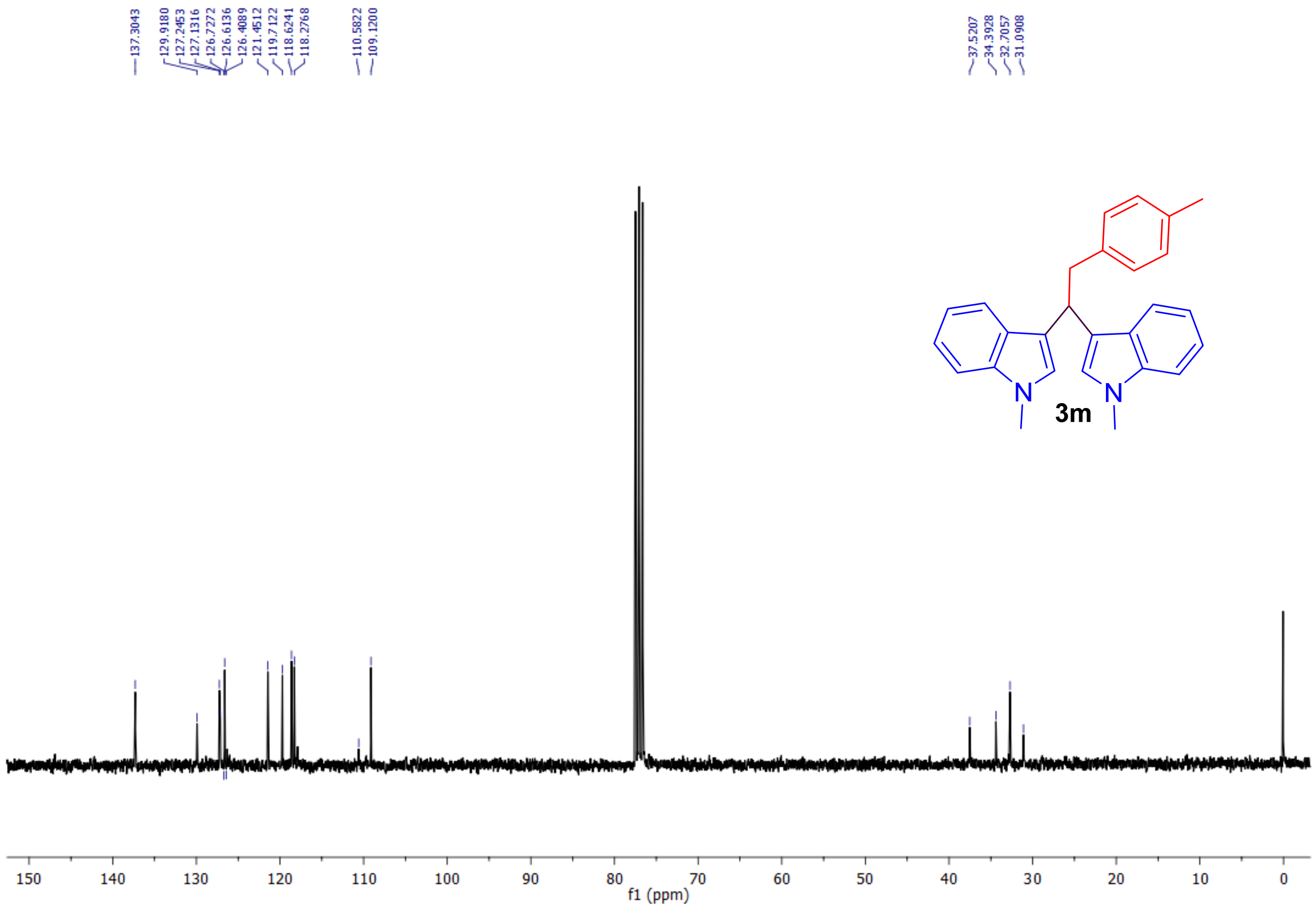

Figure S26. ^{13}C NMR Spectra of **3m** (300 MHz, $CDCl_3$)

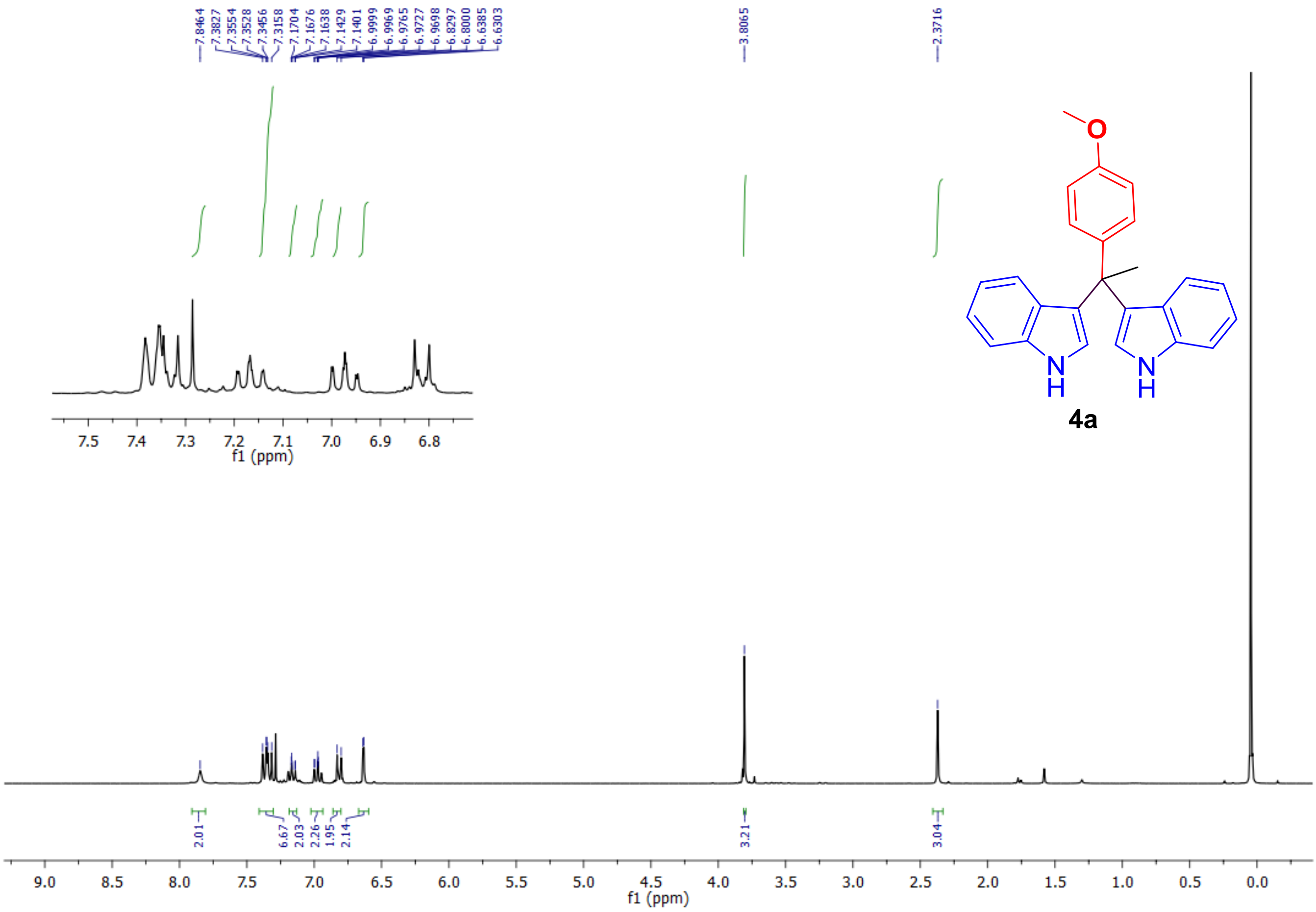

Figure S27. ^{1}H NMR Spectra of **4a** (300 MHz, $CDCl_3$)

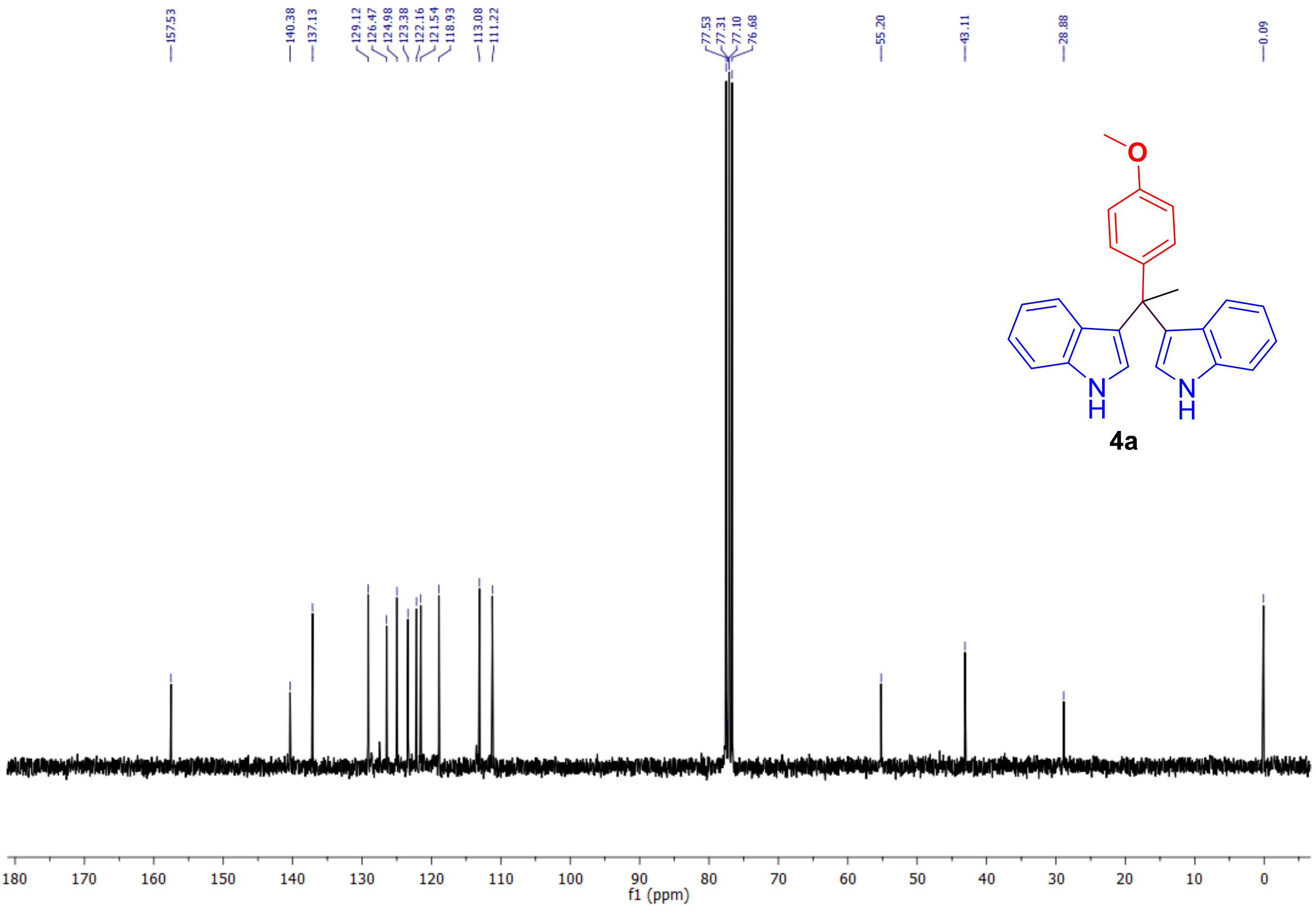

Figure S28. [13] C NMR Spectra of **4a** (300 MHz, $CDCl_3$)

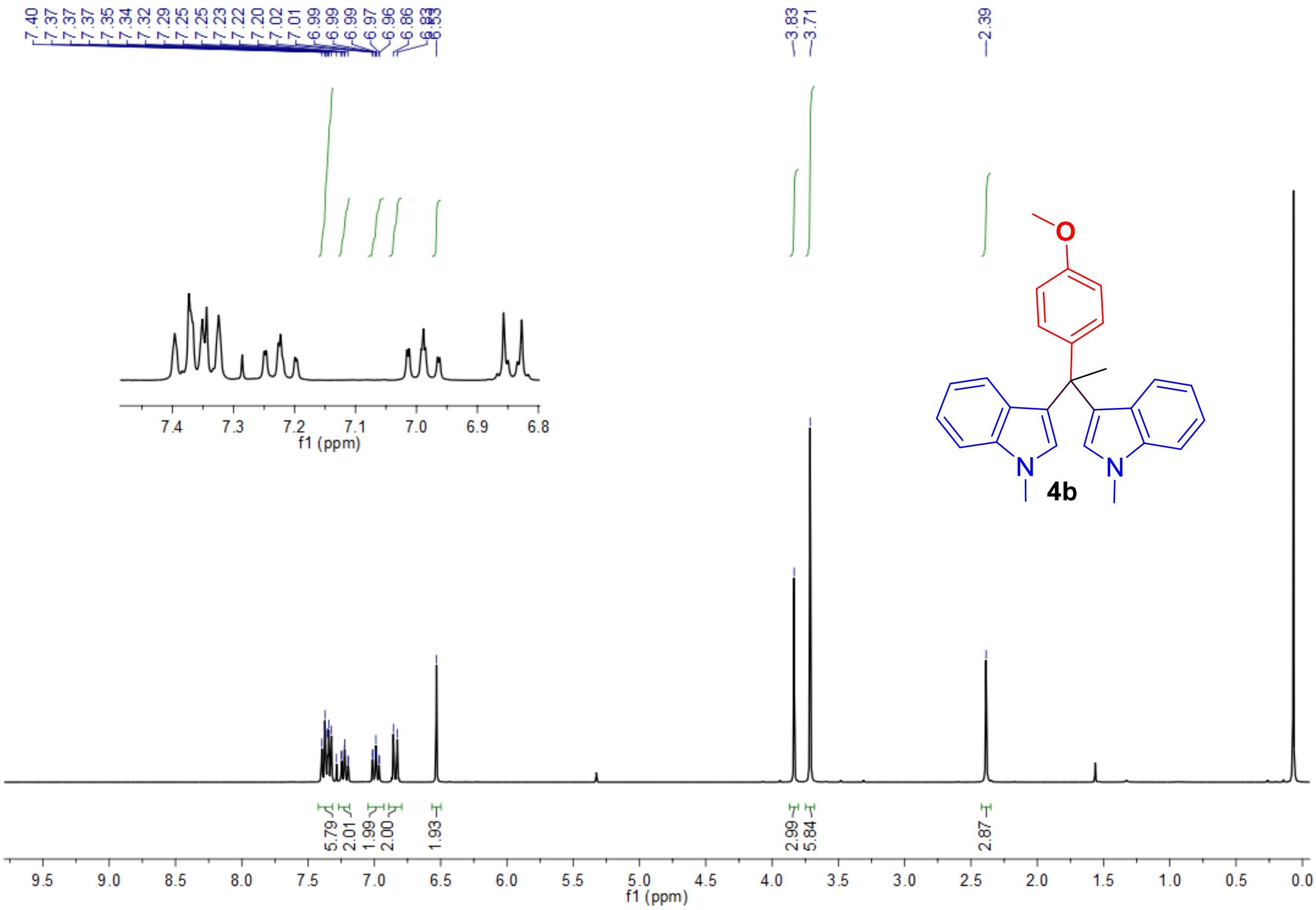

Figure S29. ^{1}H NMR Spectra of **4b** (300 MHz, $CDCl_3$)

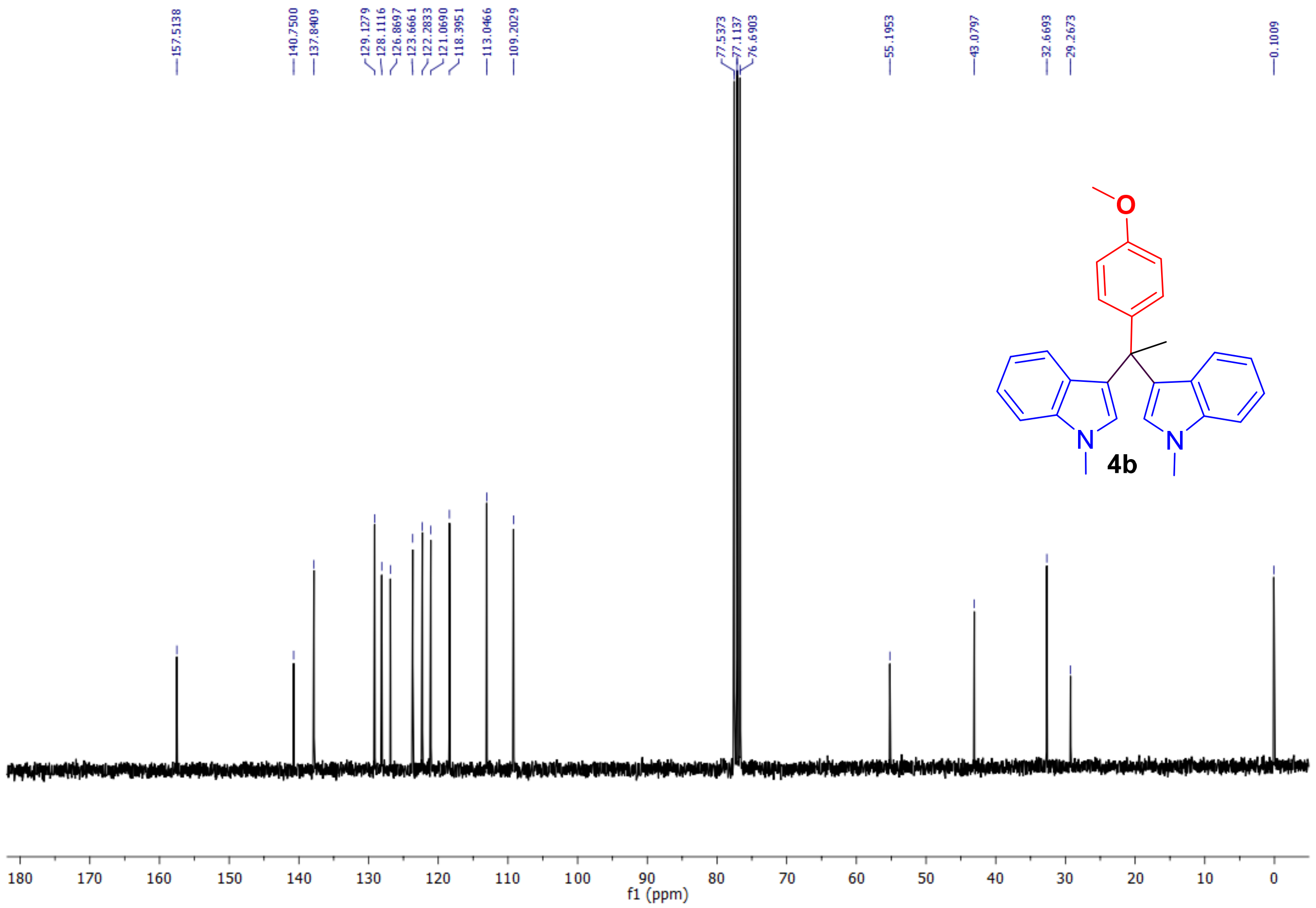

Figure S30. ^{13}C NMR Spectra of **4b** (300 MHz, $CDCl_3$)

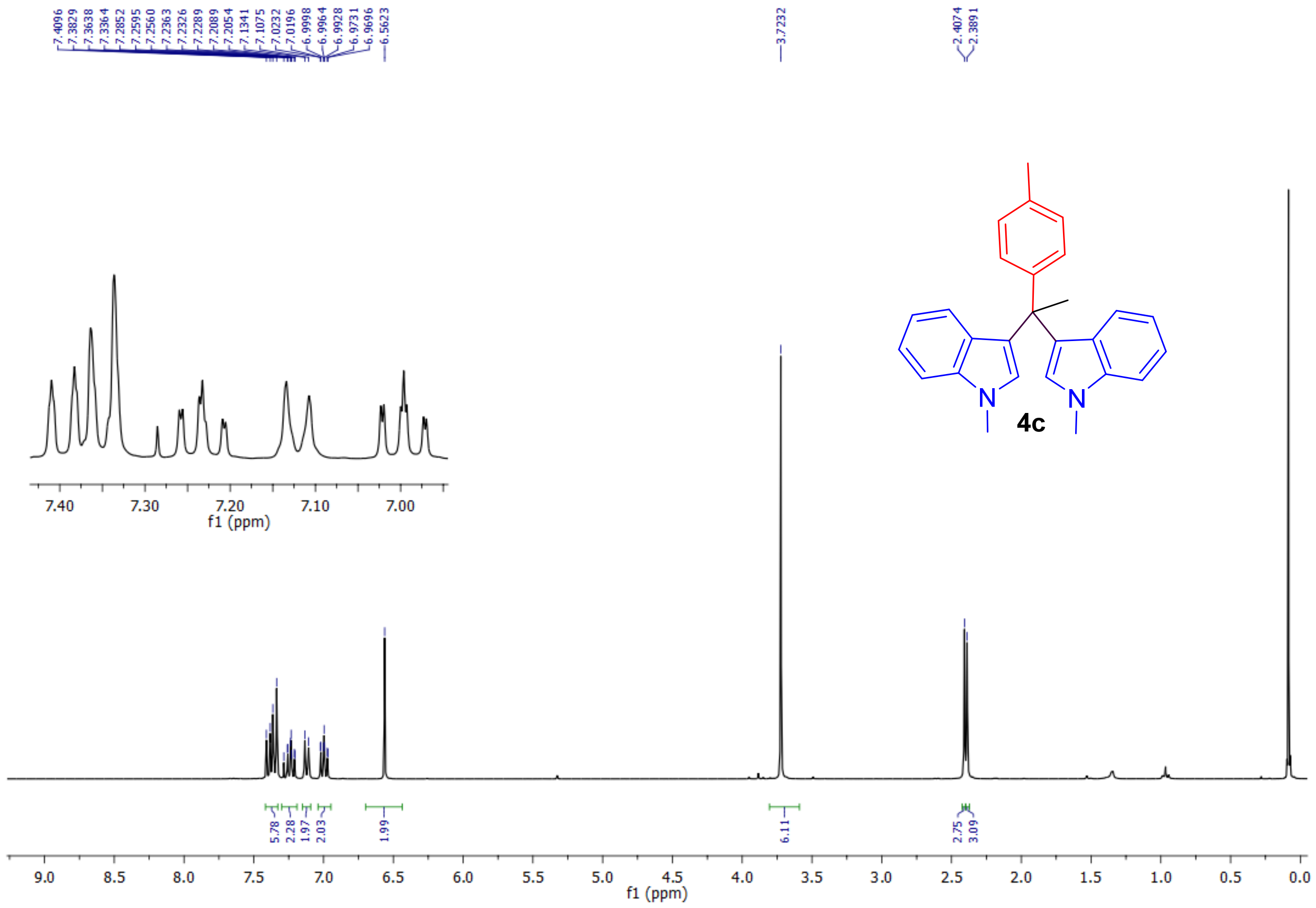

Figure S31. ^{1}H NMR Spectra of **4c** (300 MHz, $CDCl_3$)

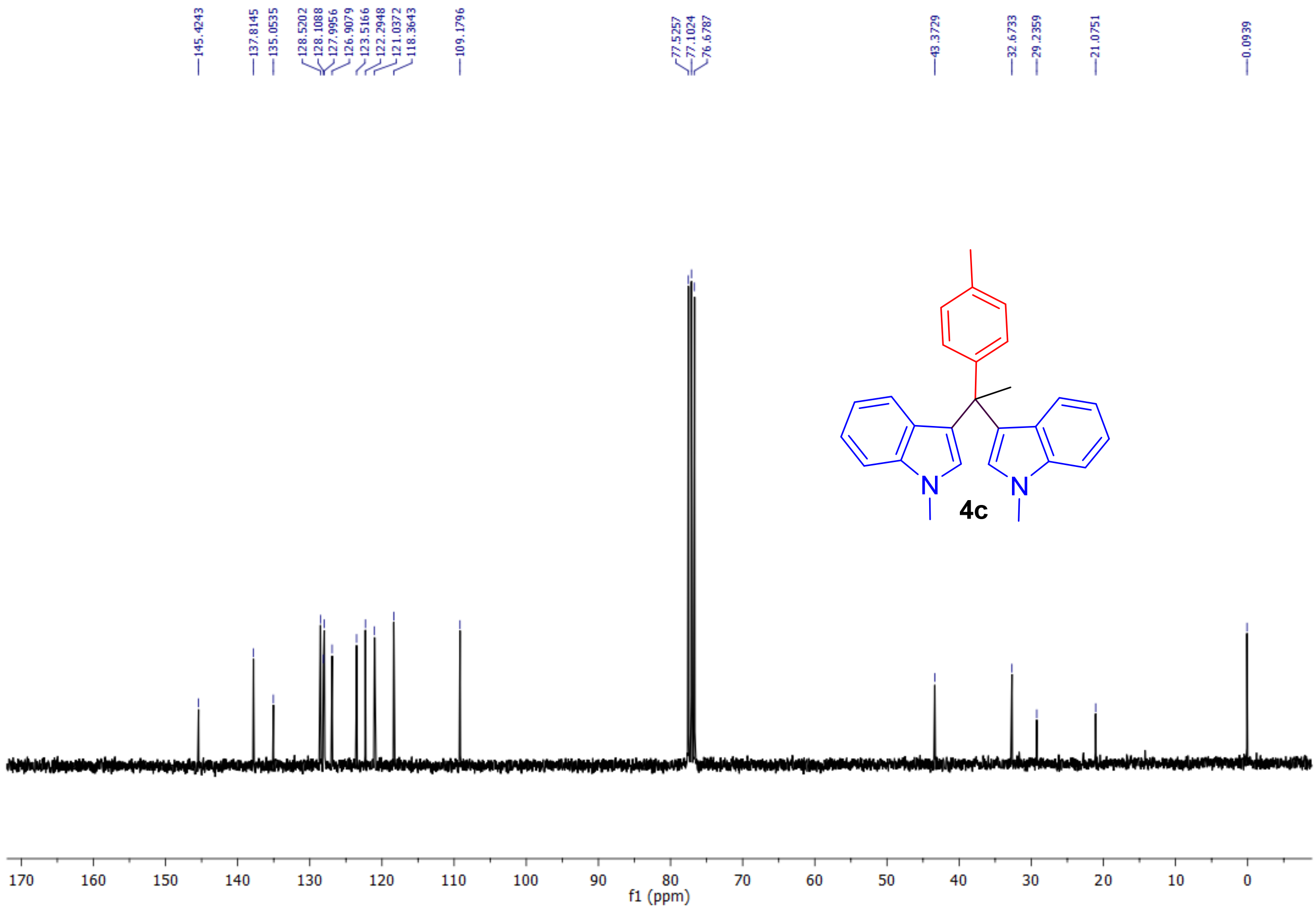

Figure S32. ^{13}C NMR Spectra of **4c** (300 MHz, $CDCl_3$)

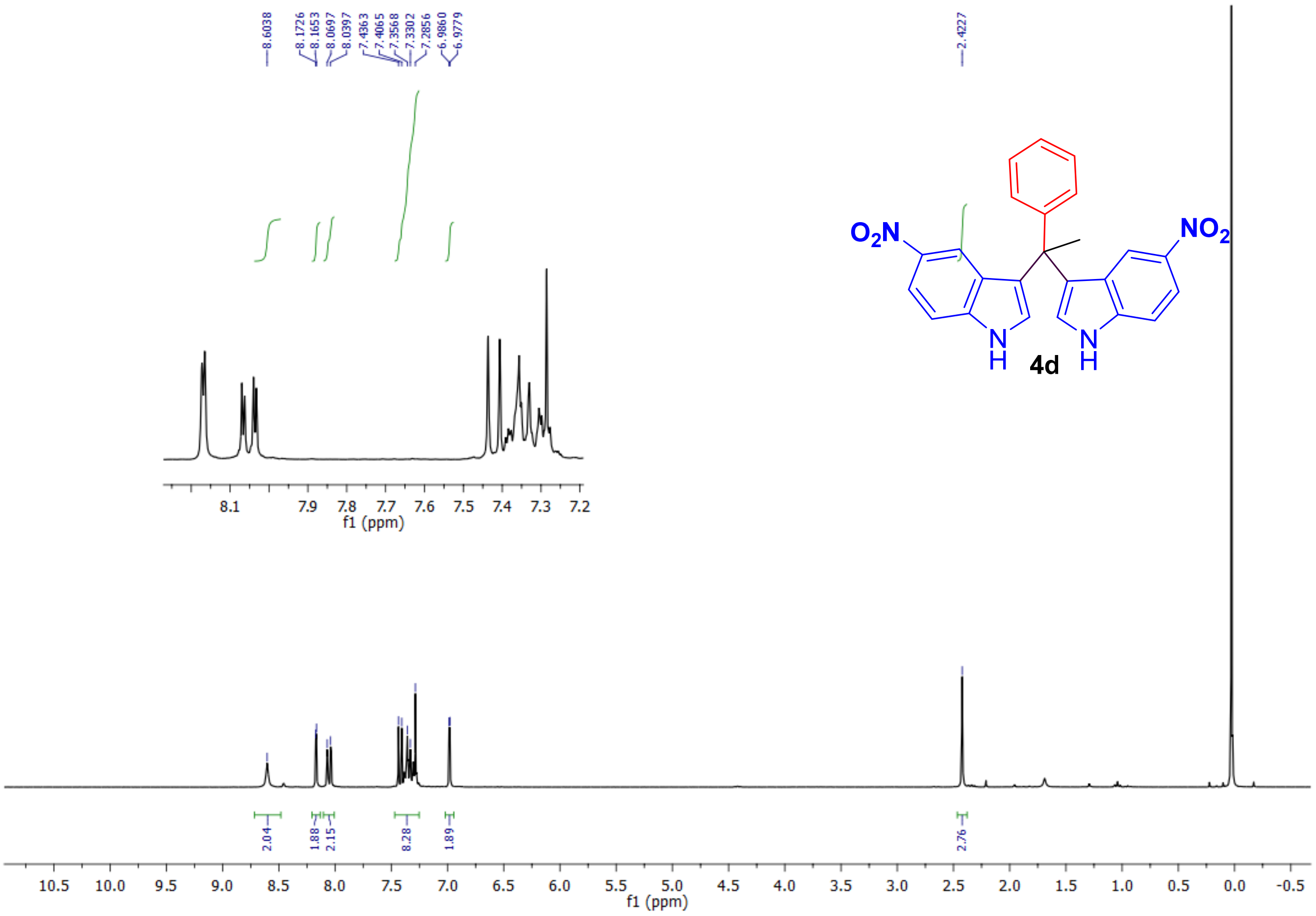

Figure S33. ^{1}H NMR Spectra of **4d** (300 MHz, $CDCl_3$)

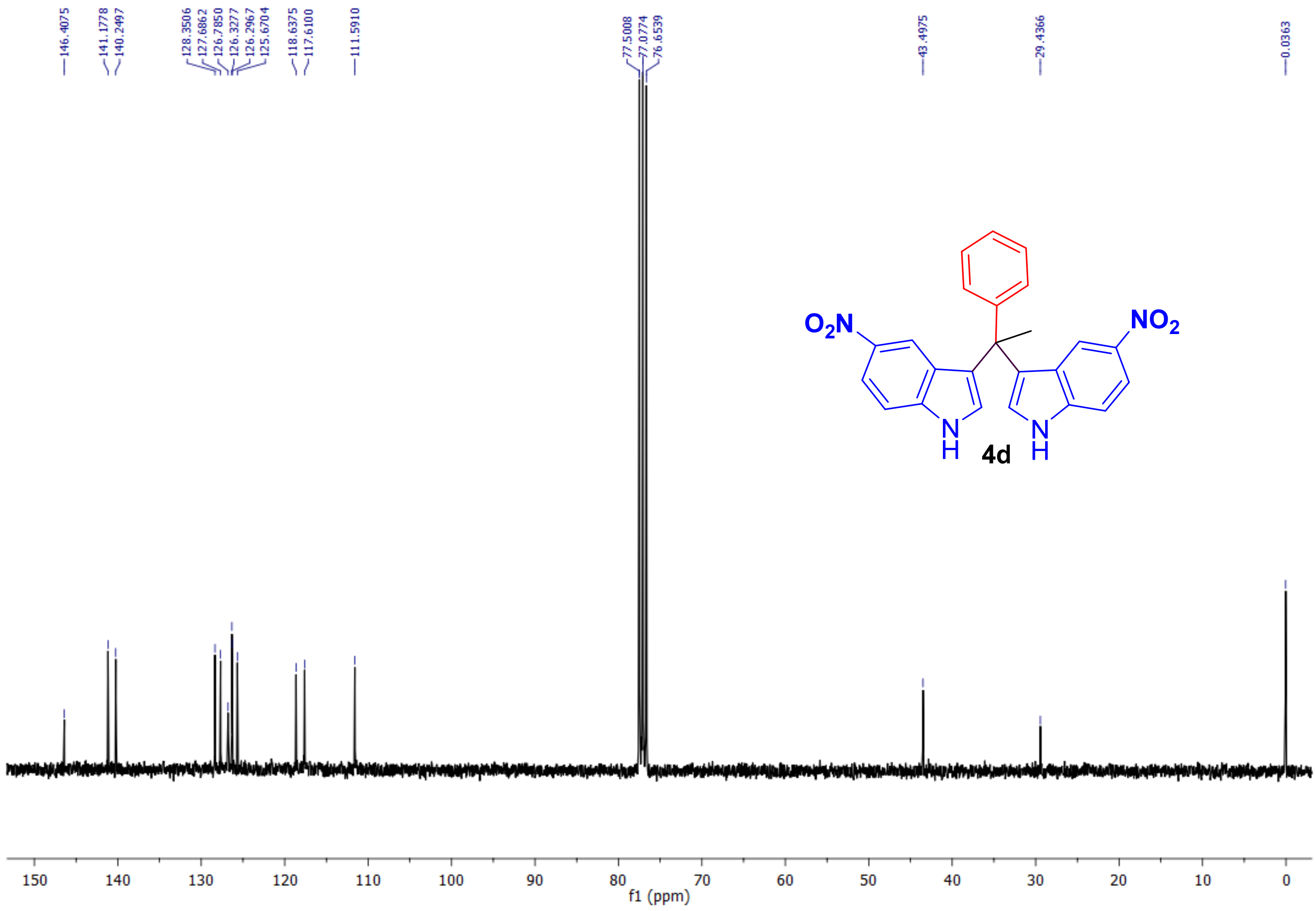

Figure S34. ^{13}C NMR Spectra of **4d** (300 MHz, $CDCl_3$)

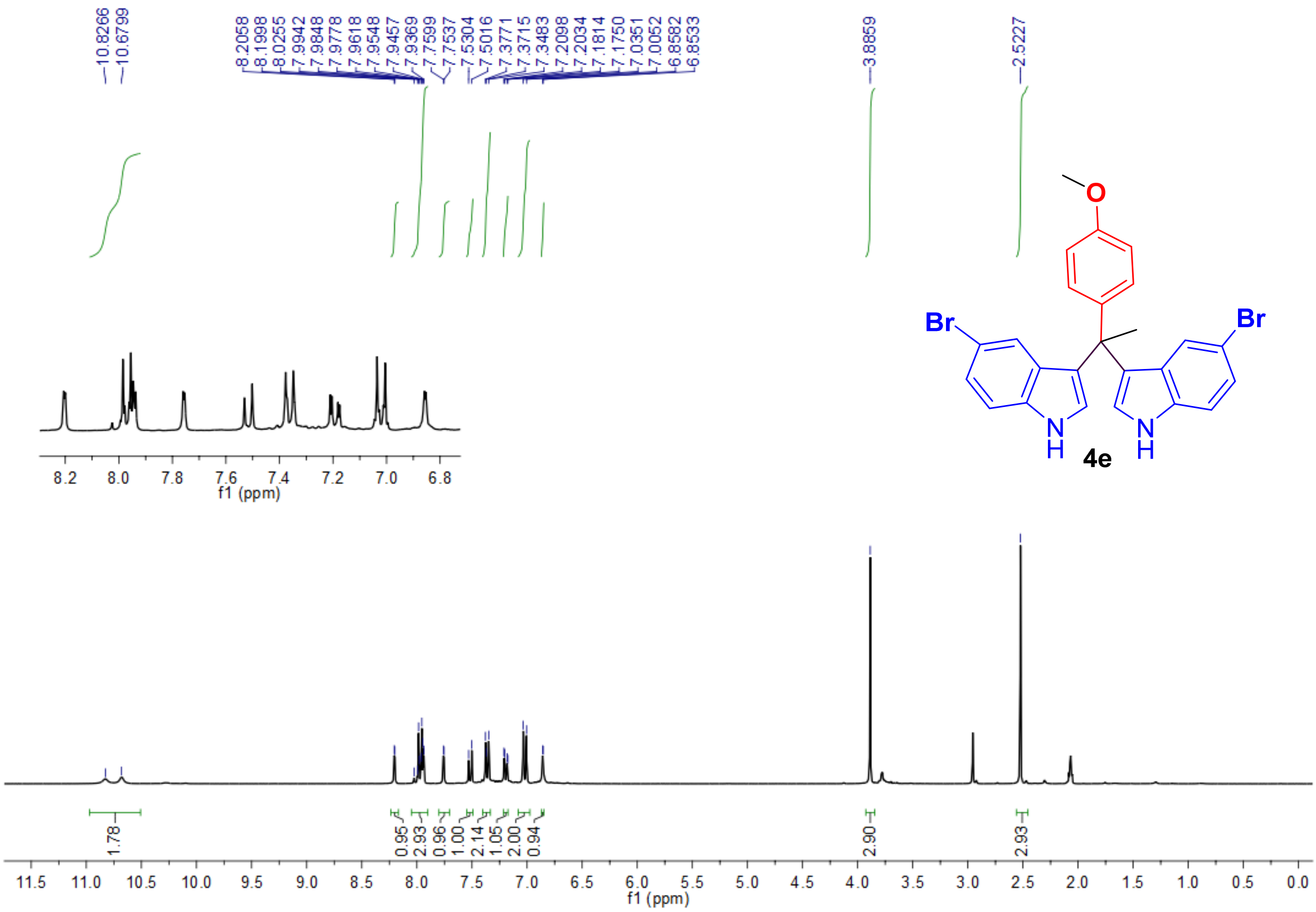

Figure S35. ^{1}H NMR Spectra of **4e** (300 MHz, acetone-d_6)

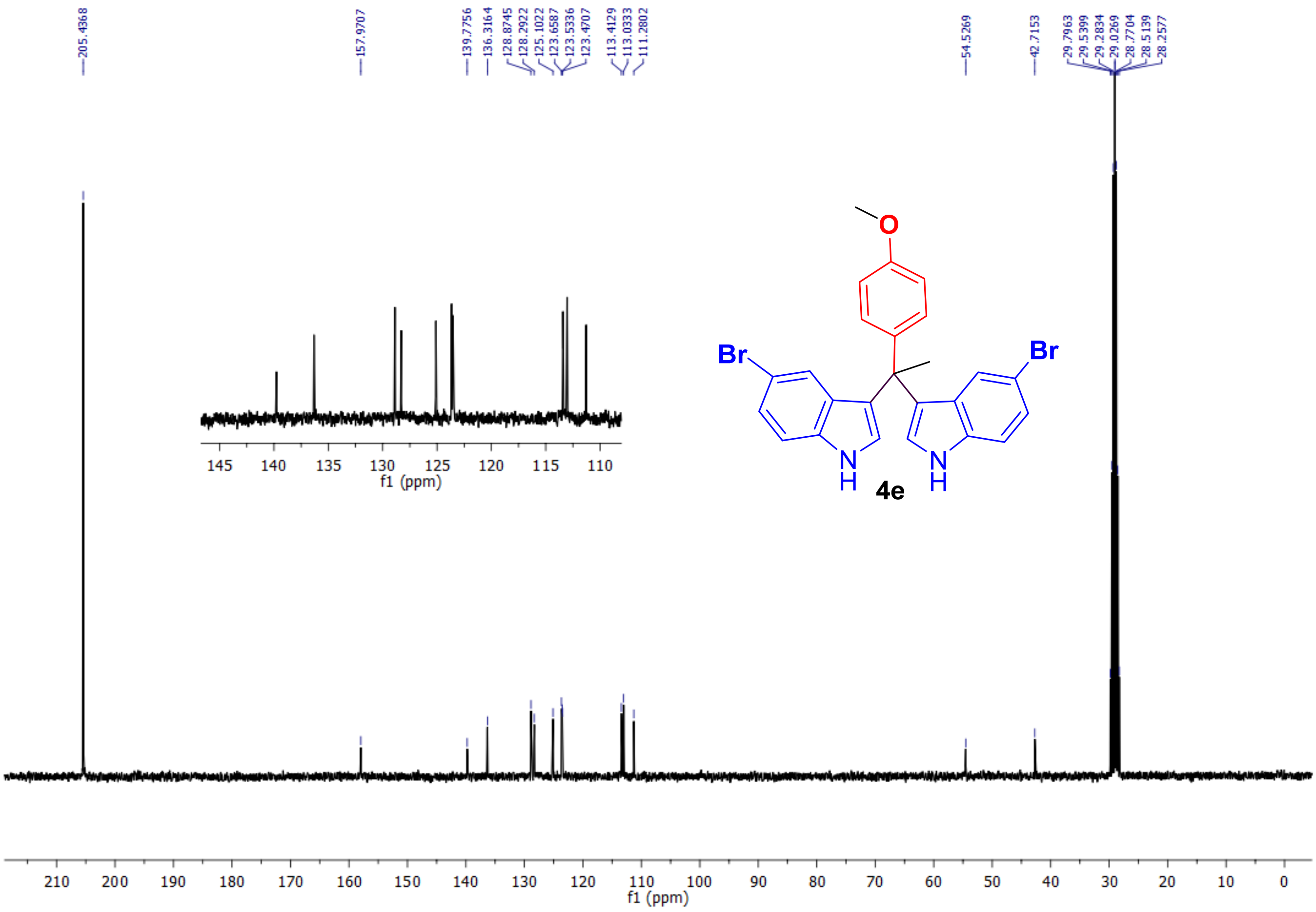

Figure S36. ^{13}C NMR Spectra of **4e** (300 MHz, acetone-d$_6$)

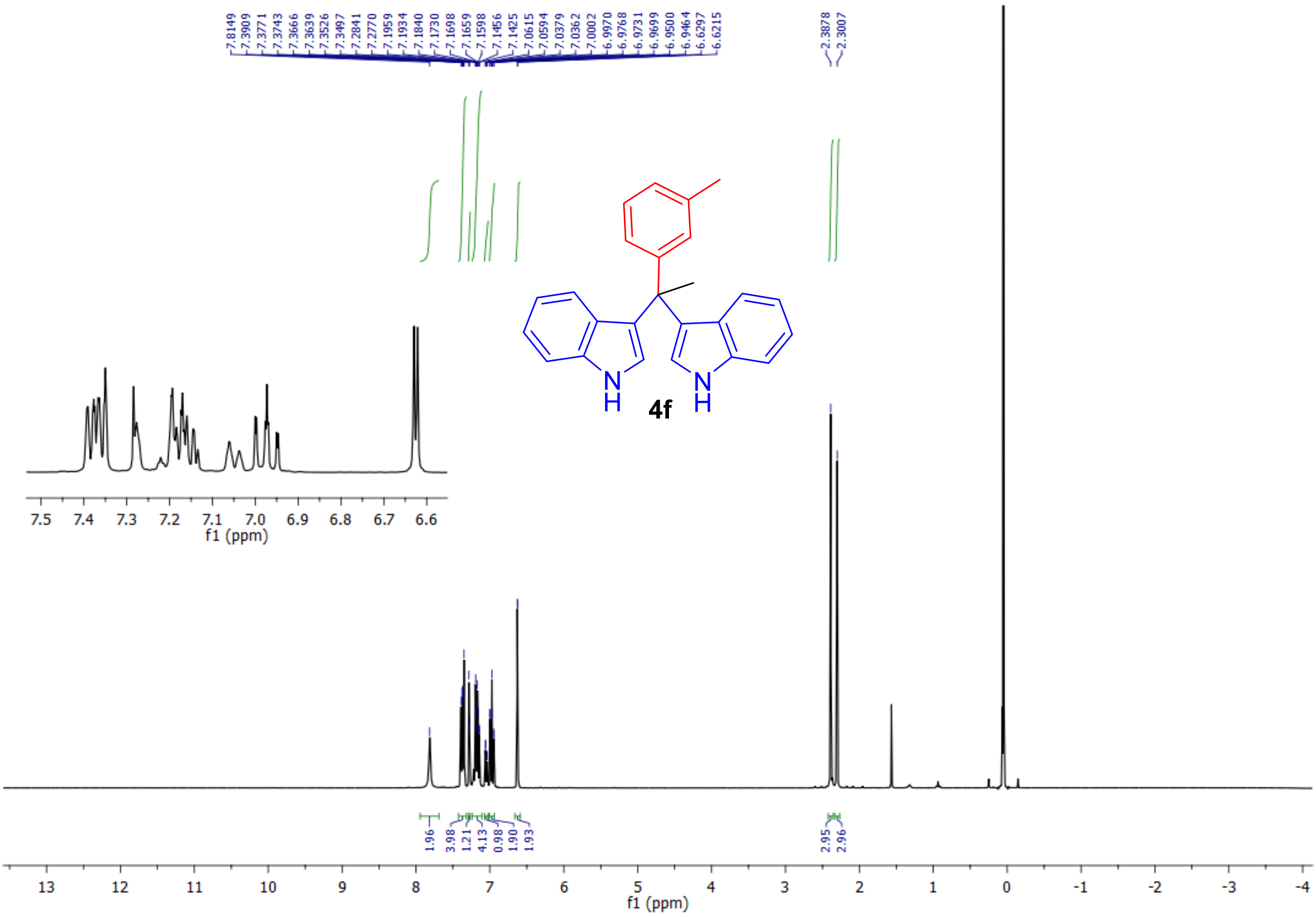

Figure S37. ^{1}H NMR Spectra of **4f** (300 MHz, $CDCl_3$)

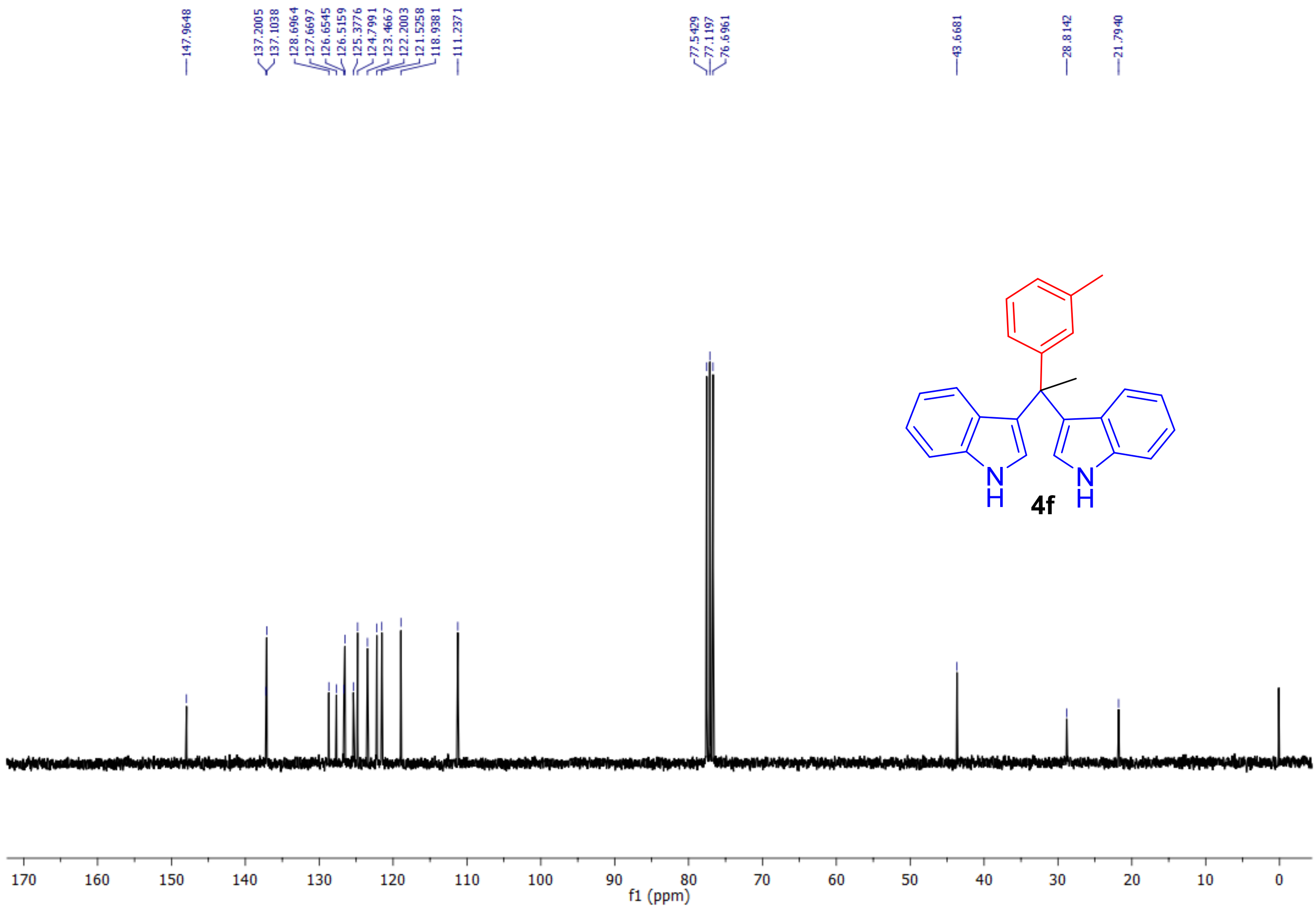

Figure S38. [13] C NMR Spectra of **4f** (300 MHz, $CDCl_3$)

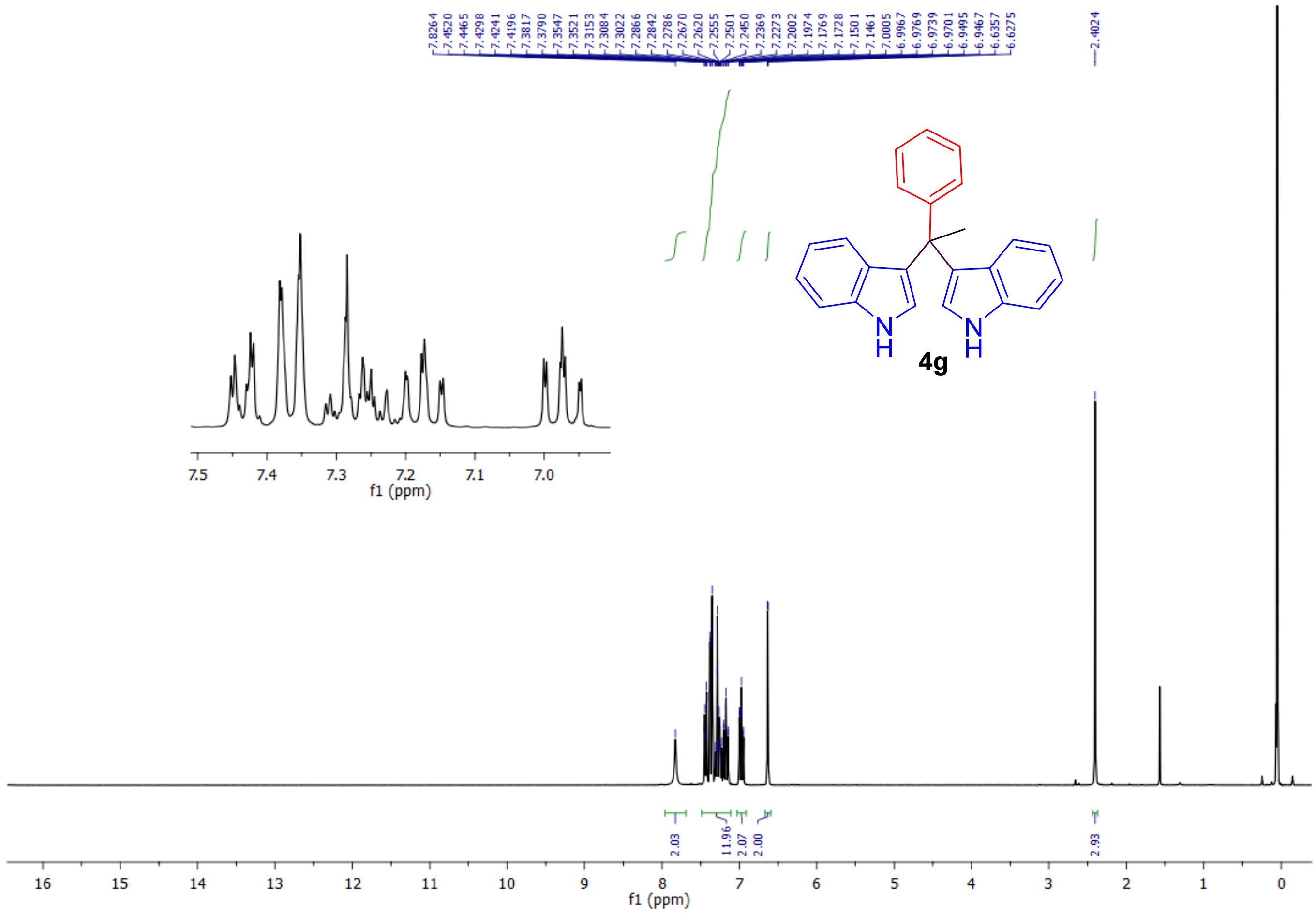

Figure S39. ^{1}H NMR Spectra of **4g** (300 MHz, $CDCl_3$)

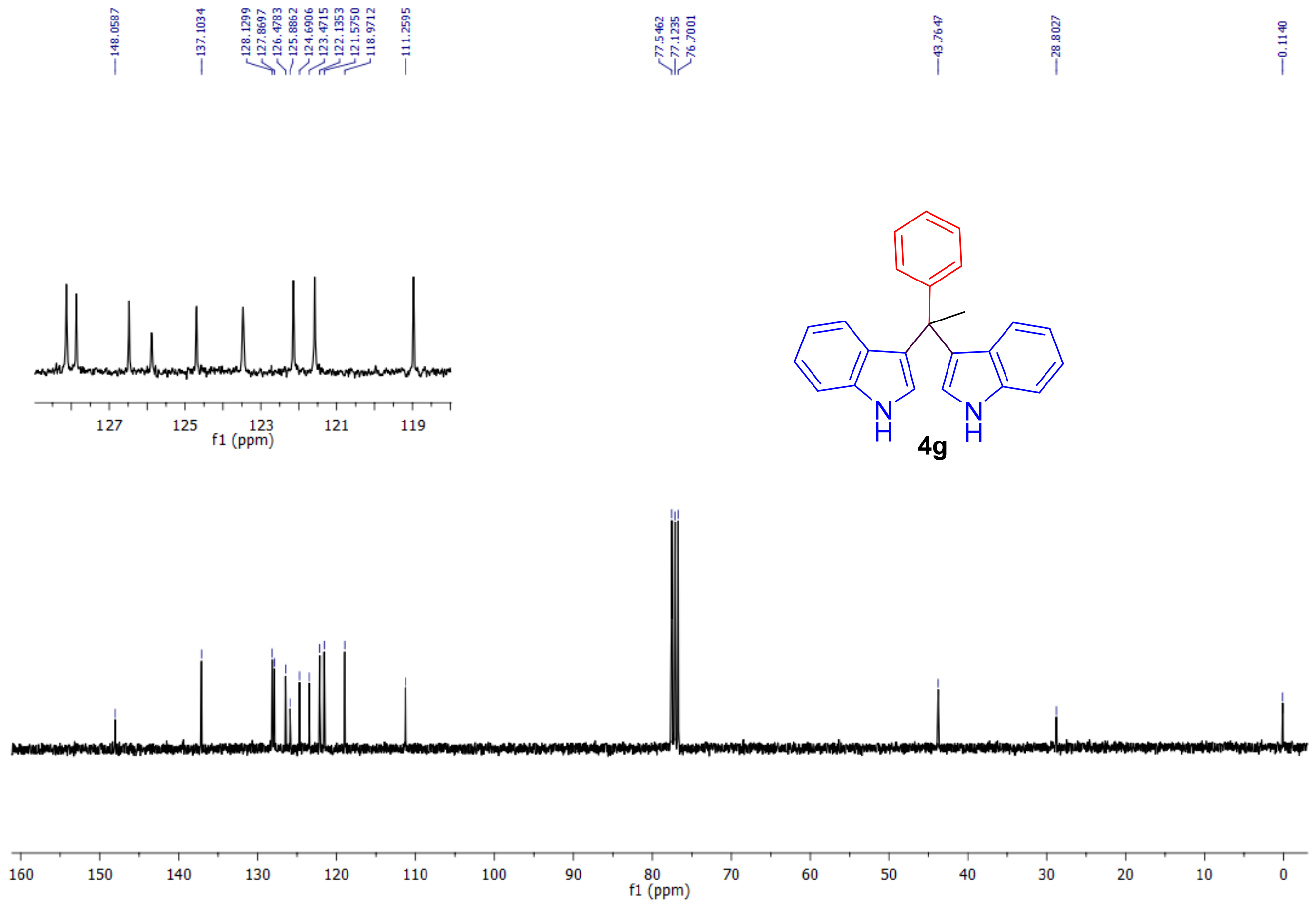

Figure S40. ^{13}C NMR Spectra of **4g** (300 MHz, $CDCl_3$)

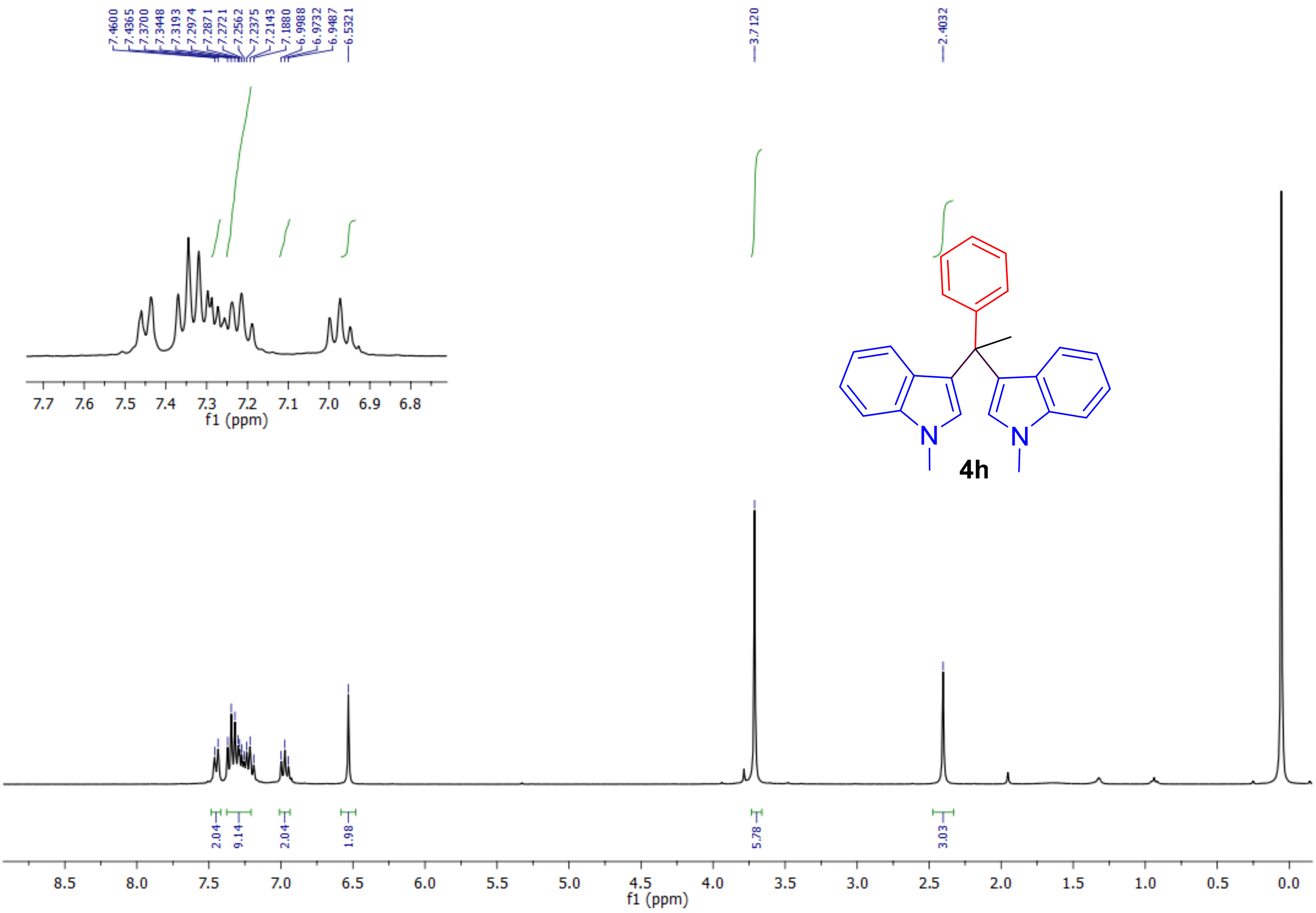

Figure S41. ^{1}H NMR Spectra of **4h** (300 MHz, $CDCl_3$)

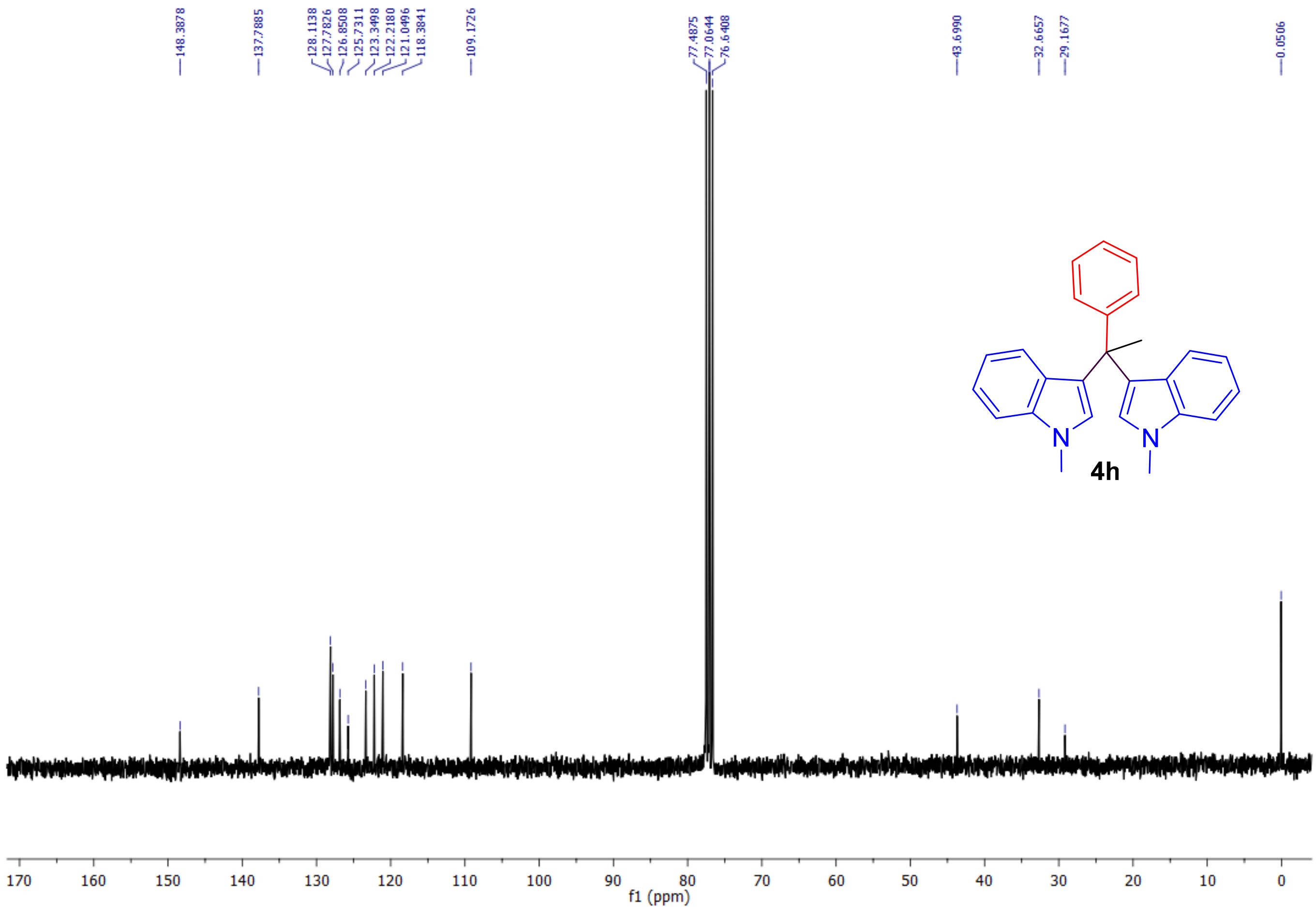

Figure S42. ^{13}C NMR Spectra of **4h** (300 MHz, $CDCl_3$)

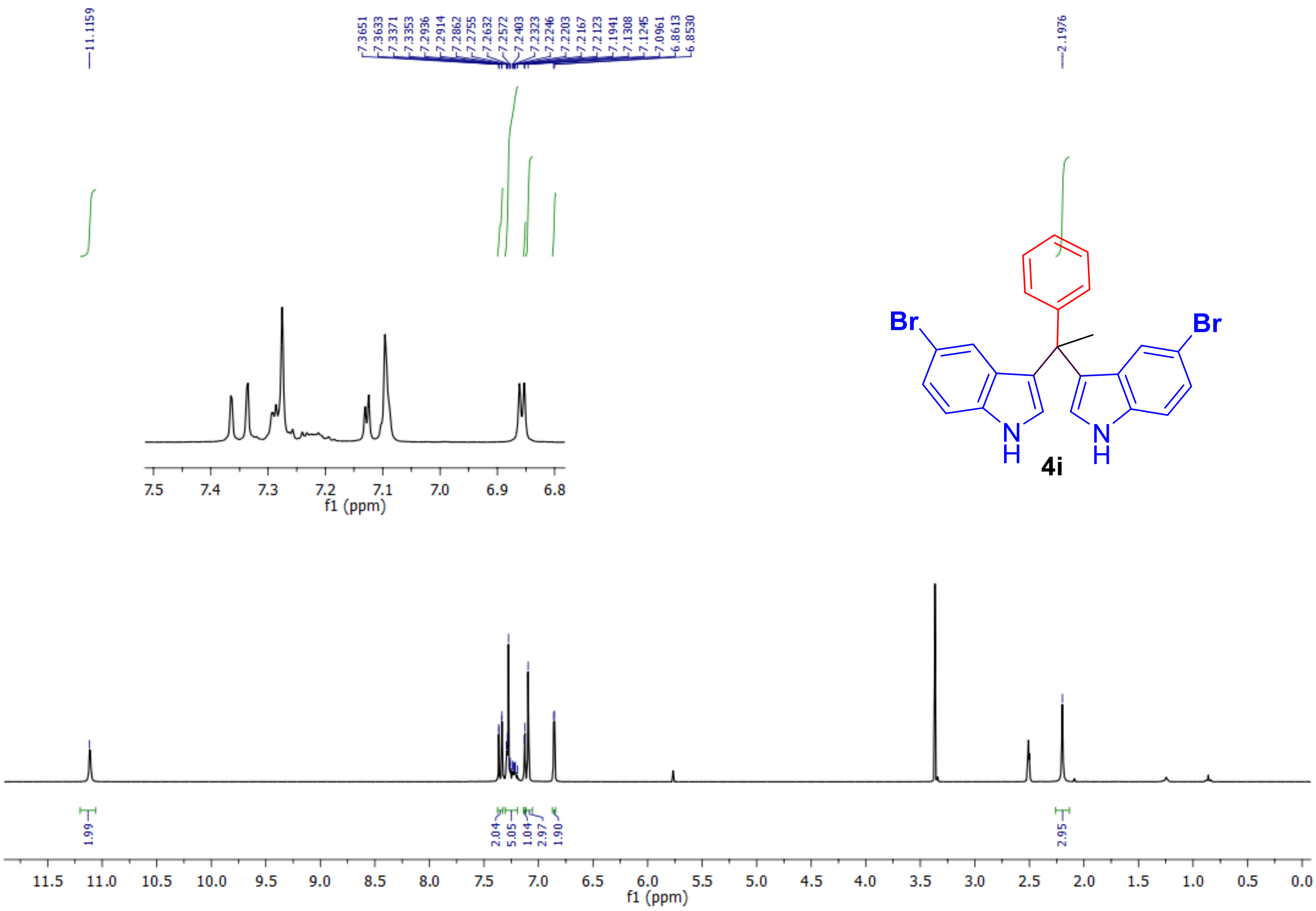

Figure S43. ^{1}H NMR Spectra of **4i** (300 MHz, DMSO-d_6)

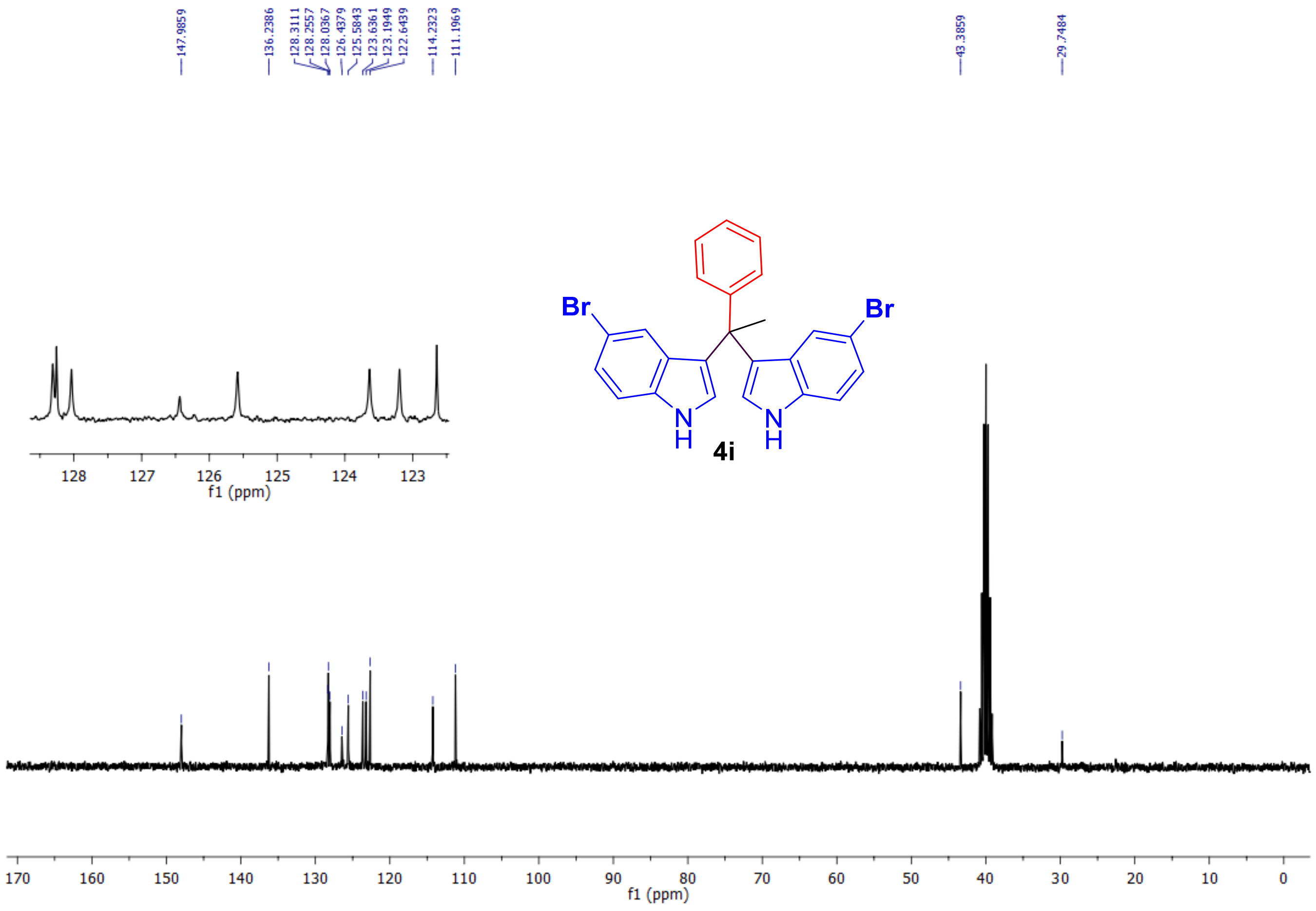

Figure S44. ^{13}C NMR Spectra of **4i** (300 MHz, $CDCl_3$)

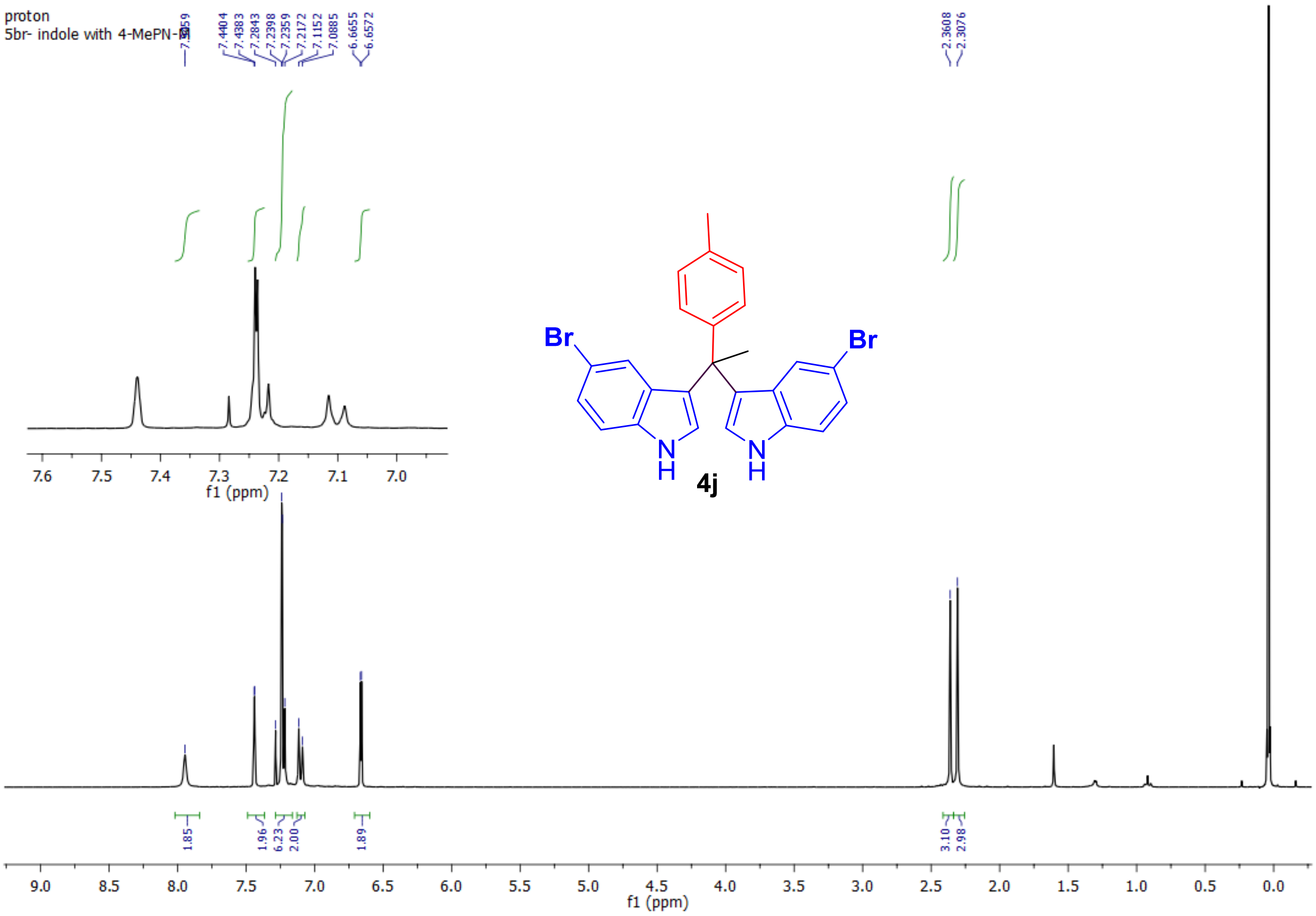

Figure S45. [1] H NMR Spectra of **4j** (300 MHz, $CDCl_3$)

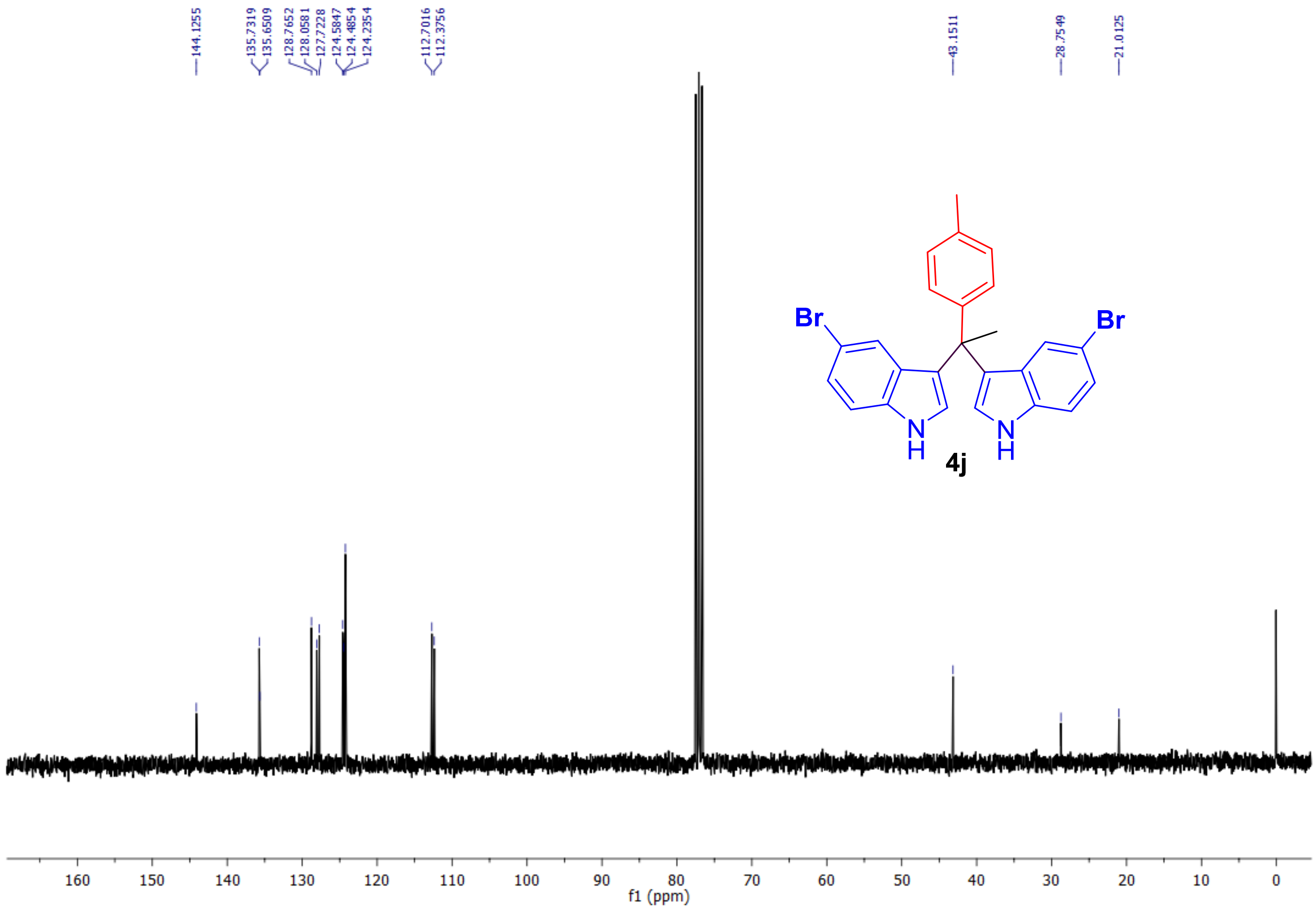

Figure S46. ^{13}C NMR Spectra of **4j** (300 MHz, $CDCl_3$)

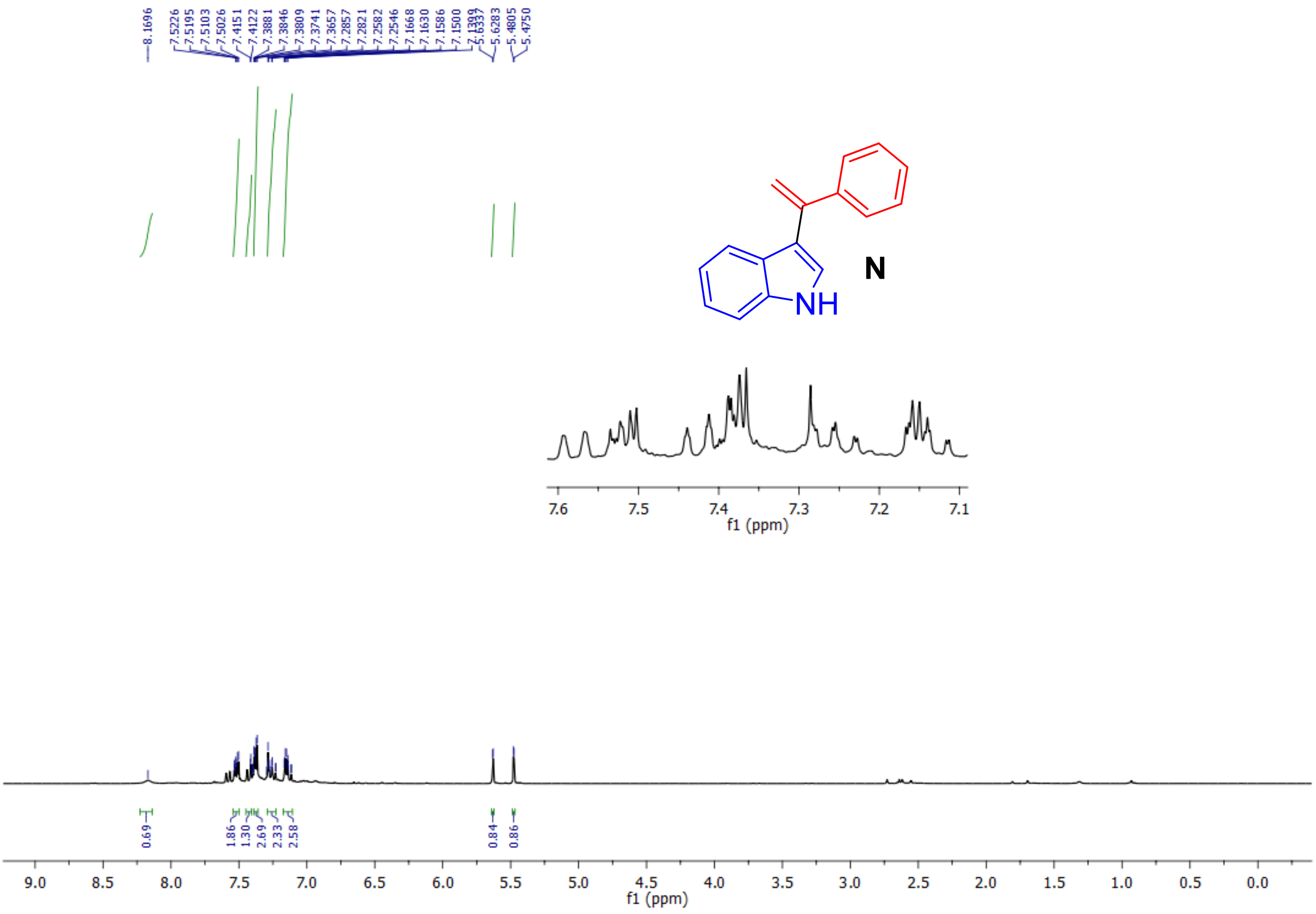

Figure S47. ^{1}H NMR Spectra of **N** (300 MHz, $CDCl_3$)

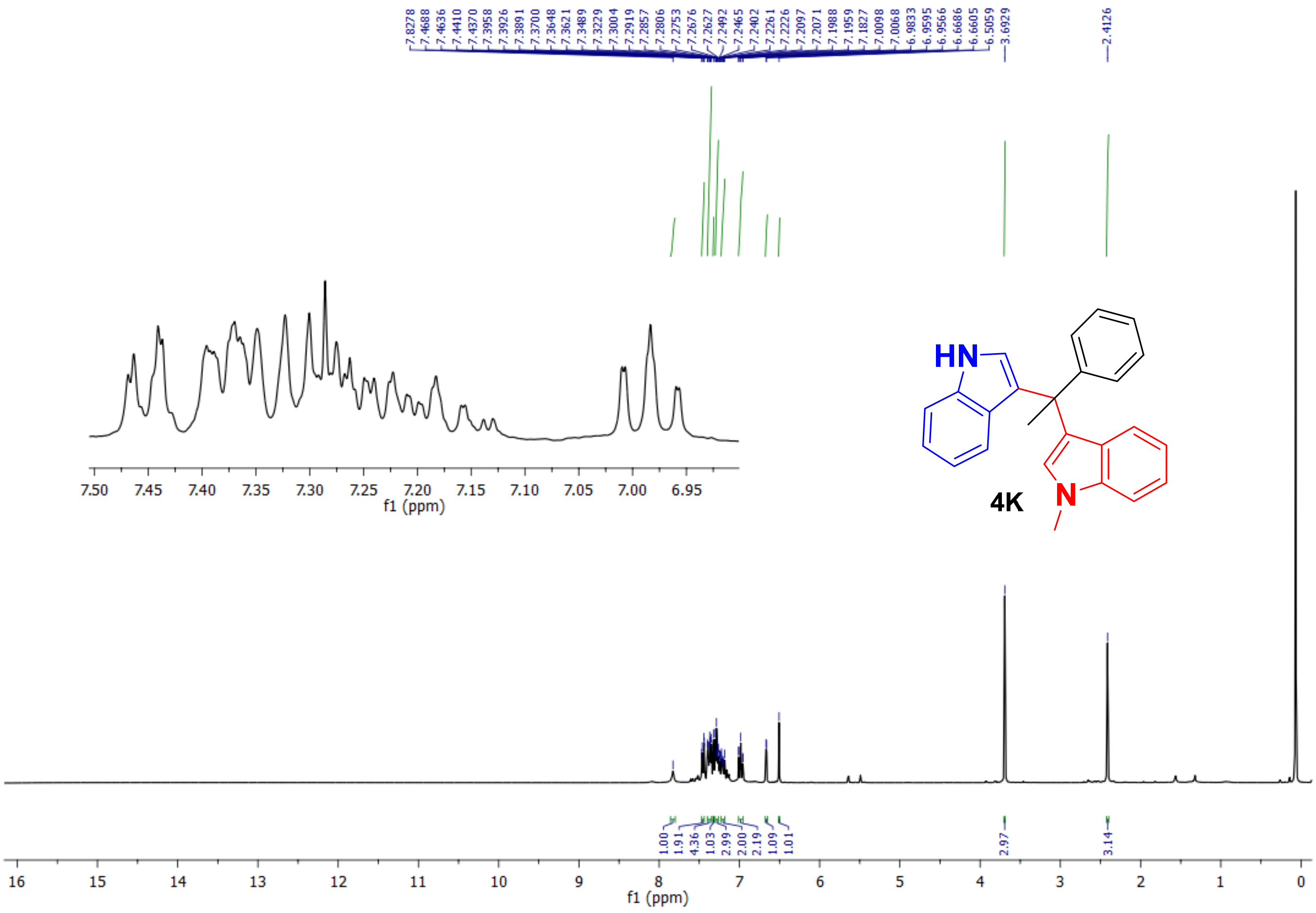

Figure S48. ^{1}H NMR Spectra of **4k** (300 MHz, $CDCl_3$)

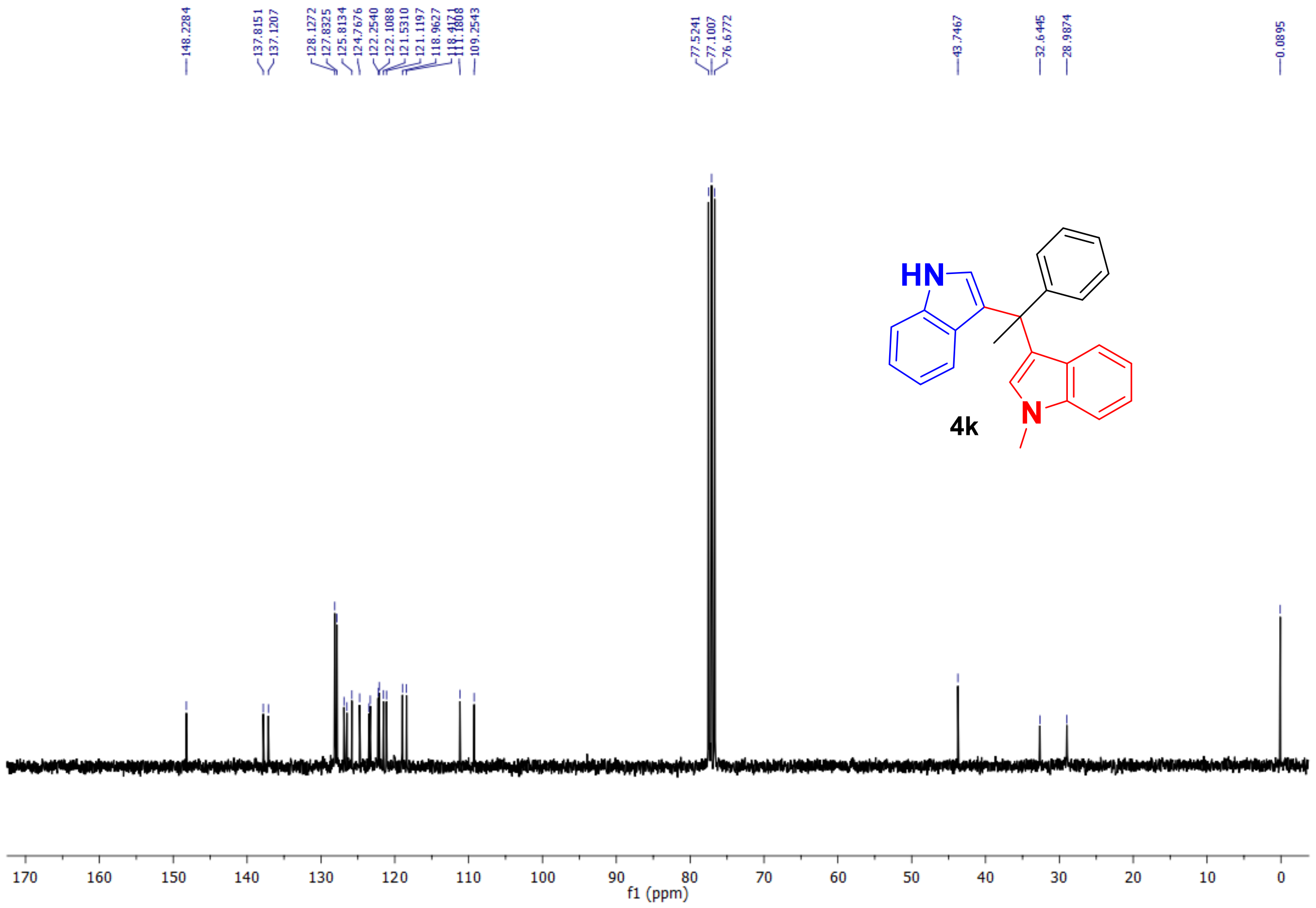

Figure S49. [13] C NMR Spectra of **4k** (300 MHz, $CDCl_3$)
